# Supplementary material for: Combining two genetic sexing strains allows sorting of non-transgenic males for Aedes genetic control
Source: Commun Biol. 2023 Jun 16;6:646. doi: 10.1038/s42003-023-05030-7 (PMC10275924; doi:10.1038/s42003-023-05030-7)
Supplement: Supplementary file 2 — Supplementary Information [file 42003_2023_5030_MOESM2_ESM.pdf]

Supplementary Information

Combining two Genetic Sexing Strains allows sorting of non-transgenic males for *Aedes* genetic control

Célia Lutrat, Myriam Burckbuchler, Roenick Proveti Olmo, Rémy Beugnon, Albin Fontaine, Omar S. Akbari, Rafael Argilés-Herrero, Thierry Baldet, Jérémy Bouyer, Eric Marois

List of Supplementary Tables:

Supplementary Table 1

Supplementary Table 2

Supplementary Table 3

List of Supplementary Data:

Supplementary Data 1

Supplementary Data 2

Supplementary Data 3

Supplementary Data 4

Supplementary Table 1: Summary and significance of statistical tests performed. More details can be found in **Supplementary Data 2**.

**Sex ratio (%)**		
**Pairwise comparison**	**Estimate (SE) ***	***p*-value**
Aaeg-M *vs*. Bra (WT)	-0.06 (0.09)	0.903
Aaeg-m *vs*. Bra (WT)	-0.01 (0.08)	0.999
Aaeg-CS *vs*. Bra (WT)	0.05 (0.09)	0.935
Aal-M *vs*. BiA (WT)	-0.09 (0.11)	0.847
Aal-m *vs*. BiA (WT)	-0.09 (0.12)	0.858
Aal-CS *vs*. BiA (WT)	-0.03 (0.11)	0.990
**Fecundity (%)**		
**Pairwise comparison**	**Estimate (SE) ****	***p*-value**
Zero hurdle models		
Aaeg-M *vs*. Bra (WT)	0.21 (0.65)	0.745
Aaeg-m *vs*. Bra (WT)	0.84 (0.77)	0.271
Aaeg-CS *vs*. Bra (WT)	0.61 (0.71)	0.386
Aal-M *vs*. BiA (WT)	0.52 (0.34)	0.626
Aal-m *vs*. BiA (WT)	-1.11 (0.65)	0.087 .
Aal-CS *vs*. BiA (WT)	-0.52 (0.68)	0.447
Truncated negative binomial models		
Aaeg-M *vs*. Bra (WT)	-0.20 (0.34)	0.551
Aaeg-m *vs*. Bra (WT)	-0.07 (0.34)	0.838
Aaeg-CS *vs*. Bra (WT)	0.61 (0.34)	0.073 .
Aal-M *vs*. BiA (WT)	0.52 (0.34)	0.126
Aal-m *vs*. BiA (WT)	0.41 (0.36)	0.252
Aal-CS *vs*. BiA (WT)	0.08 (0.35)	0.816
**Egg hatching rate (%)**		
**Pairwise comparison**	**Estimate (SE) ***	***p*-value**
Aaeg-M *vs*. Bra (WT)	-0.17 (0.10)	0.334

Aaeg-m *vs*. Bra (WT)	0.99 (0.11)	< 0.001 ***
Aaeg-CS *vs*. Bra (WT)	0.83 (0.10)	< 0.001 ***
Aal-M *vs*. BiA (WT)	0.15 (0.04)	0.002 **
Aal-m *vs*. BiA (WT)	-0.03 (0.06)	0.957
Aal-CS *vs*. BiA (WT)	0.10 (0.04)	0.046 *
**Larva to adult survival (%)**		
**Pairwise comparison**	**Estimate (SE)**	***p*-value**
Aaeg-M *vs*. Bra (WT)	-4.87 (3.83)	0.592
Aaeg-m *vs*. Bra (WT)	1.00 (4.22)	0.996
Aaeg-CS *vs*. Bra (WT)	3.75 (4.22)	0.831
Aal-M *vs*. BiA (WT)	18.13 (4.00)	0.003 **
Aal-m *vs*. BiA (WT)	16.63 (4.00)	0.006 **
Aal-CS *vs*. BiA (WT)	5.62 (4.00)	0.520
**Male competitiveness (%)**		
**Difference from expected**	**Estimate (SE) ***	***p*-value**
Aaeg-M *vs*. Bra (WT)	0.04 (0.04)	0.318
Aaeg-M *vs*. Aaeg-CS	0.08 (0.04)	0.057
Aal-M *vs*. BiA (WT)	-0.42 (0.07)	< 0.001
Aal-CS *vs*. BiA (WT)	0.05 (0.05)	0.34
**Flight test (%)**		
**Pairwise comparison**	**Estimate (SE) ***	***p*-value**
Aaeg-M *vs*. Bra (WT)	-0.22 (0.19)	0.461
Aaeg-CS *vs*. Bra (WT)	-0.05 (0.19)	0.966
Aal-M *vs*. BiA (WT)	-0.36 (0.18)	0.103
Aal-CS *vs*. BiA (WT)	0.35 (0.19)	0.148
**Male survival**		
**Pairwise comparison (7 days)**	**Estimate (SE)**	***p*-value**

Aaeg-M *vs*. Bra (WT)	0.00 (0.01)	0.764
Aaeg-CS *vs*. Bra (WT)	0.01 (0.01)	0.485
Aal-M *vs*. BiA (WT)	0.00 (0.02)	1.000
Aal-CS *vs*. BiA (WT)	0.01 (0.02)	0.722
**Pairwise comparison (14 days)**	**Estimate (SE)**	***p*-value**
Aaeg-M *vs*. Bra (WT)	0.00 (0.01)	0.976
Aaeg-CS *vs*. Bra (WT)	0.01 (0.01)	0.625
Aal-M *vs*. BiA (WT)	0.00 (0.02)	0.996
Aal-CS *vs*. BiA (WT)	0.00 (0.02)	0.980

* estimated difference in logit of individuals

Supplementary Table 2: Sequences of markers marking out and within the 63Mbp with high male-female genetic differentiation in *Ae. aegypti.*

137972_left_side_Xspe_163341238	GGAAAAAGGGTTTAAAAAATAACTGCATCAACCAACTATGCAAAAGCCAACTAGTACACACAGCTGACGTACAAACACTGCTGGAGACACTCAGCTTATTTTCCGCTGCAGGGTTATGTCATTGCAGGCAACACAT
220255_Xspe_166482560	TCGACCTTGGCTCGGAGTTTCCGTCGCGTTTGACCCAAAACCGGCGTACATAGGAAAGCATCCGAACCAAGGTGGTCAGATTCGTTGTAACCGGCGATACGGTCCATTTCGGCAATCGCCGAAACCGGTGTTTTGG
31870_right_side_Xspe_174626199	CAGCCAATCCGAAAAGCAAACACAAAGCCTGCCCGAGTTCGTCTGCTTTCCCGATGGGGTGCAATAGAGCAAAAGTTTCTCTTTTGTCTATGCGGTGATATTTTTGCGAAACTCGCGATAGAAAGCCTTTCCTGTG

Supplementary Table 3: Cumulative number of fluorescent and negative males and females between generations 3 and 6 in 11 *Ae. albopictus* lines carrying an M-linked transgene. Underlined numbers are observations that may correspond to recombination events or to the presence of additional, less sex-linked transgene insertions. Lines showing such events were thus discarded. The selected M-linked line (Aal-M) is the one named "4.4Y" in the table.

Line	Positive males	Negative males	Positive females	Negative females	Total
**SM1**	181	0	3	316	500
**SM2**	53	0	34	25	112
**SM3**	301	0	0	625	926
**SM5**	259	11	26	384	680
**SM6**	22	0	1	17	40
**SM7**	81	0	30	63	174
**SM11**	79	0	11	51	141
**SM12**	647	0	0	623	1270
**1.2R**	666	0	0	569	1235
**4.4Y = Aal-M**	905	0	0	813	1718
**5.2GR**	403	0	0	310	713
