# Supplementary material for: Combining two genetic sexing strains allows sorting of non-transgenic males for Aedes genetic control
Source: Commun Biol. 2023 Jun 16;6:646. doi: 10.1038/s42003-023-05030-7 (PMC10275924; doi:10.1038/s42003-023-05030-7)
Supplement: Supplementary file 3 — Description of Additional Supplementary Files [file 42003_2023_5030_MOESM3_ESM.pdf]

Description of Additional Supplementary Files

File name: Supplementary Data 1
Description: 1239bp *Ae. albopictus* genomic sequence flanking the Aal-M transposon and 1647 bp *Ae. albopictus* genomic sequence flanking the Aal-m transposon

File name: Supplementary Data 2
Description: Detailed statistical outputs

File name: Supplementary Data 3
Description: Production costs and initial parameters

File name: Supplementary Data 4
Description: Plasmid and genomic integration nucleotide sequences
