# Supplementary material for: Combining two genetic sexing strains allows sorting of non-transgenic males for Aedes genetic control
Source: Commun Biol. 2023 Jun 16;6:646. doi: 10.1038/s42003-023-05030-7 (PMC10275924; doi:10.1038/s42003-023-05030-7)
Supplement: Supplementary file 4 — Supplementary Data 1 [file 42003_2023_5030_MOESM4_ESM.pdf]

Supplementary Data 1: 1239bp *Ae. albopictus* genomic sequence flanking the Aal-M transposon and 1647 bp *Ae. albopictus* genomic sequence flanking the Aal-m transposon. Complete flanking sequences and detailed explanations can be found in Supplementary Data 4.

Aal-M:

TTAACCACGAATTCTTTTAGGGATTCCTCCAGAGTCCTACCAGAAATTCATTTAGGGATAT
CTCCAGGAAATCCTTGAGAAATTCGTCCAGGGATCCAGGAATTCCTCCAGAGTATCCTTC
AGGCATTCCGGTCAGAAATCTTTAAGGGGTTCATCCTCGGATTTGTTCAGCAATCCATCC
ATGAATTCATCAAGAATACGCTCTGCTGCTTCTGCAAATGAATTCTTTCAGAATTTCGTCC
TGTGATTCTTTCAGGGATATTTATTATTGAGATTATTTTTTCCGAGAAATCCTTCAAGAAG
TTATCCATTGATTTTTTTCAAGAACTCCTCCACAAGTTTTTTCAGGGATGTTTAGAGAGTG
TCCTTCAATGATTCTTCCAAAAAAATAGTCAAGTCACTCGTTTAGAAATTCCCTCGGCAAT
ACCTCAAGGATTCCCTTCAGCGATTGTTTTAGGACTTATCCTAGGGGTTTGTTCAAATATT
ACATAACGCTAAGGGGGAGAGAGGGGGTCTAGCTCTGTGTTACGATTCATACAACATTC
TTAAAGTTTTCATATAAAATTTGTTACGTGGGGGAGGGAGGGGGTCTTAAATTGTGAAAT
TTTGCGTTACGTAATATTTGAAAGAACCTTAGGATTTCTTCAGGGATTCCACCAGGGACC
TCCTTTCGAGTTTTTTTTTTGTAAAGATTCTTTCAGAAATTTCTCCAAGAAATTGCCTAAA
AATTTTCCAGGAATCTCTTAAGCAGTTCCTTCAGGGATTGTATCAGGAATTCCTATACCTA
ATAAATCTTTCAGGGATTCTTCAAAGCAACACGCATGTTACAGAAGTGTCACGGCAGTG
CGCAAGGTTTTGTTTTCGAAAAAGTCACTGTGACCTACTTTATACAAATAAGTACCTACA
TGCTTGAATCGCTTACAAACANTTTTTCCAGGGAATCTATCAGGAATTCACCCAGAGAA
AACTTTTTTGGAATTTTCCCAGGGTTTGTCCGACAATTACCATAAGAAAAAGTATCAGGA
GTTCCCCTAGGAAWTCCTTCTCAAATTCCTTCATTTTGAATATATTCTAGCGATTCGTCTA
GGAATCCCTTCAGGTGAATTTCAGGATTTTTTCAAGAACTTCATTATTTGCGAGTTCTCC
GGAAATACCTCAAGAATTTCTCCAGGGATTTCTTACAGGAATACCTCGAGGGTTTCCTCT
AGTGGTAGTTACAAGGTTTAACGAGAAGCCTTCA

Aal-m:

TTAAGCTAGTATTTACATTCTGGTTTCCGATTTAGTTTAATAAGAATCAATCCGCAAACAG
AGATAAGATTTTGTATCCTGGCCCGAGGTAGTGGCTCACAATGGCTCAACGCAGACCCA
AGCAACACAGAAAACATCTTTGGACCATCAGTATTTCCAAAGATGTTTTTTAAGTGAATT
ATAAGCGATTCAGAAAACATCTTGTGTTAATATCCAGCTCTAATTATGCTGATCGAAAACA
AACAACATCAAACATGTTATTGTCTATATCACGTATGAAATATGTTTATATAACTATCAGTG
AATCAAAATTCAACCATCGCGCAACAATAATGACAATGTCGCAACCTGTATTTGTTGCGA
CAAAACAACCCATGCGACATATGTTTGTCCCAACTTGAAAATAATGGGATTAACATCGTA
CACCACGAGTTTAGAGATTTTAAGCCTTGCATTGCGATTTTCGAACGGTTACTTCGAGTC
GGTAAACACTGCAACTCTGGTGAAGCTCTATCATCAAACAGTTTCGACTTTGGATTAGTA
ATAATTGATTATTCACGAGTTGAAAAGTACGCAACAAATTCAGCTTCCGTTTTGTCTGCA
ACAAAAAATTTCGAGATCATGTCATTATCTGTCACCAGTAGGGATAAAGACTATCCATCA
CATCTTCTTTGTTGCTTGTTTTGTGCCTCTTTTGTCTGACAATTCTCTTCACTGATAACTAT
TATATAACAGAAACATCAAGTTGCTAATTCAATCATATGTAAAAGCAAACGCGACAGAAC
AACAGAAAGAAGATAAAGATAAAAATAAACCAAAATGCAAACATTGTACCGTCTACCCC
CGATGGTTTGAACGACACCTCATGCAAACCAACGGGGTTCTTTTTTTAATTTGAACTTTT
AGTAACCCTGTGGACATCAACAAACGTACTTTTGGTAACCTTATTTGTTGTTTTGTTTTGA
TTCTGCGTTCCGTTTCACCGCGTTCCATGGGCAGAATGACGTTTGAACCATTTTTAGTTT
GAACGATGTGCAGATTGGCGGGGGTCAAATTAAAAAGTGTTCAGATTAGATGCTGTCAA

ACCAATGGGGGTAGACGGTATGCATTGATTTTAACCATTCCTAGGAGGCTCTGGTCGATG
GGGGAGAGTGAGTGGTTCAAGGGACCACGTCCCCCACGTCCGACGAACGTCCCCGCGC
TAATTAGGATCACCATACACAAATTTGGCCATCATCCTCAAGGCCAAAATGTACAGGCTG
CTGATCTACCAACTGGTCTGTCGAATACTATCCAGAAACACCGCGCAACTTGTATTCCGG
CCTATGTGAAAGAATCAGCATCCAACAGTCGGTGCTATTCCAACTCCAGCGTGAATTCCG
GCACTCAAACTTCACCTCTCTGCCGTGTGCAGCATAGTTGTCCCATGTTCCAAAACAGC
AAACTGAGAAAAACGCGTTTTAAGTTTCAGCATTGTTTCCATCTCTACGGACAAGTGTA
ATAAAAACACAAAATGAATCCACCATCATAGCGTTAGAATTGGTATTTATCATTGAAGTTT
GAACAAAAAGTTTCATAAACATACAATTTACTTTGATTACACGGTCCGACAAAAAATCA
ACTTTTGTTCTGCCCAGCTCTTAAATT
