# Supplementary material for: Combining two genetic sexing strains allows sorting of non-transgenic males for Aedes genetic control
Source: Commun Biol. 2023 Jun 16;6:646. doi: 10.1038/s42003-023-05030-7 (PMC10275924; doi:10.1038/s42003-023-05030-7)

Supplementary Data 2: Detailed statistical outputs

Contents

Initial settings **1**

R version . . . 1

Packages . . . 2

Plot common settings . . . 2

Figure 3 **3**

Sex ratio . . . 3

Hatching rate . . . 15

Fecundity . . . 27

Larva survival . . . 41

Figure 4 **51**

Competitiveness . . . 51

Flight test . . . 65

Adult survival . . . 77

Figure 5 **96**

Data . . . 96

Model . . . 96

Initial settings

R version

platform x86_64-w64-mingw32
arch x86_64
os mingw32
system x86_64, mingw32 major 4
minor 1.0
year 2021
svn rev 80317
language R
version.string R version 4.1.0 (2021-05-18) nickname Camp Pontanezen

Packages

```
set.seed(2501)

libs <- c(
  'plyr', 'dplyr', 'tidyr', 'stringr',
  'ggeffects', 'lme4', 'multcomp',
  'performance', 'reshape2', 'icenReg',
  'survminer', 'survtools', 'survival',
  'ggplot2', 'ggpubr', 'viridis', 'cowplot'
)
invisible(lapply(libs, library, character.only = T))
```

Versions:

plyr__1.8.6, dplyr__1.0.7, tidyr__1.1.3, stringr__1.4.0

ggeffects__1.0.2, lme4__1.1-26, multcomp__1.4-16

performance__0.7.0, reshape2__1.4.4, icenReg__2.0.15

survminer__0.4.9, survtools__0.1, survival__3.2-11

ggplot2__3.3.5, ggpubr__0.4.0, viridis__0.5.1

Plot common settings

```
# Panel background color
species.bg = c('Ae. aegypti' = 'white', 'Ae. albopictus' = 'grey')

# Aeg line colors
line.color.aeg = c('Bra (WT)' = "#24ff24", 'Aaeg-M' = "#004949",
                   'Aaeg-m' = "#009292", 'Aaeg-CS' = "#006ddb")

# Alb line colors
line.color.alb = c('BiA (WT)' = "#24ff24", 'Aal-M' = "#004949",
                   'Aal-m' = "#009292", 'Aal-CS' = "#006ddb")

# Set of colors
pal <- c('1' = "#000000", '2' = "#004949", '3' = "#009292",
         '4' = "#ff6db6", '5' = "#ffb6db", '6' = "#490092",
         '7' = "#006ddb", '8' = "#b66dff", '9' = "#6db6ff",
         '10' = "#b6dbff", '11' = "#920000", '12' = "#924900",
         '13' = "#db6d00", '14' = "#24ff24", '15' = "#ffff6d")
```

Figure 3

Sex ratio

Ae. aegypti

Data .

```
# Data measurements
sr.aeg.rep = read.csv('data/Sex-ratio_aeg.csv', header = T, sep = ';')
sr.aeg.rep$sr = sr.aeg.rep$Males / (sr.aeg.rep$Males + sr.aeg.rep$Females) * 100
sr.aeg.rep$Line = factor(sr.aeg.rep$Line,
                         levels = c('Bra (WT)', 'Aaeg-M', 'Aaeg-m' , 'Aaeg-CS'))
sr.aeg.rep
```

```
   Replicate     Line Males Females       sr
1          2 Bra (WT)    97     100 49.23858
2          3 Bra (WT)    97      87 52.71739
3          4 Bra (WT)   126      95 57.01357
4          1   Aaeg-M   562     512 52.32775
5          2   Aaeg-M   912     837 52.14408
6          3   Aaeg-M   592     580 50.51195
7          1   Aaeg-m  1567    1301 54.63738
8          2   Aaeg-m   320     309 50.87440
9          3   Aaeg-m  1827    1693 51.90341
10         1  Aaeg-CS  1089     916 54.31421
11         2  Aaeg-CS   383     288 57.07899
12         3  Aaeg-CS   660     582 53.14010
```

```
# Build binomial dataset
males.aeg =
  rbind(
    data.frame(replicate = sr.aeg.rep[1,1],
               treatment = sr.aeg.rep[1,2],
               result = rep(1, sr.aeg.rep[1,3])),
    data.frame(replicate = sr.aeg.rep[1,1],
               treatment = sr.aeg.rep[1,2],
               result = rep(0, sr.aeg.rep[1,4])))

for(i in 2:nrow(sr.aeg.rep)){
  males.aeg =
    rbind(
      males.aeg,
      data.frame(replicate = sr.aeg.rep[i,1],
                 treatment = sr.aeg.rep[i,2],
                 result = rep(1, sr.aeg.rep[i,3])),
      data.frame(replicate = sr.aeg.rep[i,1],
                 treatment = sr.aeg.rep[i,2],
                 result = rep(0, sr.aeg.rep[i,4]))
    )

}
males.aeg$treatment = factor(x = males.aeg$treatment,
```

```
                                levels = c('Bra (WT)', 'Aaeg-M', 'Aaeg-m' , 'Aaeg-CS'))
males.aeg$Species = "Ae. aegypti"

summary.sr.aeg = males.aeg %>% group_by(treatment) %>%
  dplyr::summarise(value = n(),
            N = max(replicate)) %>% data.frame() %>%
  mutate(pos = c(1, 2, 3,4))

head(males.aeg)
```

```
  replicate treatment result     Species
1         2  Bra (WT)      1 Ae. aegypti
2         2  Bra (WT)      1 Ae. aegypti
3         2  Bra (WT)      1 Ae. aegypti
4         2  Bra (WT)      1 Ae. aegypti
5         2  Bra (WT)      1 Ae. aegypti
6         2  Bra (WT)      1 Ae. aegypti
```

Model .

```
# Generalized linear model with Bernoulli distribution
sr.aeg = glm(formula = 'result ~ treatment',
             family = binomial,
             data = males.aeg)
summary(sr.aeg)
```

```
Call:
glm(formula = "result ~ treatment", family = binomial, data = males.aeg)

Deviance Residuals:
   Min      1Q  Median      3Q     Max
-1.253  -1.228   1.103   1.128   1.148

Coefficients:
                  Estimate Std. Error z value Pr(>|z|)
(Intercept)       0.126414   0.081677   1.548    0.122
treatmentAaeg-M  -0.057801   0.087599  -0.660    0.509
treatmentAaeg-m  -0.009136   0.085106  -0.107    0.915
treatmentAaeg-CS  0.050668   0.087750   0.577    0.564

(Dispersion parameter for binomial family taken to be 1)

    Null deviance: 21476  on 15531  degrees of freedom
Residual deviance: 21470  on 15528  degrees of freedom
AIC: 21478

Number of Fisher Scoring iterations: 3
```

```
# Pairwise comparison
pairewise.aeg = glht(sr.aeg, mcp(treatment="Tukey"))
```

```
summary(pairewise.aeg)
```

```
	 Simultaneous Tests for General Linear Hypotheses

Multiple Comparisons of Means: Tukey Contrasts


Fit: glm(formula = "result ~ treatment", family = binomial, data = males.aeg)

Linear Hypotheses:
                         Estimate Std. Error z value Pr(>|z|)
Aaeg-M - Bra (WT) == 0  -0.057801   0.087599  -0.660   0.9063
Aaeg-m - Bra (WT) == 0  -0.009136   0.085106  -0.107   0.9995
Aaeg-CS - Bra (WT) == 0  0.050668   0.087750   0.577   0.9347
Aaeg-m - Aaeg-M == 0     0.048666   0.039679   1.226   0.5924
Aaeg-CS - Aaeg-M == 0    0.108469   0.045071   2.407   0.0689 .
Aaeg-CS - Aaeg-m == 0    0.059804   0.040012   1.495   0.4216
---
Signif. codes:  0 '***' 0.001 '**' 0.01 '*' 0.05 '.' 0.1 ' ' 1
(Adjusted p values reported -- single-step method)
```

Replication

```
      Line N    n
1 Bra (WT) 4  602
2   Aaeg-M 3 3995
3   Aaeg-m 3 7017
4  Aaeg-CS 3 3918
```

Model fit quality

```
check_model(sr.aeg)
```

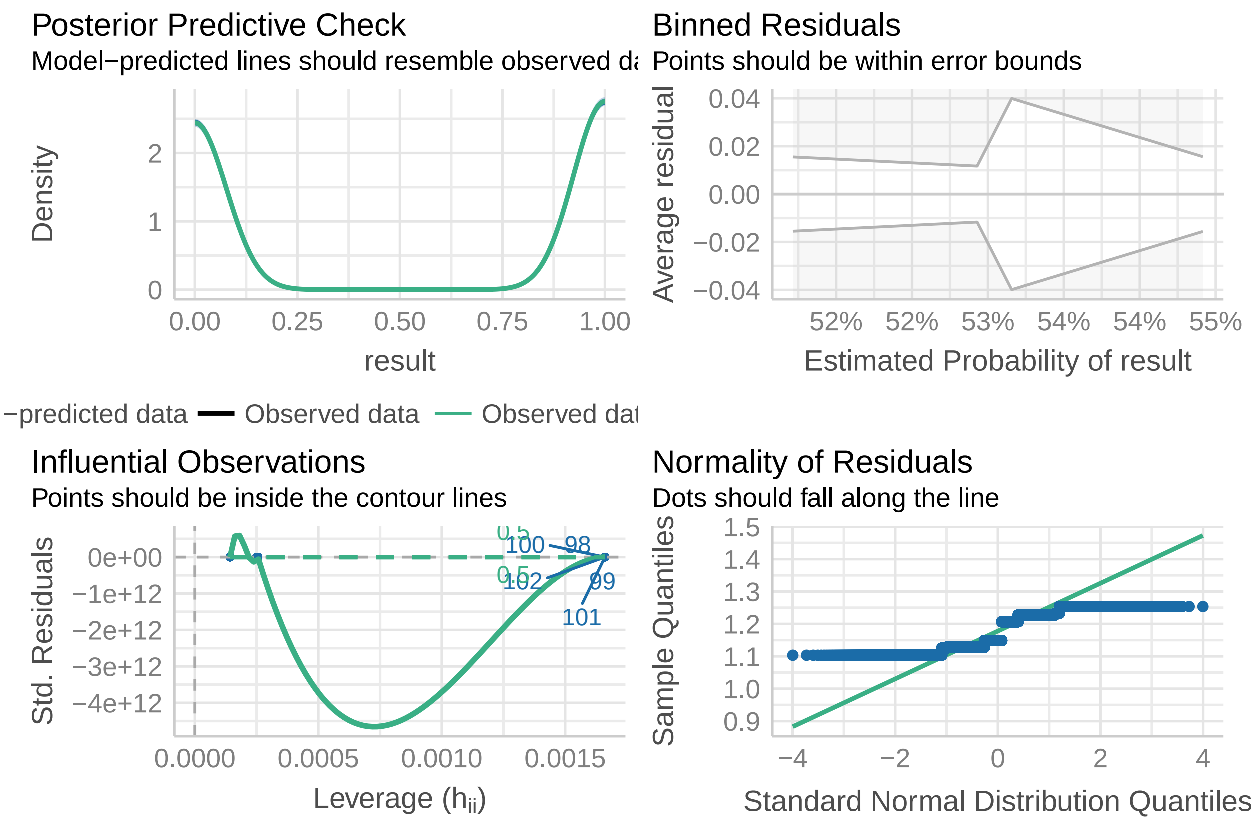

Plot .

```
# Extraction of the results
pred.sr.aeg = ggpredict(model = sr.aeg, terms = c("treatment")) %>% data.frame()
pred.sr.aeg$Species = "Ae. aegypti"


# plot
sr.plot.aeg =
  ggplot(data =  NULL) +
  geom_point(data = sr.aeg.rep,
             aes(x = jitter(as.numeric(factor(Line)),
                                 factor = .5),
                 y = sr , color = Line),
             alpha = .6, size = 2) +
  geom_point(data = pred.sr.aeg,
             aes(x = as.numeric(x),
                 y = predicted * 100),
             color = 'black',
             size = 1.5) +
  geom_errorbar(data = pred.sr.aeg,
                aes(x = as.numeric(x),
                    ymin = conf.low* 100,
                    ymax = conf.high* 100),
                color = 'black',
```

```
               width = 0.05) +
  annotate(geom = 'text',
           x = summary.sr.aeg$pos, y = 15,
           label = 'N = 3',
           size = 4) +
  annotate(geom = 'text',
           x = summary.sr.aeg$pos, y = 7,
           label = paste0('n = ', summary.sr.aeg$value),
           size = 3.5) +
  annotate(geom = 'line', x = 1:4, y = 90) +
  annotate(geom = 'text', x = 2.5, y = 95,
           label = 'n.s.',
           size = 4) +
  annotate(geom = 'line', x = 1:3, y = 80) +
  annotate(geom = 'text', x = 2, y = 85,
           label = 'n.s.',
           size = 4) +
  annotate(geom = 'line', x = 1:2, y = 70) +
  annotate(geom = 'text', x = 1.5, y = 75,
           label = 'n.s.',
           size = 4) +
  scale_x_continuous(breaks = 1:4,
                     labels = c("Bra (WT)",
                                "Aaeg-M",
                                "Aaeg-m",
                                "Aaeg-CS"),
                     limits = c(.5,4.5), guide = guide_axis(angle = 30)) +
  scale_y_continuous(breaks = c(0, 50, 100), limits = c(0, 100)) +
  scale_color_manual(values = line.color.aeg, labels = names(line.color.aeg)) +
  labs(x = 'Line',
       y = 'Percentage of males (%)') +
  theme_classic() +
  theme(panel.background =
          element_rect(fill = species.bg[names(species.bg)=="Ae. aegypti"]),
        legend.position = 'none',
        axis.title.y = element_text(color = 'black',
                                    size = 12),
        axis.text.y = element_text(size = 10,
                                   hjust = 0, vjust = 0),
        axis.title.x = element_text(color = 'black',
                                    size = 12),
        axis.text.x = element_text(size = 10))

sr.plot.aeg
```

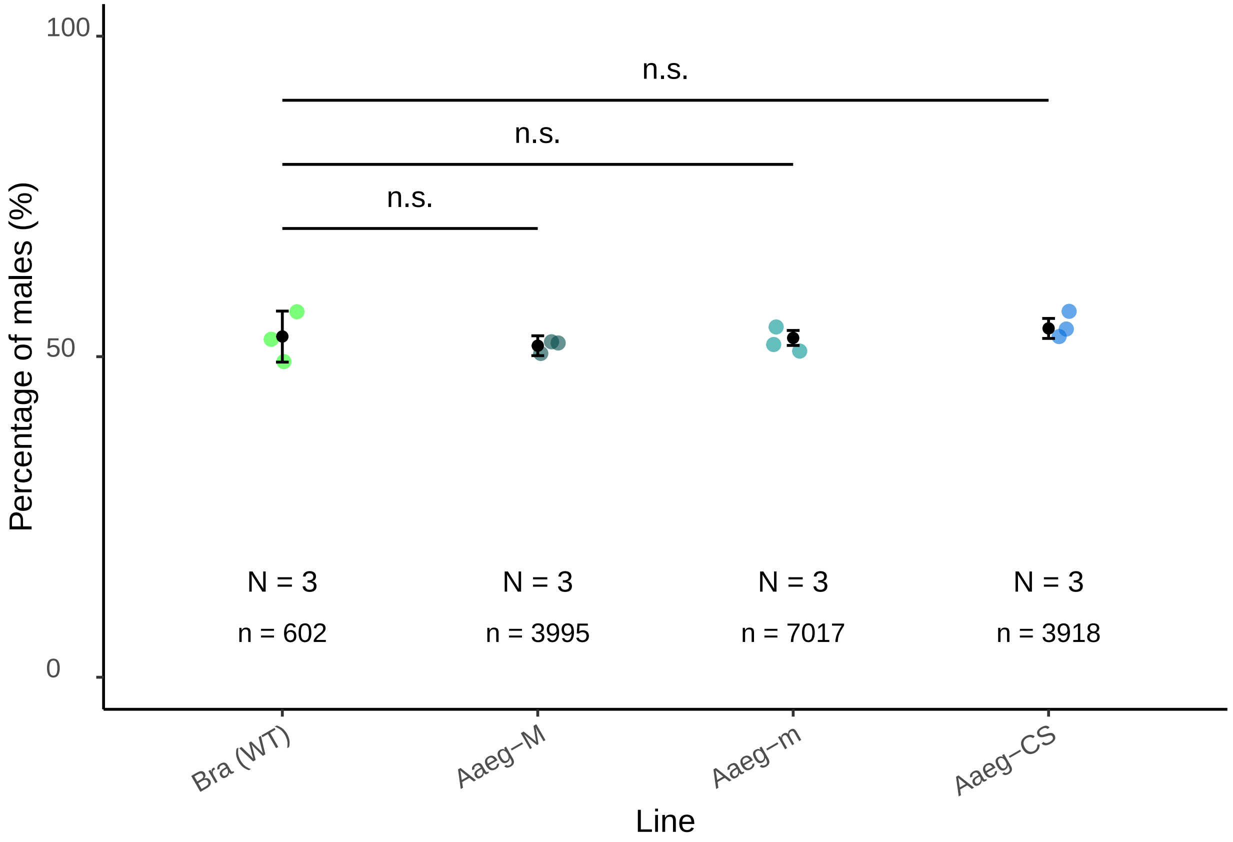

Percentage of males (%)
100
50
0
n.s.
n.s.
n.s.
N = 3
n = 602
N = 3
n = 3995
N = 3
n = 7017
N = 3
n = 3918
Bra (WT)
Aaeg–M
Aaeg–m
Aaeg–CS
Line

Ae. albopictus

Data .

```
# Data measurements
sr.alb.rep = read.table("data/Sex-ratio_albo.csv", header=T, sep=";")
sr.alb.rep$sr = sr.alb.rep$Males /
  (sr.alb.rep$Males + sr.alb.rep$Females) * 100
sr.alb.rep$Line = factor(sr.alb.rep$Line,
                         levels = c('BiA (WT)', 'Aal-M', 'Aal-m', 'Aal-CS'))
sr.alb.rep
```

```
   Replicate     Line Males Females       sr
1          A    Aal-m   167     166 50.15015
2          B    Aal-m   245     246 49.89817
3          C    Aal-m   293     274 51.67549
4          D   Aal-CS   136     119 53.33333
5          E   Aal-CS   533     493 51.94932
6          F   Aal-CS   510     471 51.98777
7          J    Aal-M   232     223 50.98901
8          K    Aal-M   553     537 50.73394
9          L    Aal-M   184     183 50.13624
10         1 BiA (WT)    47      45 51.08696
11         2 BiA (WT)    74      67 52.48227
12         3 BiA (WT)    76      63 54.67626
```

```
# Build binomial dataset
males.albo =
  rbind(
    data.frame(replicate = sr.alb.rep[1,1],
               treatment = sr.alb.rep[1,2],
               result = rep(1, sr.alb.rep[1,3])),
    data.frame(replicate = sr.alb.rep[1,1],
               treatment = sr.alb.rep[1,2],
               result = rep(0, sr.alb.rep[1,4])))

for(i in 2:nrow(sr.alb.rep)){
  males.albo =
    rbind(
      males.albo,
      data.frame(replicate = sr.alb.rep[i,1],
                 treatment = sr.alb.rep[i,2],
                 result = rep(1, sr.alb.rep[i,3])),
      data.frame(replicate = sr.alb.rep[i,1],
                 treatment = sr.alb.rep[i,2],
                 result = rep(0, sr.alb.rep[i,4]))
    )

}
males.albo$treatment = factor(males.albo$treatment,
                              levels = c('BiA (WT)', 'Aal-M', 'Aal-m', 'Aal-CS'))
males.albo$Species = "Ae. albopictus"
```

```
summary.sr.alb = males.albo %>%
  group_by(treatment) %>%
  dplyr::summarise(value = n()) %>%
  data.frame() %>%
  mutate(pos = c(1, 2, 3,4))

head(males.albo)
```

```
  replicate treatment result        Species
1         A     Aal-m      1 Ae. albopictus
2         A     Aal-m      1 Ae. albopictus
3         A     Aal-m      1 Ae. albopictus
4         A     Aal-m      1 Ae. albopictus
5         A     Aal-m      1 Ae. albopictus
6         A     Aal-m      1 Ae. albopictus
```

Model .

```
# Generalized linear model with Bernoulli distribution
sr.albo = glm(formula = 'result ~ treatment',
              family = binomial,
              data = males.albo)
summary(sr.albo)
```

```
Call:
glm(formula = "result ~ treatment", family = binomial, data = males.albo)

Deviance Residuals:
   Min      1Q  Median      3Q     Max
-1.228  -1.189   1.128   1.166   1.166

Coefficients:
                Estimate Std. Error z value Pr(>|z|)
(Intercept)      0.11842    0.10388   1.140    0.254
treatmentAal-M  -0.09122    0.11350  -0.804    0.422
treatmentAal-m  -0.09110    0.11690  -0.779    0.436
treatmentAal-CS -0.03349    0.11208  -0.299    0.765

(Dispersion parameter for binomial family taken to be 1)

    Null deviance: 8226.0  on 5936  degrees of freedom
Residual deviance: 8224.4  on 5933  degrees of freedom
AIC: 8232.4

Number of Fisher Scoring iterations: 3
```

```
# Pairwise comparison
pairewise.albo = glht(sr.albo, mcp(treatment="Tukey"))

summary(pairewise.albo)
```

```
     Simultaneous Tests for General Linear Hypotheses

Multiple Comparisons of Means: Tukey Contrasts


Fit: glm(formula = "result ~ treatment", family = binomial, data = males.albo)

Linear Hypotheses:
                         Estimate Std. Error z value Pr(>|z|)
Aal-M - BiA (WT) == 0  -0.0912194  0.1135026  -0.804    0.847
Aal-m - BiA (WT) == 0  -0.0910976  0.1169041  -0.779    0.858
Aal-CS - BiA (WT) == 0 -0.0334861  0.1120801  -0.299    0.990
Aal-m - Aal-M == 0      0.0001218  0.0704883   0.002    1.000
Aal-CS - Aal-M == 0     0.0577333  0.0621609   0.929    0.781
Aal-CS - Aal-m == 0     0.0576115  0.0681741   0.845    0.826
(Adjusted p values reported -- single-step method)
```

Replication

```
# A tibble: 4 x 3
  Line         N     n
  <fct>    <int> <int>
1 BiA (WT)     3   372
2 Aal-M        3  1912
3 Aal-m        3  1391
4 Aal-CS       3  2262
```

Model fit quality

```
check_model(sr.albo)
```

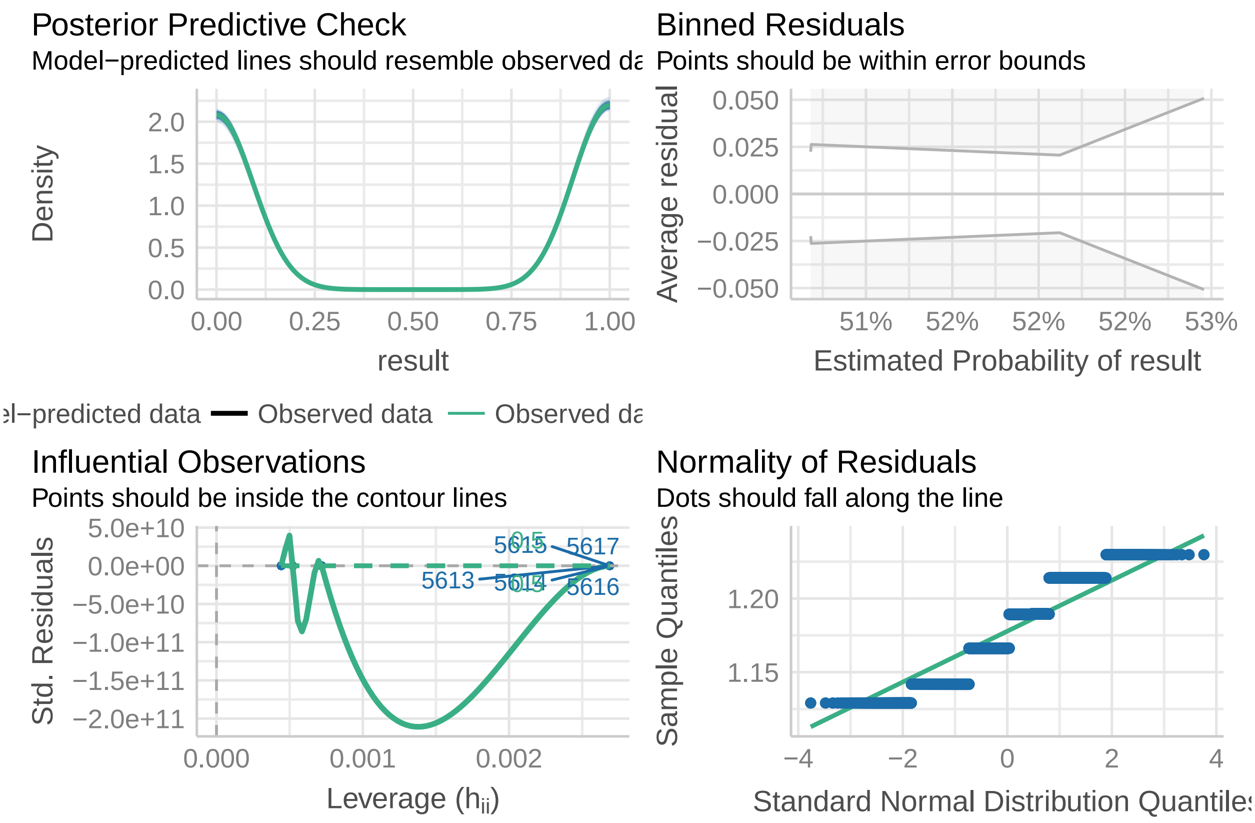

Plot .

```
# Extraction of the results
pred.sr.albo = ggpredict(model = sr.albo,
                         terms = c("treatment")) %>%
  data.frame()
pred.sr.albo$Species = "Ae. albopictus"


# plot
sr.plot.albo =
  ggplot(data =  NULL) +
  geom_point(data = sr.alb.rep,
             aes(x = jitter(as.numeric(factor(Line)),
                            factor = .5),
                 y = sr ,
                 color = Line),
             alpha = .6,
             size = 2) +
  geom_point(data = pred.sr.albo,
             aes(x = as.numeric(x),
                 y = predicted * 100),
             size = 1.5, color = 'black') +
  geom_errorbar(data = pred.sr.albo,
                aes(x = as.numeric(x),
```

```
                    ymin = conf.low* 100,
                    ymax = conf.high* 100),
                width = 0.05,
                color = 'black') +
  annotate(geom = 'text',
           x = summary.sr.alb$pos, y = 15,
           label = 'N = 3',
           size = 4) +
  annotate(geom = 'text',
           x = summary.sr.alb$pos, y = 7,
           label = paste0('n = ', summary.sr.alb$value),
           size = 3.5) +
  annotate(geom = 'line', x = 1:4, y = 90) +
  annotate(geom = 'text', x = 2.5, y = 95,
           label = 'n.s.',
           size = 4) +
  annotate(geom = 'line', x = 1:3, y = 80) +
  annotate(geom = 'text', x = 2, y = 85,
           label = 'n.s.',
           size = 4) +
  annotate(geom = 'line', x = 1:2, y = 70) +
  annotate(geom = 'text', x = 1.5, y = 75,
           label = 'n.s.',
           size = 4) +
  scale_x_continuous(breaks = 1:4,
                     labels = c("BiA (WT)",
                                "Aal-M",
                                "Aal-m",
                                "Aal-CS"),
                     limits = c(.5,4.5),
                     guide = guide_axis(angle = 30)) +
  scale_y_continuous(breaks = c(0, 50, 100), limits = c(0, 100)) +
  scale_color_manual(values = line.color.alb,
                     labels = names(line.color.alb)) +
  labs(x = 'Line',
       y = 'Percentage of males (%)') +
  theme_classic() +
  theme(panel.background =
          element_rect(fill = species.bg[names(species.bg)=="Ae. albopictus"]),
        legend.position = 'none',
        axis.title.y = element_text(color = 'black',
                                    size = 12),
        axis.text.y = element_text(size = 10,
                                   hjust = 0, vjust = 0),
        axis.title.x = element_text(color = 'black',
                                    size = 12),
        axis.text.x = element_text(size = 10))

sr.plot.albo
```

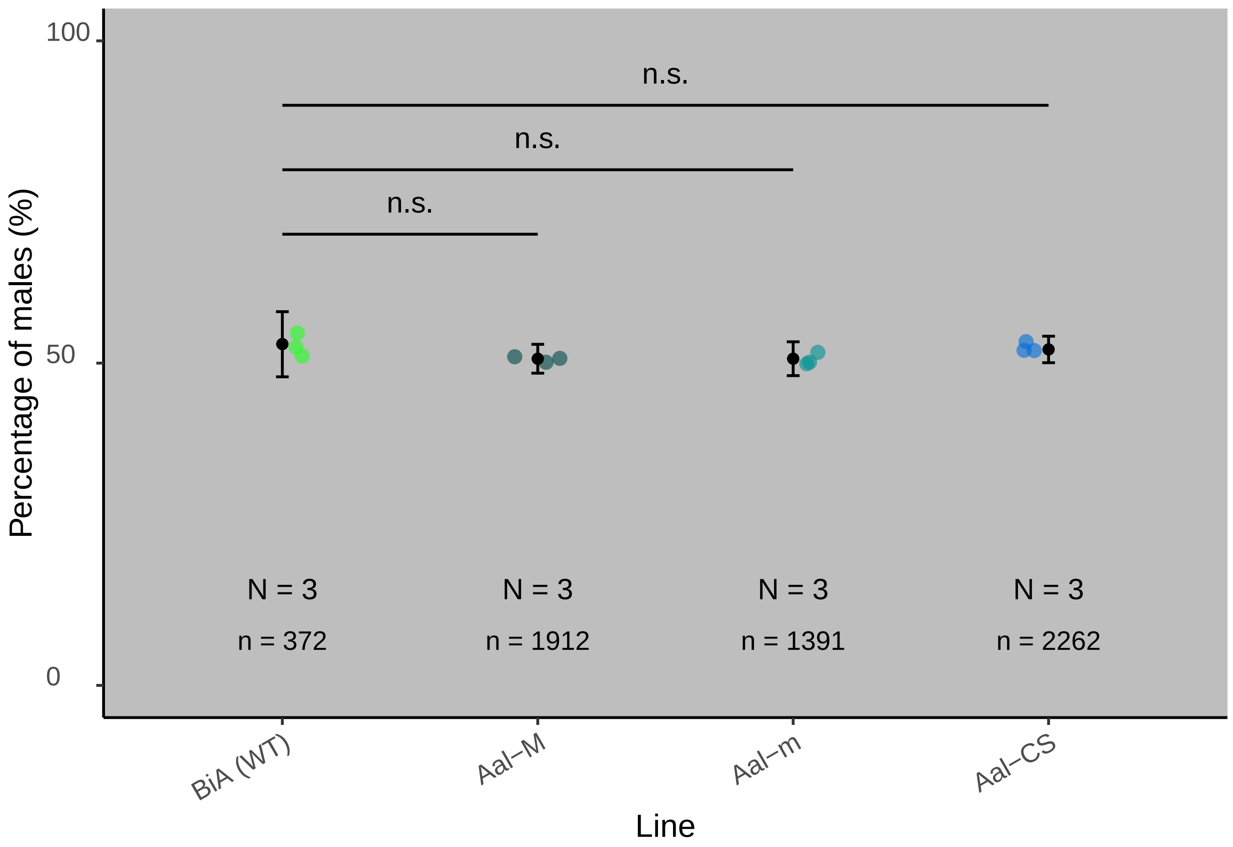
n.s.
n.s.
n.s.
100
50
0
Percentage of males (%)
N = 3
n = 372
N = 3
n = 1912
N = 3
n = 1391
N = 3
n = 2262
BiA (WT)
Aal–M
Aal–m
Aal–CS
Line

Hatching rate

Ae. aegypti

Data .

```
# Data measurements
hr.aeg = read.table("data/H-rate_aeg.csv",
                    header = T, sep = ";")
hr.aeg$hr = hr.aeg$Larvae/hr.aeg$Eggs * 100
hr.aeg$Line = factor(hr.aeg$Line, levels = c("Bra (WT)",
                                              "Aaeg-M",
                                              "Aaeg-m",
                                              "Aaeg-CS"))
hr.aeg
```

```
   Replicate     Line Eggs Larvae Unhatched       hr
1          1 Bra (WT)  220    140        80 63.63636
2          2 Bra (WT)  235    141        94 60.00000
3          3 Bra (WT)  272    166       106 61.02941
4          1   Aaeg-M  435    259       176 59.54023
5          2   Aaeg-M  297    157       140 52.86195
6          3   Aaeg-M  291    172       119 59.10653
7          1   Aaeg-m  392    315        77 80.35714
8          2   Aaeg-m  295    242        53 82.03390
9          3   Aaeg-m  491    398        93 81.05906
10         1  Aaeg-CS  515    405       110 78.64078
11         2  Aaeg-CS  335    283        52 84.47761
12         3  Aaeg-CS  465    346       119 74.40860
```

```
# Build binomial dataset
hatch.aegypti =
  rbind(
    data.frame(replicate = hr.aeg[1,1],
               treatment = hr.aeg[1,2],
               result = rep(0, hr.aeg[1,5])),
    data.frame(replicate = hr.aeg[1,1],
               treatment = hr.aeg[1,2],
               result = rep(1, hr.aeg[1,4])))

for(i in 2:nrow(hr.aeg)){
  hatch.aegypti =
    rbind(
      hatch.aegypti,
      data.frame(replicate = hr.aeg[i,1],
                 treatment = hr.aeg[i,2],
                 result = rep(0, hr.aeg[i,5])),
      data.frame(replicate = hr.aeg[i,1],
                 treatment = hr.aeg[i,2],
                 result = rep(1, hr.aeg[i,4]))
    )

}
```

```r
hatch.aegypti$treatment=factor(hatch.aegypti$treatment,
                               levels = c("Bra (WT)", "Aaeg-M", "Aaeg-m", "Aaeg-CS"))
hatch.aegypti$Species = 'Ae. aegypti'

summary.hatch.aeg = hatch.aegypti %>%
  group_by(treatment) %>%
  dplyr::summarise(value = n()) %>%
  data.frame() %>%
  mutate(pos = c(1, 2, 3, 4))

head(hatch.aegypti)
```

```
  replicate treatment result     Species
1         1  Bra (WT)      0 Ae. aegypti
2         1  Bra (WT)      0 Ae. aegypti
3         1  Bra (WT)      0 Ae. aegypti
4         1  Bra (WT)      0 Ae. aegypti
5         1  Bra (WT)      0 Ae. aegypti
6         1  Bra (WT)      0 Ae. aegypti
```

Model .

```r
# Generalized linear model with Bernoulli distribution
hatch.aegypti_stat = glm(formula = 'result ~ treatment',
                        family = binomial,
                        data = hatch.aegypti)
summary(hatch.aegypti_stat)
```

```
Call:
glm(formula = "result ~ treatment", family = binomial, data = hatch.aegypti)

Deviance Residuals:
    Min       1Q   Median       3Q      Max
-1.8245  -1.3078   0.6479   0.6934   1.0524

Coefficients:
                 Estimate Std. Error z value Pr(>|z|)
(Intercept)       0.46777    0.07621   6.138 8.38e-10 ***
treatmentAaeg-M  -0.16639    0.09904  -1.680   0.0929 .
treatmentAaeg-m   0.98677    0.10649   9.266  < 2e-16 ***
treatmentAaeg-CS  0.83507    0.10166   8.214  < 2e-16 ***
---
Signif. codes:  0 '***' 0.001 '**' 0.01 '*' 0.05 '.' 0.1 ' ' 1

(Dispersion parameter for binomial family taken to be 1)

    Null deviance: 5089.2  on 4242  degrees of freedom
Residual deviance: 4871.9  on 4239  degrees of freedom
AIC: 4879.9

Number of Fisher Scoring iterations: 4
```

```r
# Pairwise comparison
pairewise.h.aeg = glht(hatch.aegypti_stat,
                       mcp(treatment="Tukey"))

h.aeg<-summary(pairewise.h.aeg)

h.aeg
```

```
     Simultaneous Tests for General Linear Hypotheses

Multiple Comparisons of Means: Tukey Contrasts


Fit: glm(formula = "result ~ treatment", family = binomial, data = hatch.aegypti)

Linear Hypotheses:
                        Estimate Std. Error z value Pr(>|z|)
Aaeg-M - Bra (WT) == 0  -0.16639    0.09904  -1.680    0.334
Aaeg-m - Bra (WT) == 0   0.98677    0.10649   9.266   <1e-04 ***
Aaeg-CS - Bra (WT) == 0  0.83507    0.10166   8.214   <1e-04 ***
Aaeg-m - Aaeg-M == 0     1.15316    0.09763  11.812   <1e-04 ***
Aaeg-CS - Aaeg-M == 0    1.00145    0.09233  10.846   <1e-04 ***
Aaeg-CS - Aaeg-m == 0   -0.15170    0.10029  -1.513    0.429
---
Signif. codes:  0 '***' 0.001 '**' 0.01 '*' 0.05 '.' 0.1 ' ' 1
(Adjusted p values reported -- single-step method)
```

Replication

```
# A tibble: 4 x 3
  Treatment     N     n
  <fct>     <int> <int>
1 Bra (WT)      3   727
2 Aaeg-M        3  1023
3 Aaeg-m        3  1178
4 Aaeg-CS       3  1315
```

Model fit quality

```r
check_model(hatch.aegypti_stat)
```

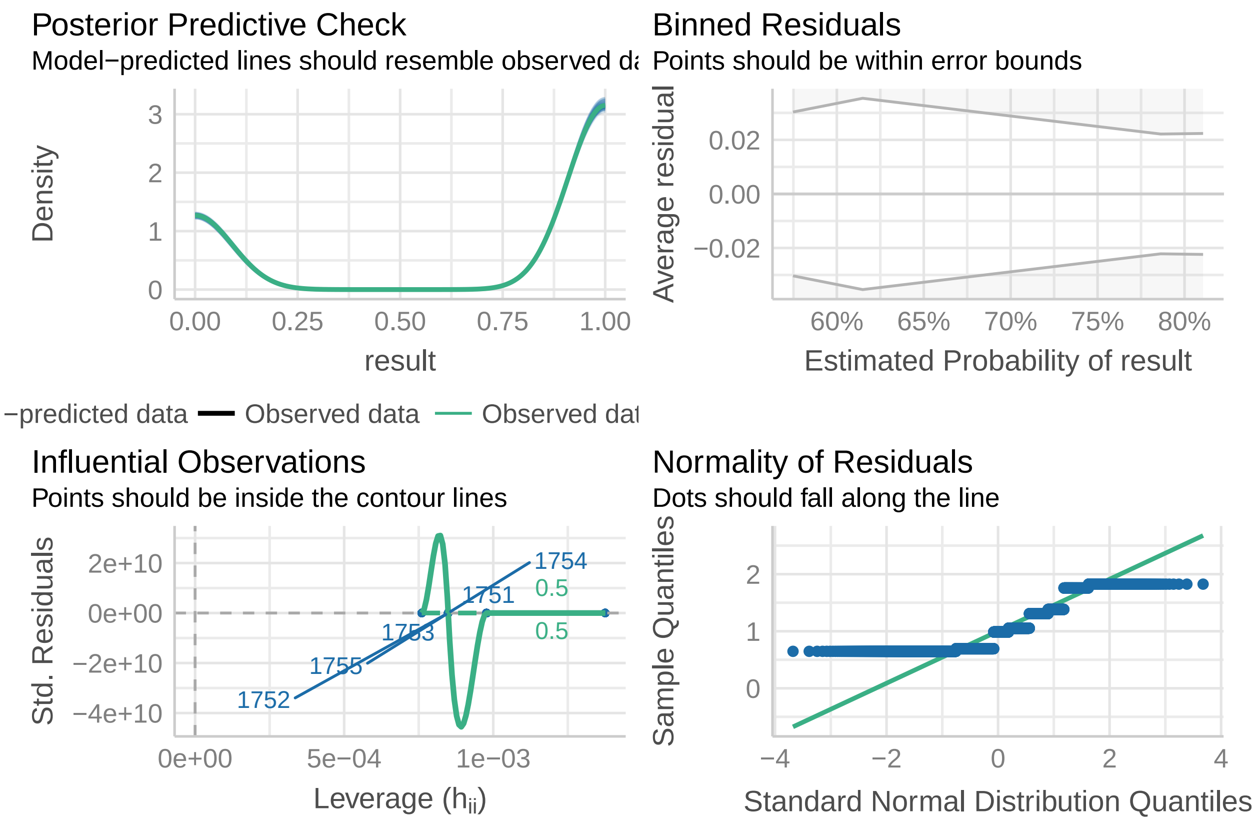

Plot .

```
# Extraction of the results
pred.aeg = data.frame(ggpredict(model = hatch.aegypti_stat,
                                terms = c("treatment")))
pred.aeg$x = factor(pred.aeg$x,
                    levels = c("Bra (WT)",
                               "Aaeg-M",
                               "Aaeg-m",
                               "Aaeg-CS"))

pred.aeg$Species = 'Ae. aegypti'


# plot
hr.plot.aeg =
  ggplot(data =  NULL) +
  geom_point(data = hr.aeg,
             aes(x = jitter(as.numeric(Line),
                            factor = 1),
                 y = hr,
                 color = Line),
             size = 2, alpha = .6) +
  geom_point(data = pred.aeg,
             aes(x = as.numeric(x),
```

```
                  y = predicted * 100),
              size = 1.5 ,
              color = 'black') +
  geom_errorbar(data = pred.aeg,
                aes(x = as.numeric(x),
                    ymin = conf.low* 100,
                    ymax = conf.high* 100),
                width = 0.05,
                color = 'black') +
  annotate(geom = 'text',
           x = summary.hatch.aeg$pos, y = 15,
           label = 'N = 3',
           size = 4) +
  annotate(geom = 'text',
           x = summary.hatch.aeg$pos, y = 7,
           label = paste0('n = ', summary.hatch.aeg$value),
           size = 3.5) +
  annotate(geom = 'line', x = 1:4, y = 100) +
  annotate(geom = 'text', x = 2.5, y = 105,
           label = '***',
           size = 4) +
  annotate(geom = 'line', x = 1:3, y = 90) +
  annotate(geom = 'text', x = 2, y = 95,
           label = '***',
           size = 4) +
  annotate(geom = 'line', x = 1:2, y = 80) +
  annotate(geom = 'text', x = 1.5, y = 85,
           label = 'n.s.',
           size = 4) +
  scale_x_continuous(breaks = 1:4,
                     labels = c("Bra (WT)",
                                "Aaeg-M",
                                "Aaeg-m",
                                "Aaeg-CS"),
                     limits = c(.5,4.5),
                     guide = guide_axis(angle = 30)) +
  scale_y_continuous(breaks = c(0, 25, 50, 75, 100),
                     limits = c(0, 100)) +
  scale_color_manual(values = line.color.aeg,
                     labels = names(line.color.aeg)) +
  labs(x = 'Line',
       y = 'Hatched eggs (%)') +
  theme_classic() +
  theme(legend.position = 'none',
        axis.title.y = element_text(color = 'black',
                                    size = 12),
        axis.text.y = element_text(size = 10,
                                   hjust = 0, vjust = 0),
        axis.title.x = element_text(color = 'black',
                                    size = 12),
        axis.text.x = element_text(size = 10))

hr.plot.aeg
```

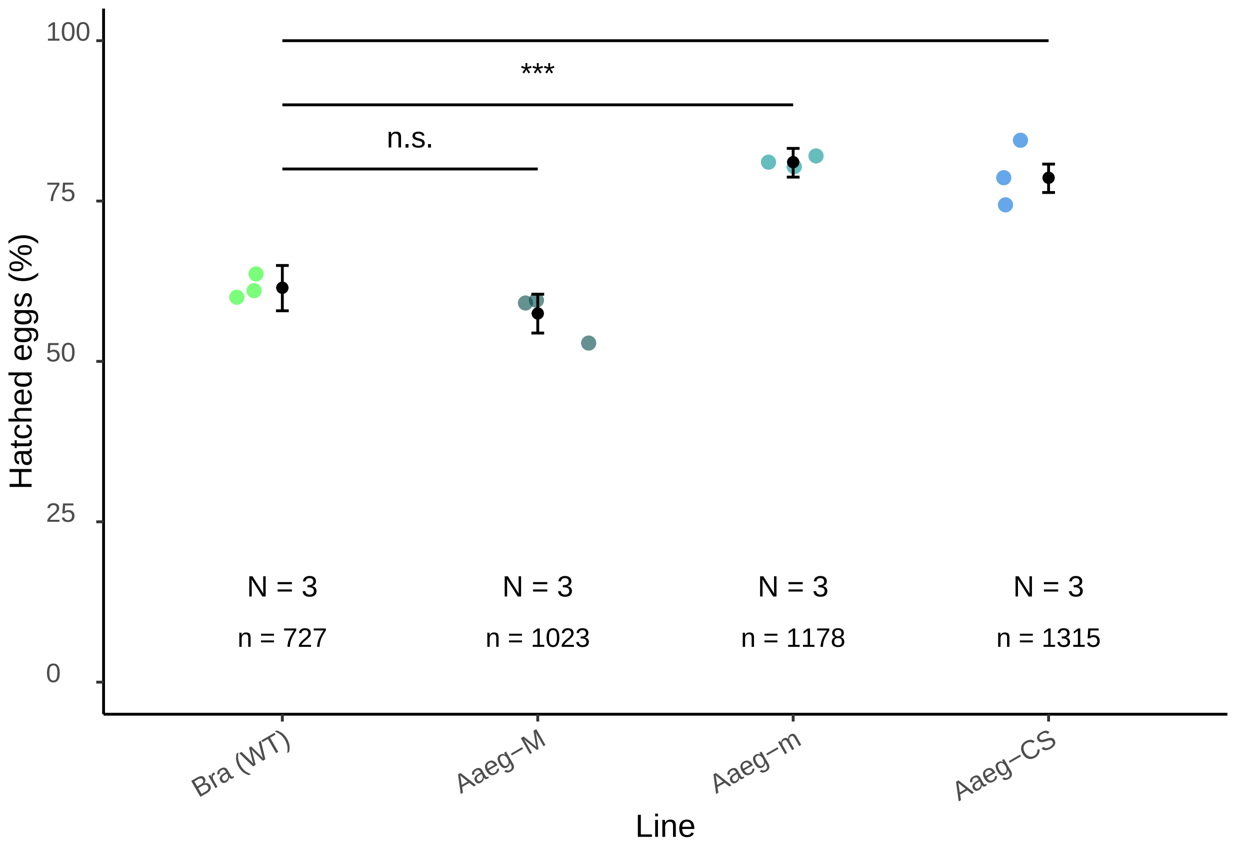

100
75
50
25
0
Hatched eggs (%)
***
n.s.
N = 3
n = 727
N = 3
n = 1023
N = 3
n = 1178
N = 3
n = 1315
Bra (WT)
Aaeg–M
Aaeg–m
Aaeg–CS
Line

Ae. albopictus

Data .

```
# Data measurements
hr.alb = read.table("data/H-rate_albo.csv",
                    header = T, sep = ";")
hr.alb$hr = hr.alb$Larvae/hr.alb$Eggs * 100
hr.alb$Line = factor(hr.alb$Line,
                     levels = c("BiA (WT)",
                                "Aal-M",
                                "Aal-m",
                                "Aal-CS"))

hr.alb
```

```
   Replicate     Line Eggs Larvae Unhatched       hr
1          1 BiA (WT)  965    555       410 57.51295
2          2 BiA (WT)  750    461       289 61.46667
3          3 BiA (WT) 1025    539       486 52.58537
4          4 BiA (WT)  730    326       404 44.65753
5          5 BiA (WT)  756    347       409 45.89947
6          1    Aal-M  927    632       295 68.17691
7          2    Aal-M  887    453       434 51.07103
8          3    Aal-M  863    490       373 56.77868
9          4    Aal-M 1473    783       690 53.15682
10         5    Aal-M  534    285       249 53.37079
11         1    Aal-m  221    103       118 46.60633
12         2    Aal-m  338    147       191 43.49112
13         3    Aal-m  587    365       222 62.18058
14         4    Aal-m  331    156       175 47.12991
15         5    Aal-m  225    114       111 50.66667
16         1   Aal-CS 1594    897       697 56.27353
17         2   Aal-CS 1633    849       784 51.99020
18         3   Aal-CS 2020   1325       695 65.59406
19         4   Aal-CS 1663    873       790 52.49549
20         5   Aal-CS 1250    557       693 44.56000
```

```
# Build binomial dataset
hatch.albo =
  rbind(
    data.frame(replicate = hr.alb[1,1],
               treatment = hr.alb[1,2],
               result = rep(0, hr.alb[1,5])),
    data.frame(replicate = hr.alb[1,1],
               treatment = hr.alb[1,2],
               result = rep(1, hr.alb[1,4])))

for(i in 2:nrow(hr.alb)){
  hatch.albo =
    rbind(
      hatch.albo,
      data.frame(replicate = hr.alb[i,1],
                 treatment = hr.alb[i,2],
```

```
                  result = rep(0, hr.alb[i,5])),
      data.frame(replicate = hr.alb[i,1],
                  treatment = hr.alb[i,2],
                  result = rep(1, hr.alb[i,4]))
    )

}
hatch.albo$treatment = factor(hatch.albo$treatment,
                              levels = c("BiA (WT)", "Aal-M", "Aal-m", "Aal-CS"))
hatch.albo$Species = 'Ae. albopictus'

summary.hatch.albo = hatch.albo %>%
  group_by(treatment) %>%
  dplyr::summarise(value = n()) %>%
  data.frame() %>%
  mutate(pos = c(1, 2, 3, 4))

head(hatch.albo)
```

```
  replicate treatment result        Species
1         1  BiA (WT)      0 Ae. albopictus
2         1  BiA (WT)      0 Ae. albopictus
3         1  BiA (WT)      0 Ae. albopictus
4         1  BiA (WT)      0 Ae. albopictus
5         1  BiA (WT)      0 Ae. albopictus
6         1  BiA (WT)      0 Ae. albopictus
```

Model .

```
# Generalized linear model with Bernoulli distribution
hatch.albo_stat = glm(formula = 'result ~ treatment',
                      family = binomial,
                      data = hatch.albo)
summary(hatch.albo_stat)
```

```
Call:
glm(formula = "result ~ treatment", family = binomial, data = hatch.albo)

Deviance Residuals:
   Min      1Q  Median      3Q     Max
-1.289  -1.267   1.070   1.091   1.144

Coefficients:
                Estimate Std. Error z value Pr(>|z|)
(Intercept)      0.10896    0.03081   3.536 0.000406 ***
treatmentAal-M   0.14952    0.04263   3.507 0.000453 ***
treatmentAal-m  -0.02901    0.05747  -0.505 0.613746
treatmentAal-CS  0.09815    0.03801   2.582 0.009816 **
---
Signif. codes:  0 '***' 0.001 '**' 0.01 '*' 0.05 '.' 0.1 ' ' 1
```

```
(Dispersion parameter for binomial family taken to be 1)

    Null deviance: 25862  on 18771  degrees of freedom
Residual deviance: 25844  on 18768  degrees of freedom
AIC: 25852

Number of Fisher Scoring iterations: 3
```

```
# Pairwise comparison
pairewise.h.alb = glht(hatch.albo_stat,
                       mcp(treatment="Tukey"))

h.albo <- summary(pairewise.h.alb)

h.albo
```

```
     Simultaneous Tests for General Linear Hypotheses

Multiple Comparisons of Means: Tukey Contrasts


Fit: glm(formula = "result ~ treatment", family = binomial, data = hatch.albo)

Linear Hypotheses:
                       Estimate Std. Error z value Pr(>|z|)
Aal-M - BiA (WT) == 0   0.14952    0.04263   3.507  0.00232 **
Aal-m - BiA (WT) == 0  -0.02901    0.05747  -0.505  0.95667
Aal-CS - BiA (WT) == 0  0.09815    0.03801   2.582  0.04630 *
Aal-m - Aal-M == 0     -0.17853    0.05676  -3.145  0.00834 **
Aal-CS - Aal-M == 0    -0.05137    0.03693  -1.391  0.49667
Aal-CS - Aal-m == 0     0.12716    0.05338   2.382  0.07725 .
---
Signif. codes:  0 '***' 0.001 '**' 0.01 '*' 0.05 '.' 0.1 ' ' 1
(Adjusted p values reported -- single-step method)
```

Replication

```
# A tibble: 4 x 3
  Treatment     N     n
  <fct>     <int> <int>
1 BiA (WT)      5  4226
2 Aal-M         5  4684
3 Aal-m         5  1702
4 Aal-CS        5  8160
```

Model fit quality

```
check_model(hatch.albo_stat)
```

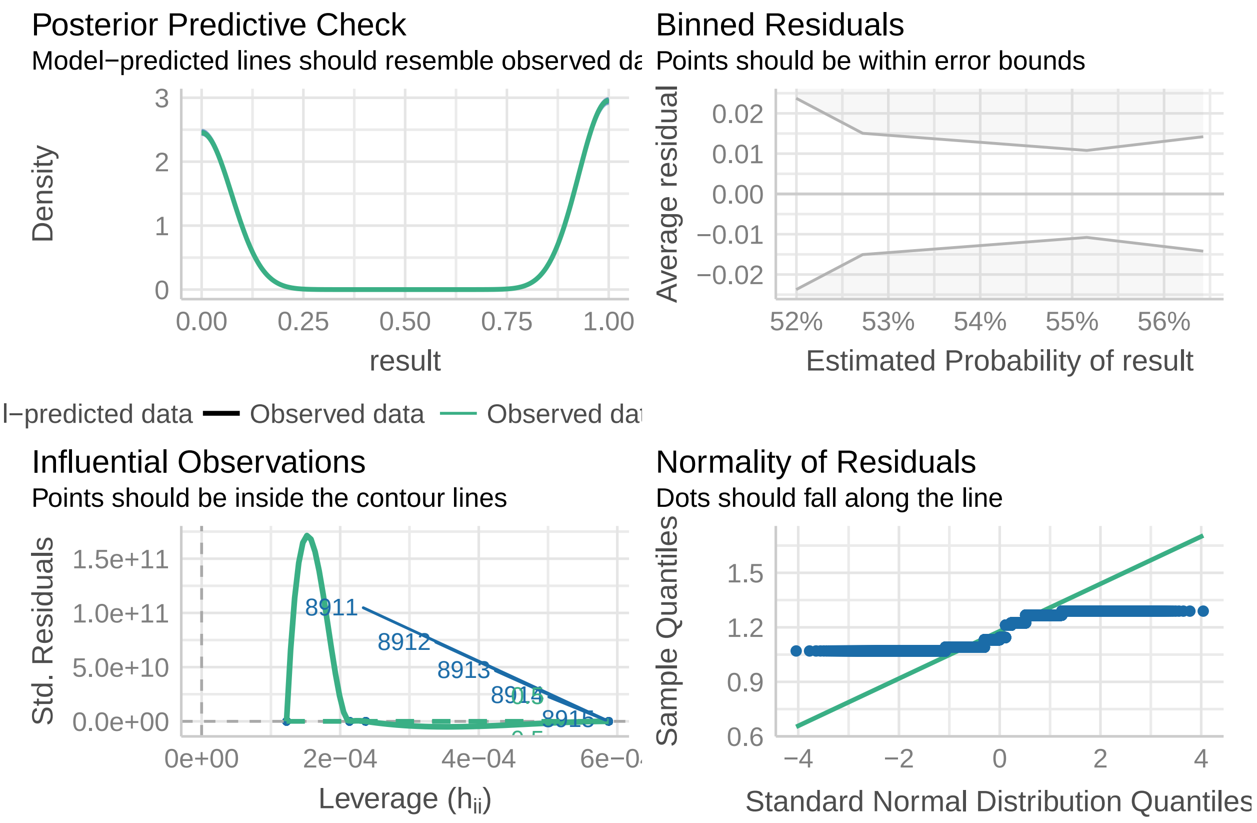

Plot .

```
# Extraction of the results
pred.alb = data.frame(ggpredict(model = hatch.albo_stat,
                                terms = c("treatment")))
pred.alb$x = as.character(pred.alb$x)
pred.alb$x = factor(pred.alb$x,
                    levels = c("BiA (WT)",
                               "Aal-M",
                               "Aal-m",
                               "Aal-CS"))
pred.alb$Species = 'Ae. albopictus'
pred.alb$group = as.factor(2)

# plot
hr.plot.alb =
  ggplot(data =  NULL) +
  geom_point(data = hr.alb,
             aes(x = jitter(as.numeric(Line),
                            factor = 1),
                 y = hr, color = Line),
             size = 2, alpha = .6) +
  geom_point(data = pred.alb,
             aes(x = as.numeric(x),
                 y = predicted * 100),
```

```
             size = 1.5 , color = 'black') +
  geom_errorbar(data = pred.alb,
                aes(x = as.numeric(x),
                    ymin = conf.low* 100,
                    ymax = conf.high* 100),
                width = 0.05,
                color = 'black') +
  annotate(geom = 'text',
           x = summary.hatch.albo$pos, y = 15,
           label = 'N = 5',
           size = 4) +
  annotate(geom = 'text',
           x = summary.hatch.albo$pos, y = 7,
           label = paste0('n = ', summary.hatch.albo$value),
           size = 3.5) +
  annotate(geom = 'line', x = 1:4, y = 100) +
  annotate(geom = 'text', x = 2.5, y = 105,
           label = '*',
           size = 4) +
  annotate(geom = 'line', x = 1:3, y = 90) +
  annotate(geom = 'text', x = 2, y = 95,
           label = 'n.s.',
           size = 4) +
  annotate(geom = 'line', x = 1:2, y = 80) +
  annotate(geom = 'text', x = 1.5, y = 85,
           label = '**',
           size = 4) +
  scale_x_continuous(breaks = 1:4,
                     labels = c("BiA (WT)",
                                "Aal-M",
                                "Aal-m",
                                "Aal-CS"),
                     limits = c(.5,4.5), guide = guide_axis(angle = 30)) +
  scale_y_continuous(breaks = c(0, 25, 50, 75, 100), limits = c(0, 100)) +
  scale_color_manual(values = line.color.alb,
                     labels = names(line.color.alb)) +
  labs(x = 'Line',
       y = 'Hatched eggs (%)') +
  theme_classic() +
  theme(panel.background =
          element_rect(fill = species.bg[names(species.bg)=="Ae. albopictus"]),
        legend.position = 'none',
        axis.title.y = element_text(color = 'black',
                                    size = 12),
        axis.text.y = element_text(size = 10,
                                   hjust = 0, vjust = 0),
        axis.title.x = element_text(color = 'black',
                                    size = 12),
        axis.text.x = element_text(size = 10))

hr.plot.alb
```

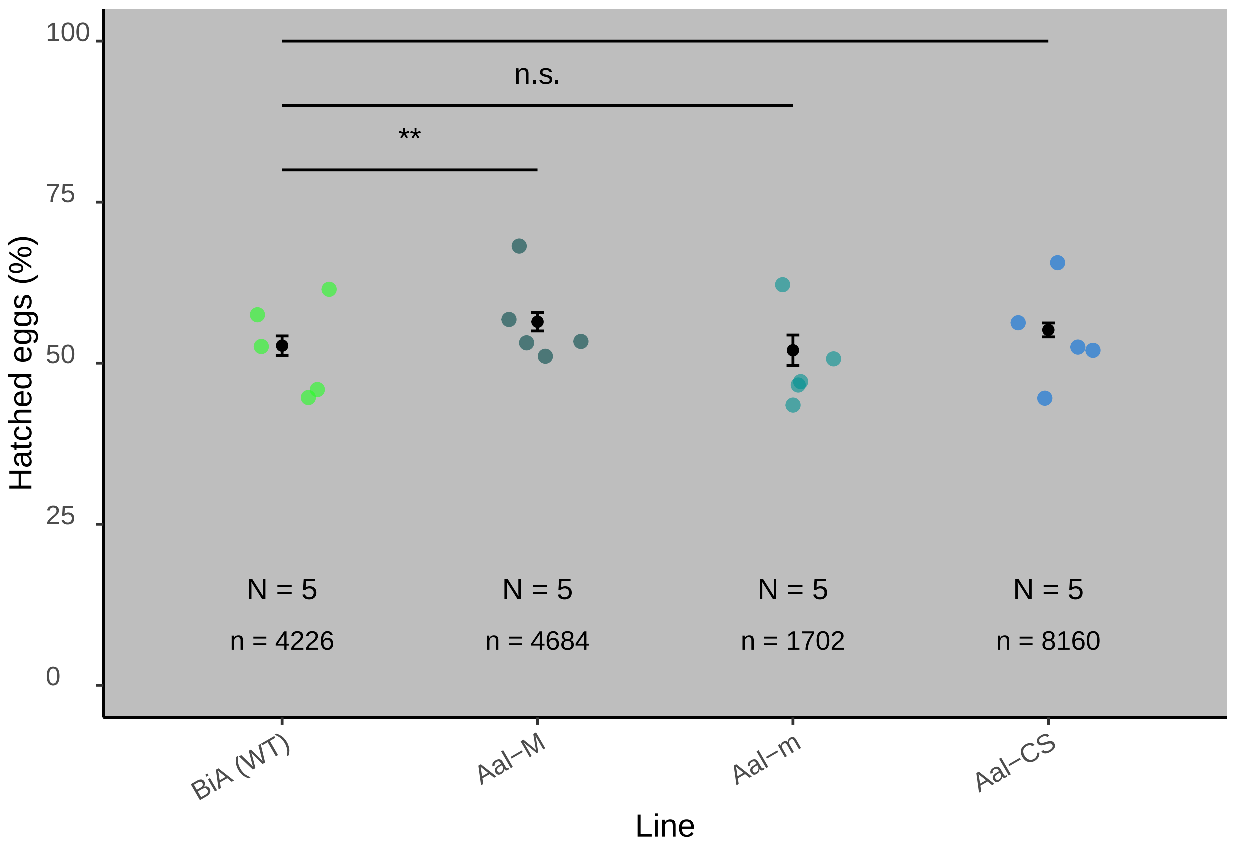

100
75
50
25
0
Hatched eggs (%)
n.s.
**
N = 5
n = 4226
N = 5
n = 4684
N = 5
n = 1702
N = 5
n = 8160
BiA (WT)
Aal–M
Aal–m
Aal–CS
Line

Fecundity

Ae. aegypti

Data .

```
fecund_aeg = read.table("data/Fecundity_aeg.csv",
                        header=TRUE, sep=";")

fecund_aeg = fecund_aeg %>%
  filter(Eggs != "NA")

fecund_aeg$Line = factor(fecund_aeg$Line,
                         levels = c('Bra (WT)', 'Aaeg-M', 'Aaeg-m', 'Aaeg-CS'))

fecund_aeg$Species = "Ae. aegypti"

fecund_aeg
```

```
       Line Well Eggs     Species
1  Bra (WT)   A1   33 Ae. aegypti
2  Bra (WT)   A2    0 Ae. aegypti
3  Bra (WT)   A3    0 Ae. aegypti
4  Bra (WT)   A4   53 Ae. aegypti
5  Bra (WT)   A5   96 Ae. aegypti
6  Bra (WT)   A6    7 Ae. aegypti
7  Bra (WT)   B1    7 Ae. aegypti
8  Bra (WT)   B2    0 Ae. aegypti
9  Bra (WT)   B3   44 Ae. aegypti
10 Bra (WT)   B4   94 Ae. aegypti
11 Bra (WT)   B5   71 Ae. aegypti
12 Bra (WT)   B6   73 Ae. aegypti
13 Bra (WT)   C1    0 Ae. aegypti
14 Bra (WT)   C2    5 Ae. aegypti
15 Bra (WT)   C3    0 Ae. aegypti
16 Bra (WT)   C4    7 Ae. aegypti
17 Bra (WT)   C5    0 Ae. aegypti
18 Bra (WT)   C6   57 Ae. aegypti
19 Bra (WT)   D1   13 Ae. aegypti
20 Bra (WT)   D2    2 Ae. aegypti
21 Bra (WT)   D3    2 Ae. aegypti
22 Bra (WT)   D4   67 Ae. aegypti
23 Bra (WT)   D5    0 Ae. aegypti
24 Bra (WT)   D6    4 Ae. aegypti
25   Aaeg-M   A1    9 Ae. aegypti
26   Aaeg-M   A2    0 Ae. aegypti
27   Aaeg-M   A3   50 Ae. aegypti
28   Aaeg-M   A4    0 Ae. aegypti
29   Aaeg-M   A5   34 Ae. aegypti
30   Aaeg-M   A6    0 Ae. aegypti
31   Aaeg-M   B1   58 Ae. aegypti
32   Aaeg-M   B2   57 Ae. aegypti
33   Aaeg-M   B3   16 Ae. aegypti
34   Aaeg-M   B4    0 Ae. aegypti
```

35	Aaeg-M	B5	66	Ae. aegypti
36	Aaeg-M	B6	9	Ae. aegypti
37	Aaeg-M	C1	50	Ae. aegypti
38	Aaeg-M	C2	2	Ae. aegypti
39	Aaeg-M	C3	69	Ae. aegypti
40	Aaeg-M	C4	20	Ae. aegypti
41	Aaeg-M	C5	0	Ae. aegypti
42	Aaeg-M	C6	8	Ae. aegypti
43	Aaeg-M	D1	77	Ae. aegypti
44	Aaeg-M	D2	2	Ae. aegypti
45	Aaeg-M	D3	5	Ae. aegypti
46	Aaeg-M	D4	3	Ae. aegypti
47	Aaeg-M	D5	17	Ae. aegypti
48	Aaeg-M	D6	0	Ae. aegypti
49	Aaeg-CS	A1	71	Ae. aegypti
50	Aaeg-CS	A2	1	Ae. aegypti
51	Aaeg-CS	A3	62	Ae. aegypti
52	Aaeg-CS	A4	87	Ae. aegypti
53	Aaeg-CS	A5	83	Ae. aegypti
54	Aaeg-CS	A6	48	Ae. aegypti
55	Aaeg-CS	B1	62	Ae. aegypti
56	Aaeg-CS	B2	72	Ae. aegypti
57	Aaeg-CS	B3	48	Ae. aegypti
58	Aaeg-CS	B4	0	Ae. aegypti
59	Aaeg-CS	B5	129	Ae. aegypti
60	Aaeg-CS	B6	0	Ae. aegypti
61	Aaeg-CS	C1	0	Ae. aegypti
62	Aaeg-CS	C2	59	Ae. aegypti
63	Aaeg-CS	C3	57	Ae. aegypti
64	Aaeg-CS	C4	42	Ae. aegypti
65	Aaeg-CS	C5	48	Ae. aegypti
66	Aaeg-CS	C6	95	Ae. aegypti
67	Aaeg-CS	D3	0	Ae. aegypti
68	Aaeg-CS	D4	70	Ae. aegypti
69	Aaeg-CS	D5	114	Ae. aegypti
70	Aaeg-CS	D6	72	Ae. aegypti
71	Aaeg-m	A1	44	Ae. aegypti
72	Aaeg-m	A2	48	Ae. aegypti
73	Aaeg-m	A3	73	Ae. aegypti
74	Aaeg-m	A4	15	Ae. aegypti
75	Aaeg-m	A5	1	Ae. aegypti
76	Aaeg-m	A6	0	Ae. aegypti
77	Aaeg-m	B1	0	Ae. aegypti
78	Aaeg-m	B2	52	Ae. aegypti
79	Aaeg-m	B3	69	Ae. aegypti
80	Aaeg-m	B4	35	Ae. aegypti
81	Aaeg-m	B5	3	Ae. aegypti
82	Aaeg-m	B6	6	Ae. aegypti
83	Aaeg-m	C1	87	Ae. aegypti
84	Aaeg-m	C2	11	Ae. aegypti
85	Aaeg-m	C3	57	Ae. aegypti
86	Aaeg-m	C4	55	Ae. aegypti
87	Aaeg-m	C5	32	Ae. aegypti
88	Aaeg-m	C6	4	Ae. aegypti

```
89   Aaeg-m   D1    0 Ae. aegypti
90   Aaeg-m   D2    1 Ae. aegypti
```

```
fecund.summary.aeg = fecund_aeg %>%
  group_by(Line) %>%
  dplyr::summarise(value = n()) %>%
  data.frame() %>%
  mutate(pos = c(1, 2, 3, 4))
```

Model . 4 models were tested and compared for analyzing fecundity data: a Generalized Linear Model with Poisson distribution (mod0), a Negative Binomial Generalized Linear Model (mod), a Hurdle model with negative binomial distribution (mod1) and a Hurdle model with Poisson distribution (mod2). These models were compared using the "compare_performance" function of package "performance". From this comparison of model fits, the Hurdle model with negative binomial distribution (mod1) was chosen.

```
# Generalized Linear Model with Poisson distribution (mod0)
mod0 = glm(formula = 'Eggs ~ Line',
                     data = fecund_aeg, family = 'poisson')

check_distribution(mod0)
```

```
# Distribution of Model Family

Predicted Distribution of Residuals

 Distribution Probability
       normal         34%
      tweedie         28%
       cauchy         16%

Predicted Distribution of Response

               Distribution Probability
 neg. binomial (zero-infl.)         94%
              beta-binomial          3%
                    tweedie          3%
```

```
check_homogeneity(mod0)
```

```
OK: There is not clear evidence for different variances across groups (Bartlett Test, p = 0.433).
```

```
check_zeroinflation(mod0)
```

```
# Check for zero-inflation

   Observed zeros: 20
  Predicted zeros: 0
            Ratio: 0.00
```

```r
check_singularity(mod0)
```

```
[1] FALSE
```

```r
# Negative Binomial Generalized Linear Model (mod)
mod = MASS::glm.nb(formula = 'Eggs ~ Line',
                    data = fecund_aeg)

# Hurdle model with negative binomial distribution (mod1)
mod1 = pscl::hurdle(Eggs ~ Line,
             data = fecund_aeg,
             dist = 'negbin')

# Hurdle model with negative binomial distribution (mod1)
mod2 = pscl::hurdle(Eggs ~ Line,
             data = fecund_aeg,
             dist = 'poisson')

# Performance comparison
performance::compare_performance(mod0, mod, mod1, mod2)
```

```
# Comparison of Model Performance Indices

Name |  Model |  AIC (weights) | AICc (weights) |  BIC (weights) |   RMSE |  Sigma | Score_log | Score_s
------------------------------------------------------------------------------------------------------------
mod0 |    glm | 3463.7 (<.001) | 3464.2 (<.001) | 3473.7 (<.001) | 31.040 |  6.007 |   -19.198 |
mod  | negbin |  776.5 (0.020) |  777.2 (0.043) |  789.0 (0.756) | 31.040 |  1.109 |    -4.788 |
mod1 | hurdle |  768.8 (0.980) |  771.0 (0.957) |  791.3 (0.244) | 31.040 | 32.719 |    -5.145 |
mod2 | hurdle | 2127.5 (<.001) | 2129.3 (<.001) | 2147.5 (<.001) | 31.040 | 32.519 |   -13.424 |
```

```r
summary(mod1)
```

```
Call:
pscl::hurdle(formula = Eggs ~ Line, data = fecund_aeg, dist = "negbin")

Pearson residuals:
    Min      1Q  Median      3Q     Max 
-0.8767 -0.7530 -0.2053  0.5867  1.9791 

Count model coefficients (truncated negbin with log link):
            Estimate Std. Error z value Pr(>|z|)    
(Intercept)  3.59467    0.24406  14.728   <2e-16 ***
LineAaeg-M  -0.20305    0.34025  -0.597   0.5507    
LineAaeg-m  -0.07031    0.34473  -0.204   0.8384    
LineAaeg-CS  0.60761    0.33873   1.794   0.0728 .  
Log(theta)   0.01902    0.20161   0.094   0.9248    
Zero hurdle model coefficients (binomial with logit link):
            Estimate Std. Error z value Pr(>|z|)  
(Intercept)   0.8873     0.4491   1.976   0.0482 *
LineAaeg-M    0.2113     0.6511   0.325   0.7455  
```

```
LineAaeg-m    0.8473     0.7706   1.100   0.2715
LineAaeg-CS   0.6168     0.7122   0.866   0.3865
---
Signif. codes:  0 '***' 0.001 '**' 0.01 '*' 0.05 '.' 0.1 ' ' 1

Theta: count = 1.0192
Number of iterations in BFGS optimization: 11
Log-likelihood: -375.4 on 9 Df
```

```
emmeans::emmeans(mod1, specs = 'Line', mode = 'response')
```

```
 Line     emmean    SE df lower.CL upper.CL
 Bra (WT)   26.5  7.18 81     12.2     40.7
 Aaeg-M     23.0  5.96 81     11.2     34.8
 Aaeg-m     29.6  7.57 81     14.6     44.7
 Aaeg-CS    55.5 14.03 81     27.5     83.4

Confidence level used: 0.95
```

```
# Pairwise comparison
pred.fecund.aeg =
  emmeans::emmeans(mod1, specs = 'Line', mode = 'response') %>%
  data.frame() %>%
  dplyr::select(
    Line, mean.pro = emmean,
    sd.pro = SE,
    conf.inf = lower.CL,
    conf.sup = upper.CL
  )

head(pred.fecund.aeg)
```

```
      Line mean.pro    sd.pro conf.inf conf.sup
1 Bra (WT) 26.45834  7.182045 12.16832 40.74835
2   Aaeg-M 23.00000  5.955554 11.15031 34.84968
3   Aaeg-m 29.64999  7.574826 14.57847 44.72152
4  Aaeg-CS 55.45453 14.027949 27.54332 83.36575
```

Replication

```
      Line  N
1 Bra (WT) 24
2   Aaeg-M 24
3   Aaeg-m 20
4  Aaeg-CS 22
```

Plot .

```
fecund.plot.aeg  = ggplot(data=NULL) +
  geom_point(data = fecund_aeg,
             aes(x = jitter(as.numeric(Line)),
```

```
                 y = Eggs, color = Line),
             size = 2, show.legend = FALSE, alpha = 0.6) +
  geom_point(data = pred.fecund.aeg,
             aes(x = as.numeric(Line),
                 y = mean.pro),
             size = 1.5, show.legend = FALSE,
             color = 'black') +
  geom_errorbar(data = pred.fecund.aeg,
                aes(x = as.numeric(Line),
                    ymin = conf.inf,
                    ymax = conf.sup), width = 0.05,
                color = 'black') +
  annotate(geom = 'text', x= fecund.summary.aeg$pos, y = -15,
           label = paste0('N = ', fecund.summary.aeg$value),
           size = 4) +
  annotate(geom = 'line', x= 1:2, y=170) +
  annotate(geom = 'text', x= 1.5, y=185,
           label = 'n.s. (n.s.)',
           size = 4) +
  annotate(geom = 'line', x= 1:3, y=205) +
  annotate(geom = 'text', x= 2, y=220,
           label = 'n.s. (n.s.)',
           size = 4) +
  annotate(geom = 'line', x= 1:4, y=240) +
  annotate(geom = 'text', x= 2.5, y=255,
           label = '. (n.s.)',
           size = 4) +
  labs(x = 'Line', y = 'Number of eggs per female',
       color = "Line") +
  scale_y_continuous(breaks = c(0, 50, 100, 150), labels = c(0, 50, 100, 150)) +
  coord_cartesian(clip = 'off', ylim = c(-20,150)) +
  scale_color_manual(values = line.color.aeg,
                     labels = names(line.color.aeg)) +
  scale_x_continuous(guide = guide_axis(angle = 30), breaks = 1:4,
                     labels = c("Bra (WT)", "Aaeg-M", "Aaeg-m", "Aaeg-CS")) +
  theme_classic() +
  theme(legend.position = 'none',
        axis.title.y = element_text(color = 'black',
                                    size = 12),
        axis.text.y = element_text(size = 10,
                                   hjust = 0, vjust = 0),
        axis.title.x = element_text(color = 'black',
                                    size = 12),
        axis.text.x = element_text(size = 10),
        plot.margin = unit(c(2,0,0,0), 'cm'))
fecund.plot.aeg
```

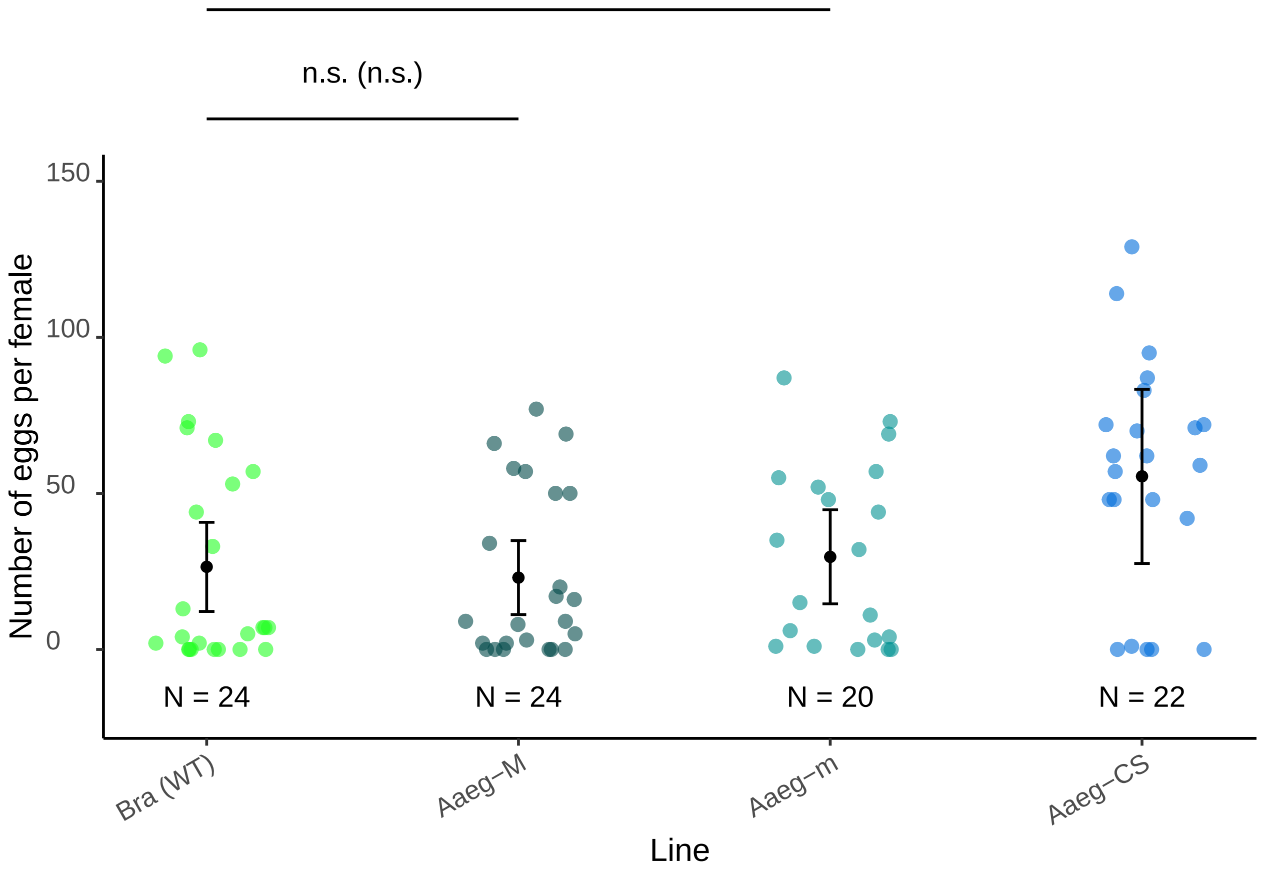
n.s. (n.s.)
150
100
50
0
Number of eggs per female
N = 24
N = 24
N = 20
N = 22
Bra (WT)
Aaeg–M
Aaeg–m
Aaeg–CS
Line

Ae. albopictus

Data .

```
fecund_albo = read.table("data/Fecundity_albo.csv", header=TRUE, sep=";")

fecund_albo = fecund_albo %>% filter(Eggs != "NA")

fecund_albo$Line = factor(fecund_albo$Line,
                          levels = c('BiA (WT)', 'Aal-M', 'Aal-m', 'Aal-CS'))

fecund_albo$Species = "Ae. albopictus"

fecund_albo
```

```
       Line Well Eggs        Species
1  BiA (WT)   A1    3 Ae. albopictus
2  BiA (WT)   A2  100 Ae. albopictus
3  BiA (WT)   A3    1 Ae. albopictus
4  BiA (WT)   A4    0 Ae. albopictus
5  BiA (WT)   A5   97 Ae. albopictus
6  BiA (WT)   A6   17 Ae. albopictus
7  BiA (WT)   B1    1 Ae. albopictus
8  BiA (WT)   B2  131 Ae. albopictus
9  BiA (WT)   B3   67 Ae. albopictus
10 BiA (WT)   B4    2 Ae. albopictus
11 BiA (WT)   B5   75 Ae. albopictus
12 BiA (WT)   B6    3 Ae. albopictus
13 BiA (WT)   C1    7 Ae. albopictus
14 BiA (WT)   C2  117 Ae. albopictus
15 BiA (WT)   C3    0 Ae. albopictus
16 BiA (WT)   C4    0 Ae. albopictus
17 BiA (WT)   C5   19 Ae. albopictus
18 BiA (WT)   C6  100 Ae. albopictus
19 BiA (WT)   D1   38 Ae. albopictus
20 BiA (WT)   D2   61 Ae. albopictus
21 BiA (WT)   D3    0 Ae. albopictus
22 BiA (WT)   D4    0 Ae. albopictus
23 BiA (WT)   D6   42 Ae. albopictus
24   Aal-CS   A1   12 Ae. albopictus
25   Aal-CS   A2    0 Ae. albopictus
26   Aal-CS   A3    0 Ae. albopictus
27   Aal-CS   A4    1 Ae. albopictus
28   Aal-CS   A5   32 Ae. albopictus
29   Aal-CS   B1   23 Ae. albopictus
30   Aal-CS   B2  133 Ae. albopictus
31   Aal-CS   B3    0 Ae. albopictus
32   Aal-CS   B4    0 Ae. albopictus
33   Aal-CS   B5  106 Ae. albopictus
34   Aal-CS   B6    0 Ae. albopictus
35   Aal-CS   C1    0 Ae. albopictus
36   Aal-CS   C2  104 Ae. albopictus
37   Aal-CS   C3   77 Ae. albopictus
38   Aal-CS   C4    7 Ae. albopictus
```

```
39   Aal-CS   C5   65 Ae. albopictus
40   Aal-CS   C6   84 Ae. albopictus
41   Aal-CS   D1    4 Ae. albopictus
42   Aal-CS   D3   27 Ae. albopictus
43   Aal-CS   D4   33 Ae. albopictus
44   Aal-CS   D5    0 Ae. albopictus
45   Aal-CS   D6   87 Ae. albopictus
46    Aal-M   A1   58 Ae. albopictus
47    Aal-M   A2   74 Ae. albopictus
48    Aal-M   A3  128 Ae. albopictus
49    Aal-M   A4   49 Ae. albopictus
50    Aal-M   A5   56 Ae. albopictus
51    Aal-M   A6   64 Ae. albopictus
52    Aal-M   B1  142 Ae. albopictus
53    Aal-M   B2    0 Ae. albopictus
54    Aal-M   B3    0 Ae. albopictus
55    Aal-M   B4  127 Ae. albopictus
56    Aal-M   B5    1 Ae. albopictus
57    Aal-M   B6  109 Ae. albopictus
58    Aal-M   C1    9 Ae. albopictus
59    Aal-M   C2   28 Ae. albopictus
60    Aal-M   C3  103 Ae. albopictus
61    Aal-M   C4  136 Ae. albopictus
62    Aal-M   C5    0 Ae. albopictus
63    Aal-M   C6  108 Ae. albopictus
64    Aal-M   D1  120 Ae. albopictus
65    Aal-m   A1    0 Ae. albopictus
66    Aal-m   A2    0 Ae. albopictus
67    Aal-m   A3    0 Ae. albopictus
68    Aal-m   A4    0 Ae. albopictus
69    Aal-m   A5    0 Ae. albopictus
70    Aal-m   A6  120 Ae. albopictus
71    Aal-m   B1   62 Ae. albopictus
72    Aal-m   B2   66 Ae. albopictus
73    Aal-m   B3   29 Ae. albopictus
74    Aal-m   B4   44 Ae. albopictus
75    Aal-m   B5    0 Ae. albopictus
76    Aal-m   B6  116 Ae. albopictus
77    Aal-m   C1    0 Ae. albopictus
78    Aal-m   C2   44 Ae. albopictus
79    Aal-m   C3   88 Ae. albopictus
80    Aal-m   C4    6 Ae. albopictus
81    Aal-m   C5   27 Ae. albopictus
82    Aal-m   C6    0 Ae. albopictus
83    Aal-m   D1  106 Ae. albopictus
84    Aal-m   D2    0 Ae. albopictus
85    Aal-m   D3  113 Ae. albopictus
86    Aal-m   D4    0 Ae. albopictus
87    Aal-m   D5    0 Ae. albopictus
88    Aal-m   D6  137 Ae. albopictus
```

```
fecund.summary.alb = fecund_albo %>%
  group_by(Line) %>%
  dplyr::summarise(value = n()) %>%
```

```
  data.frame() %>%
  mutate(pos = c(1, 2, 3, 4))
```

Model .

Similarly to Ae. aegypti, a Hurdle model with negative binomial distribution (mod1) was chosen for analizing fecundity data in Ae. albopictus.

```
# Generalized Linear Model with Poisson distribution (mod0)
mod0 = glm(formula = 'Eggs ~ Line',
                     data = fecund_albo, family = 'poisson')
check_distribution(mod0)
```

```
# Distribution of Model Family

Predicted Distribution of Residuals

 Distribution Probability
       normal         31%
      tweedie         25%
       cauchy         16%

Predicted Distribution of Response

               Distribution Probability
 neg. binomial (zero-infl.)         81%
              beta-binomial          9%
                    tweedie          6%
```

```
check_homogeneity(mod0)
```

```
OK: There is not clear evidence for different variances across groups (Bartlett Test, p = 0.859).
```

```
check_zeroinflation(mod0)
```

```
# Check for zero-inflation

   Observed zeros: 26
  Predicted zeros: 0
            Ratio: 0.00
```

```
check_singularity(mod0)
```

```
[1] FALSE
```

```
# Negative Binomial Generalized Linear Model (mod)
mod = MASS::glm.nb(formula = 'Eggs ~ Line',
                  data = fecund_albo)

# Hurdle model with negative binomial distribution (mod1)
```

```r
mod1 = pscl::hurdle(Eggs ~ Line,
             data = fecund_albo,
             dist = 'negbin')

#Hurdle model with Poisson distribution (mod2)
mod2 = pscl::hurdle(Eggs ~ Line,
             data = fecund_albo,
             dist = 'poisson')

# Performance comparison
performance::compare_performance(mod0, mod, mod1, mod2)
```

```
# Comparison of Model Performance Indices

Name |  Model |  AIC (weights) | AICc (weights) |  BIC (weights) |   RMSE |  Sigma | Score_log | Score_s
------------------------------------------------------------------------------------------------------------
mod0 |    glm | 5201.8 (<.001) | 5202.3 (<.001) | 5211.7 (<.001) | 45.966 |  7.604 |   -29.510 |
mod  | negbin | 5203.3 (<.001) | 5204.0 (<.001) | 5215.7 (<.001) | 45.966 |  7.604 |   -29.510 |
mod1 | hurdle |  754.2 (>.999) |  756.5 (>.999) |  776.5 (>.999) | 45.966 | 48.514 |    -4.925 |
mod2 | hurdle | 2644.5 (<.001) | 2646.4 (<.001) | 2664.4 (<.001) | 45.966 | 48.209 |   -18.201 |
```

```r
summary(mod1)
```

```
Call:
pscl::hurdle(formula = Eggs ~ Line, data = fecund_albo, dist = "negbin")

Pearson residuals:
    Min      1Q  Median      3Q     Max
-0.8646 -0.7144 -0.2253  0.6598  1.9719

Count model coefficients (truncated negbin with log link):
            Estimate Std. Error z value Pr(>|z|)
(Intercept)  3.87134    0.23601  16.403   <2e-16 ***
LineAal-M    0.52400    0.34283   1.528    0.126
LineAal-m    0.41586    0.36324   1.145    0.252
LineAal-CS   0.08113    0.34946   0.232    0.816
Log(theta)   0.02139    0.20175   0.106    0.916
Zero hurdle model coefficients (binomial with logit link):
            Estimate Std. Error z value Pr(>|z|)
(Intercept)   1.2809     0.5055   2.534   0.0113 *
LineAal-M     0.3930     0.8071   0.487   0.6263
LineAal-m    -1.1139     0.6507  -1.712   0.0869 .
LineAal-CS   -0.5188     0.6820  -0.761   0.4468
---
Signif. codes:  0 '***' 0.001 '**' 0.01 '*' 0.05 '.' 0.1 ' ' 1

Theta: count = 1.0216
Number of iterations in BFGS optimization: 11
Log-likelihood: -368.1 on 9 Df
```

```
emmeans::emmeans(mod1, specs = 'Line', mode = 'response')
```

```
 Line     emmean    SE df lower.CL upper.CL
 BiA (WT)   38.3  9.81 79     18.8     57.8
 Aal-M      69.1 18.33 79     32.6    105.5
 Aal-m      39.9 13.22 79     13.6     66.2
 Aal-CS     36.1 10.57 79     15.1     57.2

Confidence level used: 0.95
```

```
# Pairwise comparison
pred.fecund.albo =
  emmeans::emmeans(mod1, specs = 'Line', mode = 'response') %>%
  data.frame() %>%
  dplyr::select(
    Line, mean.pro = emmean,
    sd.pro = SE,
    conf.inf = lower.CL,
    conf.sup = upper.CL
  )

head(pred.fecund.albo)
```

```
      Line mean.pro    sd.pro conf.inf  conf.sup
1 BiA (WT) 38.30436  9.812262 18.77354  57.83518
2    Aal-M 69.05264 18.331136 32.56543 105.53986
3    Aal-m 39.91664 13.223746 13.59543  66.23785
4   Aal-CS 36.13637 10.567392 15.10250  57.17024
```

Replication

```
      Line  N
1 BiA (WT) 23
2    Aal-M 19
3    Aal-m 24
4   Aal-CS 22
```

Plot .

```
fecund.plot.alb  = ggplot(data=NULL) +
  geom_point(data = fecund_albo,
             aes(x = jitter(as.numeric(Line)),
                 y = Eggs, color = Line),
             size = 2, show.legend = FALSE, alpha = 0.6) +
  geom_point(data = pred.fecund.albo,
             aes(x = as.numeric(Line),
                 y = mean.pro),
             size = 1.5, show.legend = FALSE,
             color = 'black') +
  geom_errorbar(data = pred.fecund.albo,
                aes(x = as.numeric(Line),
```

```
                    ymin = conf.inf,
                    ymax = conf.sup), width = 0.05,
                color = 'black') +
  annotate(geom = 'text', x= fecund.summary.alb$pos, y = -15,
           label = paste0('N = ', fecund.summary.alb$value),
           size = 4) +
  annotate(geom = 'line', x= 1:2, y=170) +
  annotate(geom = 'text', x= 1.5, y=185,
           label = '*** (n.s.)',
           size = 4) +
  annotate(geom = 'line', x= 1:3, y=205) +
  annotate(geom = 'text', x= 2, y=220,
           label = '*** (.)',
           size = 4) +
  annotate(geom = 'line', x= 1:4, y=240) +
  annotate(geom = 'text', x= 2.5, y=255,
           label = 'n.s. (n.s.)',
           size = 4) +
  labs(x = 'Line', y = 'Number of eggs per female',
       color = "Line") +
  scale_y_continuous(breaks = c(0, 50, 100, 150), labels = c(0, 50, 100, 150)) +
  coord_cartesian(clip = 'off', ylim = c(-20,150)) +
  scale_color_manual(values = line.color.alb,
                     labels = names(line.color.alb)) +
  scale_x_continuous(guide = guide_axis(angle = 30), breaks = 1:4,
                     labels = c("BiA (WT)",
                                "Aal-M",
                                "Aal-m",
                                "Aal-CS")) +
  theme_classic() +
  theme(legend.position = 'none',
        panel.background = element_rect(fill = "grey"),
        axis.title.y = element_text(color = 'black',
                                    size = 12),
        axis.text.y = element_text(size = 10,
                                   hjust = 0, vjust = 0),
        axis.title.x = element_text(color = 'black',
                                    size = 12),
        axis.text.x = element_text(size = 10),
        plot.margin = unit(c(2,0,0,0), 'cm'))

fecund.plot.alb
```

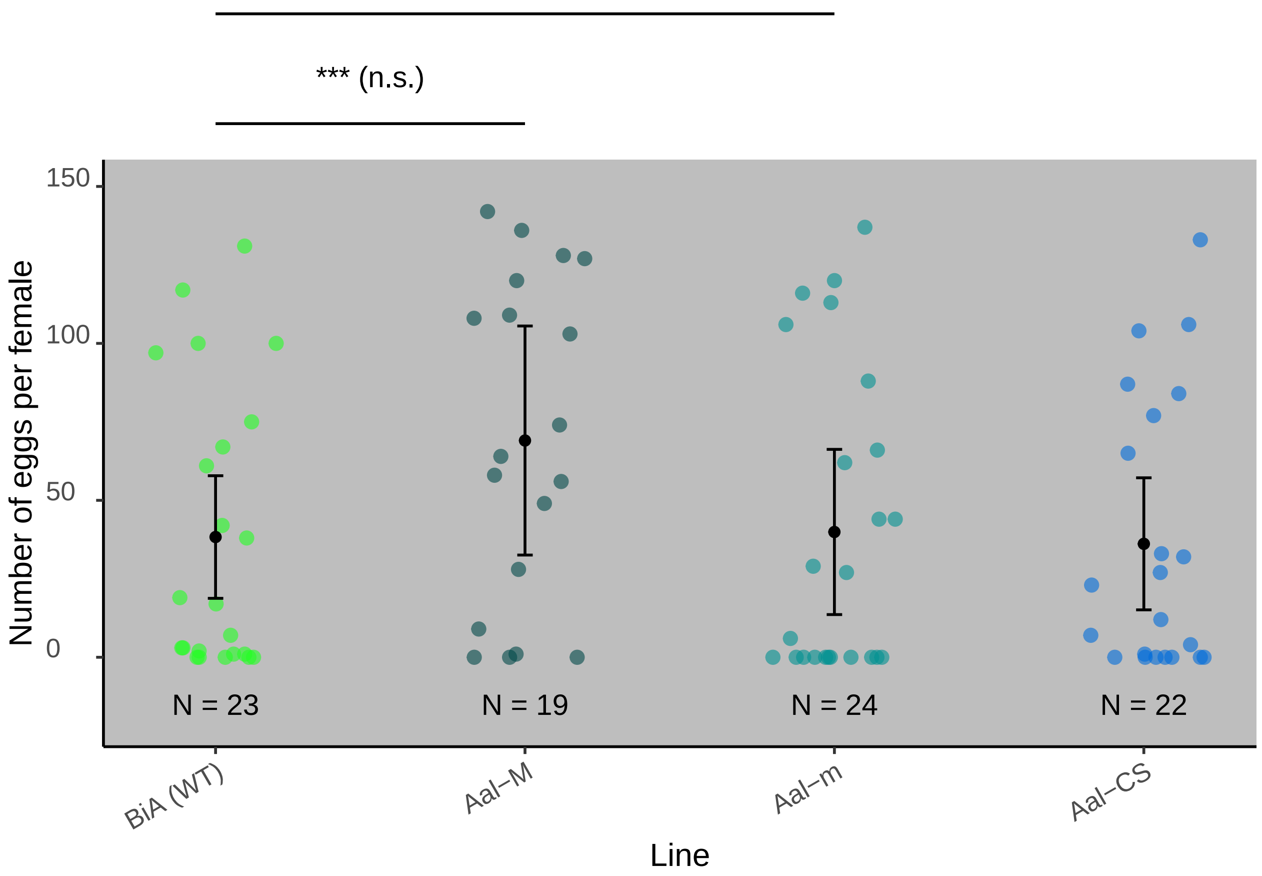
*** (n.s.)
150
100
50
0
Number of eggs per female
N = 23
N = 19
N = 24
N = 22
BiA (WT)
Aal–M
Aal–m
Aal–CS
Line

Larva survival

Ae. aegypti

Data .

```
surv.aeg.larv = read.table("data/Survival-larvae_aeg.csv",
                           sep = ";", header = T)
surv.aeg.larv = surv.aeg.larv %>%
  dplyr::select(Replicate, Line, larvae, adults)

surv.aeg.larv$Species = "Ae. aegypti"

surv.aeg.larv$Line = factor(surv.aeg.larv$Line,
                            levels = c('Bra (WT)', 'Aaeg-M', 'Aaeg-m', 'Aaeg-CS'))

surv.aeg.larv$survie = surv.aeg.larv$adults/surv.aeg.larv$larvae * 100

surv.aeg.larv
```

```
   Replicate     Line larvae adults     Species survie
1          1   Aaeg-M    100     87 Ae. aegypti   87.0
2          2   Aaeg-M    100     82 Ae. aegypti   82.0
3          3   Aaeg-M    100     93 Ae. aegypti   93.0
4          4   Aaeg-M    100     73 Ae. aegypti   73.0
5          1   Aaeg-m    100     96 Ae. aegypti   96.0
6          2   Aaeg-m    100    100 Ae. aegypti  100.0
7          3   Aaeg-m    100     96 Ae. aegypti   96.0
8          4   Aaeg-m    100     90 Ae. aegypti   90.0
9          1 Bra (WT)    200    185 Ae. aegypti   92.5
10         2 Bra (WT)    200    197 Ae. aegypti   98.5
11         3 Bra (WT)    200    177 Ae. aegypti   88.5
12         4 Bra (WT)    200    197 Ae. aegypti   98.5
13         1  Aaeg-CS    100     96 Ae. aegypti   96.0
14         2  Aaeg-CS    100     98 Ae. aegypti   98.0
15         3  Aaeg-CS    100     99 Ae. aegypti   99.0
16         4  Aaeg-CS    100    100 Ae. aegypti  100.0
17         5   Aaeg-M    100     95 Ae. aegypti   95.0
18         6   Aaeg-M    100     95 Ae. aegypti   95.0
19         7   Aaeg-M    100     95 Ae. aegypti   95.0
20         8   Aaeg-M    100     97 Ae. aegypti   97.0
```

Model .

```
mod.tot.sl.aeg = lm(data=surv.aeg.larv, formula = survie ~ Line)
mod.tot.sl.aeg %>% summary()
```

```
Call:
lm(formula = survie ~ Line, data = surv.aeg.larv)

Residuals:
```

```
    Min      1Q  Median      3Q     Max 
-16.625  -2.344   0.625   4.125   7.375 

Coefficients:
            Estimate Std. Error t value Pr(>|t|)    
(Intercept)   94.500      3.127  30.225 1.52e-15 ***
LineAaeg-M    -4.875      3.829  -1.273    0.221    
LineAaeg-m     1.000      4.422   0.226    0.824    
LineAaeg-CS    3.750      4.422   0.848    0.409    
---
Signif. codes:  0 '***' 0.001 '**' 0.01 '*' 0.05 '.' 0.1 ' ' 1

Residual standard error: 6.253 on 16 degrees of freedom
Multiple R-squared:  0.2691,    Adjusted R-squared:  0.1321 
F-statistic: 1.964 on 3 and 16 DF,  p-value: 0.1601
```

```
mod.tot.sl.aeg %>% aov %>% TukeyHSD()
```

```
  Tukey multiple comparisons of means
    95% family-wise confidence level

Fit: aov(formula = .)

$Line
                   diff        lwr       upr     p adj
Aaeg-M-Bra (WT)  -4.875 -15.830534  6.080534 0.5920067
Aaeg-m-Bra (WT)   1.000 -11.650361 13.650361 0.9957513
Aaeg-CS-Bra (WT)  3.750  -8.900361 16.400361 0.8308209
Aaeg-m-Aaeg-M     5.875  -5.080534 16.830534 0.4414047
Aaeg-CS-Aaeg-M    8.625  -2.330534 19.580534 0.1514294
Aaeg-CS-Aaeg-m    2.750  -9.900361 15.400361 0.9235384
```

```
df.larv.aeg = ggpredict(mod.tot.sl.aeg) %>%
  data.frame %>%
  dplyr::select(
    Line = Line.x,
    m = Line.predicted,
    sd = Line.std.error,
    conf.inf = Line.conf.low,
    conf.sup = Line.conf.high
  )
df.larv.aeg$Line = factor(df.larv.aeg$Line,
                          levels = c('Bra (WT)', 'Aaeg-M', 'Aaeg-m', 'Aaeg-CS'))

df.larv.aeg
```

```
      Line      m       sd conf.inf  conf.sup
1 Bra (WT) 94.500 3.126562 87.87198 101.12802
2   Aaeg-M 89.625 2.210813 84.93829  94.31171
3   Aaeg-m 95.500 3.126562 88.87198 102.12802
4  Aaeg-CS 98.250 3.126562 91.62198 104.87802
```

Replication

```
# A tibble: 4 x 3
  Line          N     n
  <fct>     <int> <int>
1 Bra (WT)      4   800
2 Aaeg-M        8   800
3 Aaeg-m        4   400
4 Aaeg-CS       4   400
```

Model quality

.

```
check_model(mod.tot.sl.aeg)
```

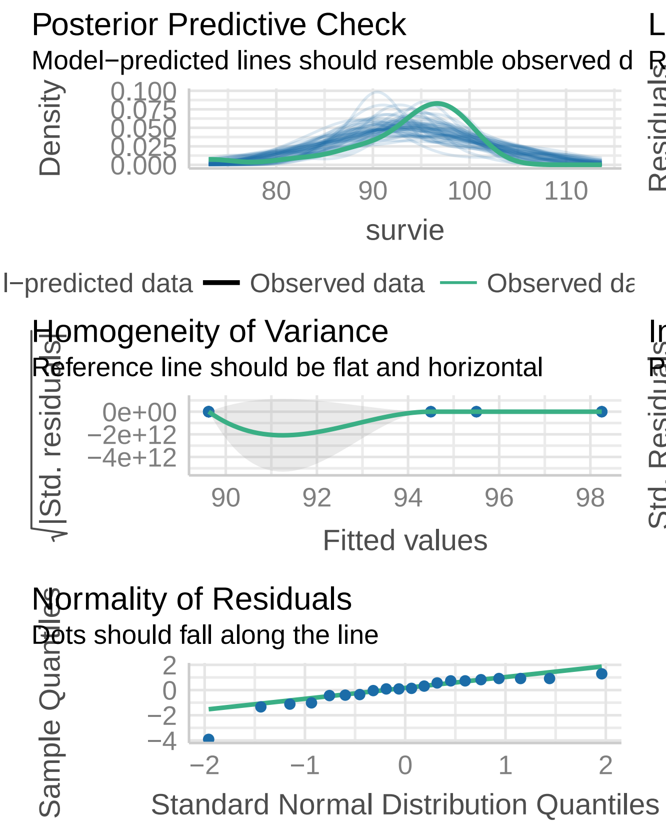


```
larval.surv.plot.aeg = ggplot(data = NULL,
                              aes()) +
  geom_point(data = surv.aeg.larv,
             aes(x = jitter(as.numeric(factor(Line))),
                 y = survie, color = Line),
             size = 2, alpha = 0.6) +
  geom_point(data = df.larv.aeg,
             aes(x = Line, y = m),
             col = 'black') +
```

```
  geom_errorbar(data = df.larv.aeg, aes(x = Line, ymin = conf.inf,
                                        ymax = conf.sup),
              width = 0.05, col = 'black') +
  annotate(geom = 'text', x = 1:4, y = 20,
           label = c("N = 4", "N = 8", "N = 4", "N = 4"),
           size = 4) +
  annotate(geom = 'text', x = 1:4, y = 5,
           label = c("n = 800", "n = 400", "n = 400", "n = 400"),
           size = 4) +
  annotate(geom = 'line', x = 1:4, y = 152) +
  annotate(geom = 'text', x = 2.5, y = 162,
           label = 'n.s.',
           size = 4) +
  annotate(geom = 'line', x = 1:3, y = 132) +
  annotate(geom = 'text', x = 2, y = 142,
           label = 'n.s.',
           size = 4) +
  annotate(geom = 'line', x = 1:2, y = 112) +
  annotate(geom = 'text', x = 1.5, y = 122,
           label = 'n.s.',
           size = 4) +
  scale_x_discrete(limits = levels(df.larv.aeg$Line),
                 labels = c("Bra (WT)",
                            "Aaeg-M",
                            "Aaeg-m",
                            "Aaeg-CS"
                 ),
  guide = guide_axis(angle = 30)) +
  scale_y_continuous(breaks = c(0, 25, 50, 75, 100),
                   labels = c(0, 25, 50, 75, 100)) +
  scale_color_manual(labels = names(line.color.aeg),
                   values = line.color.aeg) +
  coord_cartesian(clip = 'off', ylim = c(0,100)) +
  labs(x = 'Line',
       y = 'Larva to adult survival (%)',
       color = 'Line') +
  theme_classic() +
  theme(legend.position = 'none',
        axis.title.y = element_text(color = 'black',
                                    size = 12),
        axis.text.y = element_text(size = 10,
                                   hjust = 0, vjust = 0),
        axis.title.x = element_text(color = 'black',
                                    size = 12),
        axis.text.x = element_text(size = 10),
        plot.margin = unit(c(2,0,0,0), 'cm'))


larval.surv.plot.aeg
```

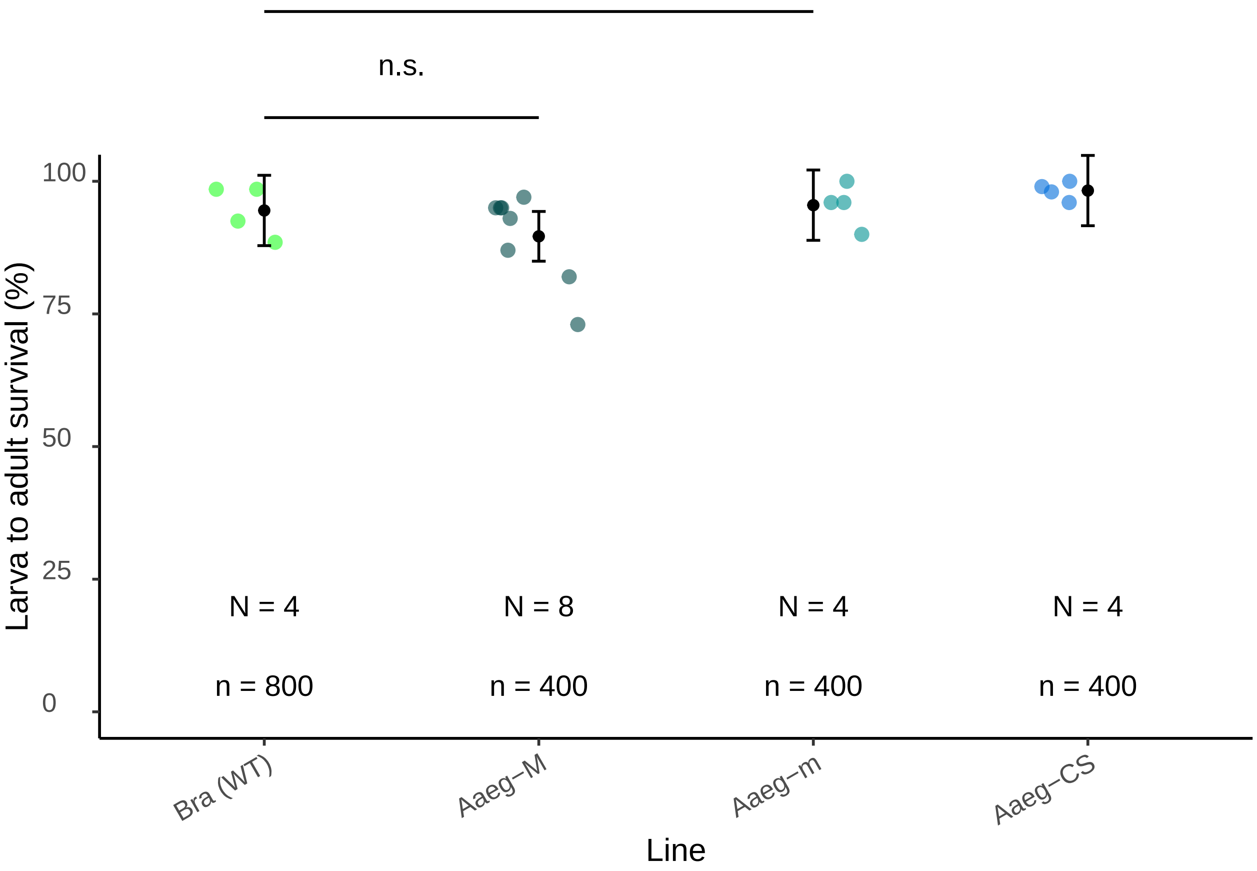

n.s.
100
75
50
25
0
Larva to adult survival (%)
N = 4
N = 8
N = 4
N = 4
n = 800
n = 400
n = 400
n = 400
Bra (WT)
Aaeg−M
Aaeg−m
Aaeg−CS
Line

Plot

Ae. albopictus

Data .

```
surv.alb.larv = read.table("data/Survival-larvae_albo.csv",
                           sep = ";", header = T)
surv.alb.larv = surv.alb.larv %>%
  dplyr::select( Replicate, Line, larvae, adults)

surv.alb.larv$Species = "Ae. albopictus"

surv.alb.larv$Line = factor(surv.alb.larv$Line,
                            levels = c('BiA (WT)', 'Aal-M', 'Aal-m', 'Aal-CS'))

surv.alb.larv$survie = surv.alb.larv$adults/surv.alb.larv$larvae * 100

surv.alb.larv
```

```
   Replicate     Line larvae adults        Species survie
1          1 BiA (WT)    200    103 Ae. albopictus   51.5
2          2 BiA (WT)    200    141 Ae. albopictus   70.5
3          3 BiA (WT)    200    129 Ae. albopictus   64.5
4          4 BiA (WT)    200    130 Ae. albopictus   65.0
5          1    Aal-M    100     88 Ae. albopictus   88.0
6          2    Aal-M    100     79 Ae. albopictus   79.0
7          3    Aal-M    100     80 Ae. albopictus   80.0
8          4    Aal-M    100     77 Ae. albopictus   77.0
9          1    Aal-m    100     82 Ae. albopictus   82.0
10         2    Aal-m    100     83 Ae. albopictus   83.0
11         3    Aal-m    100     76 Ae. albopictus   76.0
12         4    Aal-m    100     77 Ae. albopictus   77.0
13         1   Aal-CS    100     71 Ae. albopictus   71.0
14         2   Aal-CS    100     73 Ae. albopictus   73.0
15         3   Aal-CS    100     61 Ae. albopictus   61.0
16         4   Aal-CS    100     69 Ae. albopictus   69.0
```

Model .

```
mod.tot.sl.alb = lm(data=surv.alb.larv, formula = survie ~ Line)
mod.tot.sl.alb %>% summary()
```

```
Call:
lm(formula = survie ~ Line, data = surv.alb.larv)

Residuals:
    Min      1Q  Median      3Q     Max
-11.375  -2.750   1.062   2.750   7.625

Coefficients:
            Estimate Std. Error t value Pr(>|t|)
(Intercept)   62.875      2.831  22.210 4.09e-11 ***
```

```
LineAal-M     18.125      4.004   4.527 0.000693 ***
LineAal-m     16.625      4.004   4.153 0.001341 **
LineAal-CS     5.625      4.004   1.405 0.185378
---
Signif. codes:  0 '***' 0.001 '**' 0.01 '*' 0.05 '.' 0.1 ' ' 1

Residual standard error: 5.662 on 12 degrees of freedom
Multiple R-squared:  0.7043,    Adjusted R-squared:  0.6303
F-statistic: 9.525 on 3 and 12 DF,  p-value: 0.001695
```

```
mod.tot.sl.alb %>% aov %>% TukeyHSD()
```

```
  Tukey multiple comparisons of means
    95% family-wise confidence level

Fit: aov(formula = .)

$Line
                   diff        lwr        upr     p adj
Aal-M-BiA (WT)   18.125   6.238769 30.0112306 0.0033427
Aal-m-BiA (WT)   16.625   4.738769 28.5112306 0.0063439
Aal-CS-BiA (WT)   5.625  -6.261231 17.5112306 0.5198078
Aal-m-Aal-M      -1.500 -13.386231 10.3862306 0.9812185
Aal-CS-Aal-M    -12.500 -24.386231 -0.6137694 0.0383297
Aal-CS-Aal-m    -11.000 -22.886231  0.8862306 0.0730385
```

```
df.larv.alb = ggpredict(mod.tot.sl.alb) %>%
  data.frame %>%
  dplyr::select(
    Line = Line.x,
    m = Line.predicted,
    sd = Line.std.error,
    conf.inf = Line.conf.low,
    conf.sup = Line.conf.high
  )
df.larv.alb$Line = factor(df.larv.alb$Line,
                          levels = c('BiA (WT)', 'Aal-M', 'Aal-m', 'Aal-CS'))
df.larv.alb
```

```
      Line      m       sd conf.inf conf.sup
1 BiA (WT) 62.875 2.830958 56.70687 69.04313
2    Aal-M 81.000 2.830958 74.83187 87.16813
3    Aal-m 79.500 2.830958 73.33187 85.66813
4   Aal-CS 68.500 2.830958 62.33187 74.66813
```

Replication

```
# A tibble: 4 x 3
  Line         N     n
  <fct>    <int> <int>
1 BiA (WT)     4   800
2 Aal-M        4   400
3 Aal-m        4   400
4 Aal-CS       4   400
```

Model quality

```
check_model(mod.tot.sl.alb)
```

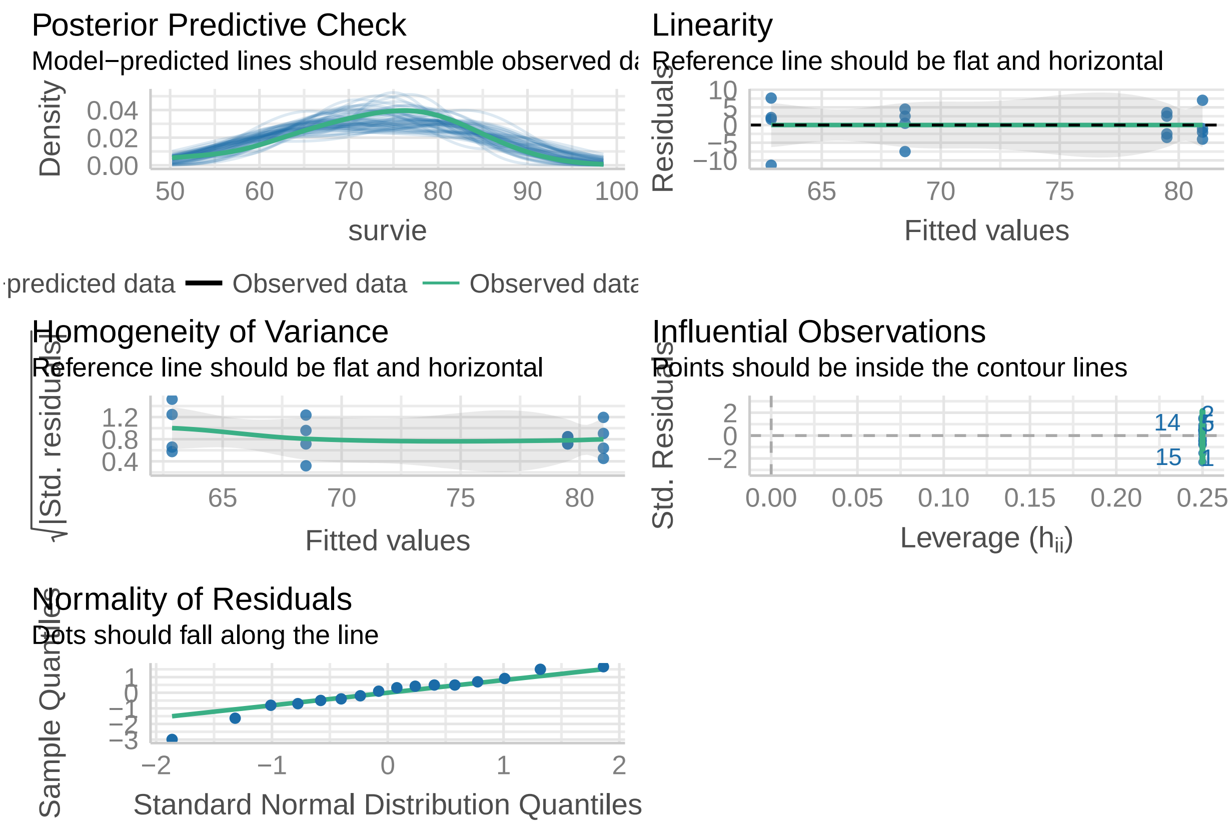

Plot .

```
larval.surv.plot.alb = ggplot(data = NULL,
                              aes()) +
  geom_point(data = surv.alb.larv,
             aes(x = jitter(as.numeric(factor(Line))),
                 y = survie, color = Line),
             size = 2, alpha = 0.6) +
  geom_point(data = df.larv.alb,
             aes(x = Line, y = m), col = 'black') +
  geom_errorbar(data = df.larv.alb,
                aes(x = Line,
                    ymin = conf.inf,
                    ymax = conf.sup),
                width = 0.05, col = 'black') +
  annotate(geom = 'text', x = 1:4, y = 20,
           label = c("N = 4", "N = 4", "N = 4", "N = 4"),
           size = 4) +
  annotate(geom = 'text', x = 1:4, y = 5,
           label = c("n = 800", "n = 400", "n = 400", "n = 400"),
           size = 4) +
```

```
  annotate(geom = 'line', x = 1:4, y = 152) +
  annotate(geom = 'text', x = 2.5, y = 162,
           label = 'n.s.',
           size = 4) +
  annotate(geom = 'line', x = 1:3, y = 132) +
  annotate(geom = 'text', x = 2, y = 142,
           label = '**',
           size = 4) +
  annotate(geom = 'line', x = 1:2, y = 112) +
  annotate(geom = 'text', x = 1.5, y = 122,
           label = '**',
           size = 4) +
  scale_x_discrete(limits = levels(df.larv.alb$Line),
                   labels = c("BiA (WT)",
                              "Aal-M",
                              "Aal-m",
                              "Aal-CS"
                   ),
  guide = guide_axis(angle = 30)) +
  scale_y_continuous(breaks = c(0, 25, 50, 75, 100),
                     labels = c(0, 25, 50, 75, 100)) +
  coord_cartesian(clip = 'off', ylim = c(0,100)) +
  scale_color_manual(labels = names(line.color.alb),
                     values = line.color.alb) +
  labs(x = 'Line',
       y = 'Larva to adult survival (%)',
       color = 'Line') +
  theme_classic() +
  theme(legend.position = 'none',
        panel.background = element_rect(fill = "grey"),
        axis.title.y = element_text(color = 'black',
                                    size = 12),
        axis.text.y = element_text(size = 10,
                                   hjust = 0, vjust = 0),
        axis.title.x = element_text(color = 'black',
                                    size = 12),
        axis.text.x = element_text(size = 10),
        plot.margin = unit(c(2,0,0,0), 'cm'))
larval.surv.plot.alb
```

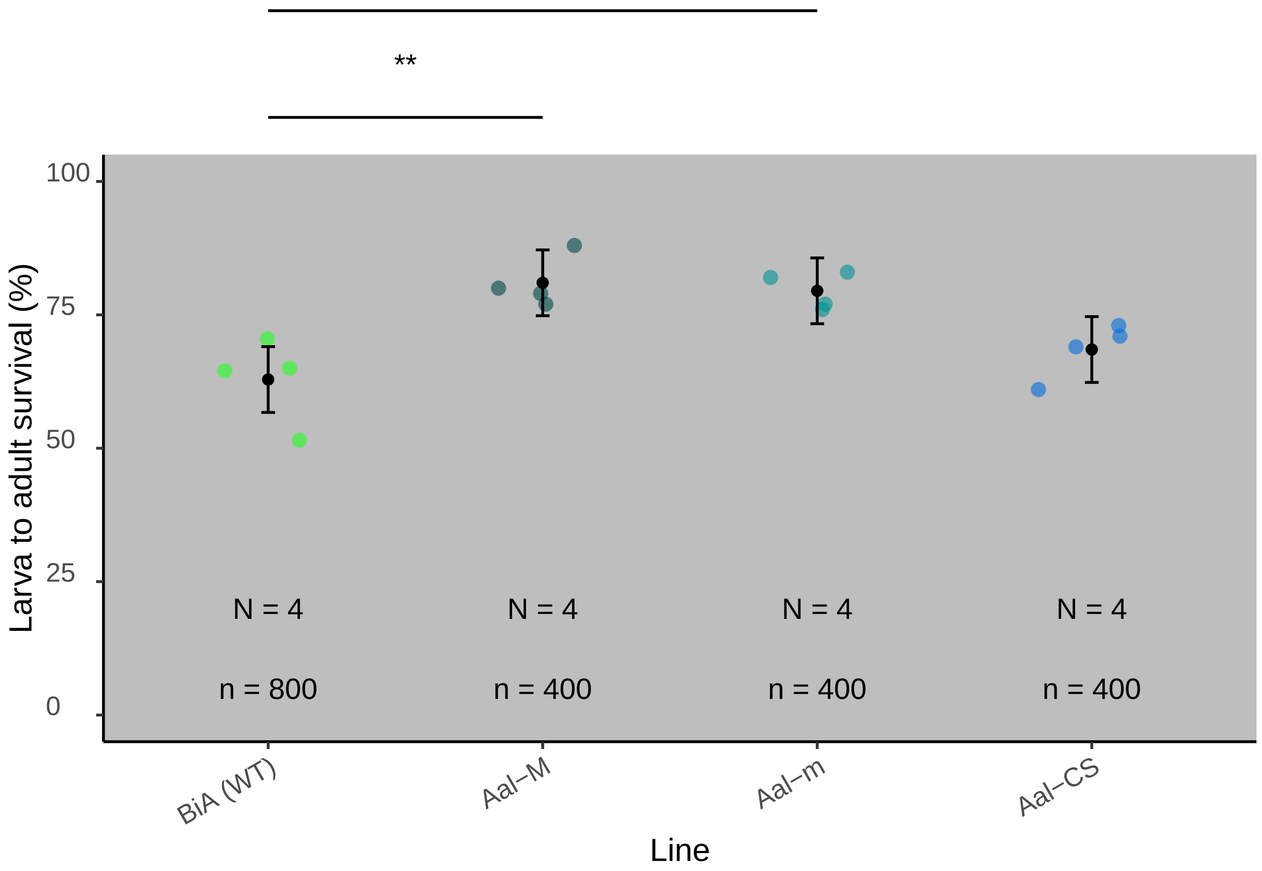
**
100
75
50
25
0
Larva to adult survival (%)
N = 4
n = 800
N = 4
n = 400
N = 4
n = 400
N = 4
n = 400
BiA (WT)
Aal–M
Aal–m
Aal–CS
Line

Figure 4

Competitiveness

Ae. aegypti

```
compet_aeg <- read.table("data/Competitiveness_aeg.csv",
                        header = TRUE, sep=",")
compet_aeg$total = compet_aeg$tsg + compet_aeg$no_tsg
compet_aeg$comp = compet_aeg$tsg / (compet_aeg$total) * 100
compet_aeg$line =  factor(x = compet_aeg$line,
                          levels = c('Expected','Aaeg-M', 'Aaeg-CS'))
compet_aeg
```

Data

```
       line Replicate  tsg no_tsg total     comp
1  Expected         1 1300   3700  5000 26.00000
2    Aaeg-M         1  632   1758  2390 26.44351
3    Aaeg-M         2  618   1670  2288 27.01049
4    Aaeg-M         3  740   1472  2212 33.45389
5    Aaeg-M         4  493   1775  2268 21.73721
6    Aaeg-M         5  522   1554  2076 25.14451
7   Aaeg-CS         1  101    486   587 17.20613
8   Aaeg-CS         2  411   1268  1679 24.47886
9   Aaeg-CS         3  459    847  1306 35.14548
10  Aaeg-CS         4  153    450   603 25.37313
11  Aaeg-CS         5  436   1033  1469 29.68005
```

```
compet.aeg =
  rbind(
    data.frame(replicate = compet_aeg[1,2],
               line = compet_aeg[1,1],
               result = rep(1, compet_aeg[1,3])),
    data.frame(replicate = compet_aeg[1,2],
               line = compet_aeg[1,1],
               result = rep(0, compet_aeg[1,4])))

for(i in 2:nrow(compet_aeg)){
  compet.aeg =
    rbind(
      compet.aeg,
      data.frame(replicate = compet_aeg[i,2],
                 line = compet_aeg[i,1],
                 result = rep(1, compet_aeg[i,3])),
      data.frame(replicate = compet_aeg[i,2],
                 line = compet_aeg[i,1],
                 result = rep(0, compet_aeg[i,4]))
    )
}
```

```
compet.aeg$line = factor(x = compet.aeg$line,
                         levels = c('Expected','Aaeg-M', 'Aaeg-CS'))
compet.aeg$Species = "Ae. aegypti"

sum.compet.aeg = compet.aeg %>%
  filter(line != 'Expected') %>%
  group_by(line) %>%
  dplyr::summarize(value = n(),
            N = max(replicate)) %>%
  data.frame() %>%
  mutate(pos = c(1,2))

head(compet.aeg)
```

```
  replicate     line result     Species
1         1 Expected      1 Ae. aegypti
2         1 Expected      1 Ae. aegypti
3         1 Expected      1 Ae. aegypti
4         1 Expected      1 Ae. aegypti
5         1 Expected      1 Ae. aegypti
6         1 Expected      1 Ae. aegypti
```

```
Expected_Aaeg_M = 0.26
Expected_Aaeg_CS = 0.26
```

Model .

```
# Generalized linear model with Bernoulli distribution
cpt.aeg = glm(formula = 'result ~ line',
             family = binomial,
             data = compet.aeg)
summary(cpt.aeg)
```

```
Call:
glm(formula = "result ~ line", family = binomial, data = compet.aeg)

Deviance Residuals:
    Min       1Q   Median       3Q      Max
-0.8044  -0.7890  -0.7890   1.6037   1.6414

Coefficients:
            Estimate Std. Error z value Pr(>|z|)
(Intercept) -1.04597    0.03224 -32.442   <2e-16 ***
lineAaeg-M   0.03858    0.03865   0.998   0.3182
lineAaeg-CS  0.08358    0.04388   1.905   0.0568 .
---
Signif. codes:  0 '***' 0.001 '**' 0.01 '*' 0.05 '.' 0.1 ' ' 1

(Dispersion parameter for binomial family taken to be 1)
```

```
    Null deviance: 25437  on 21877  degrees of freedom
Residual deviance: 25433  on 21875  degrees of freedom
AIC: 25439

Number of Fisher Scoring iterations: 4
```

```r
# Pairwise comparison
cpt.aeg.pred = ggpredict(cpt.aeg) %>% data.frame()

cpt.aeg.pred %>% dplyr::select(Line = line.x,
                               Mean = line.predicted,
                               SE = line.std.error,
                               CI.low = line.conf.low,
                               CI.high = line.conf.high)
```

```
      Line      Mean         SE    CI.low   CI.high
1 Expected 0.2600000 0.03224127 0.2480275 0.2723411
2   Aaeg-M 0.2674915 0.02131430 0.2593860 0.2757561
3  Aaeg-CS 0.2763997 0.02976378 0.2648858 0.2882179
```

Replication

```
     Line N     n
1  Aaeg-M 5 11234
2 Aaeg-CS 5  5644
```

Model fit quality

```r
check_model(cpt.aeg)
```

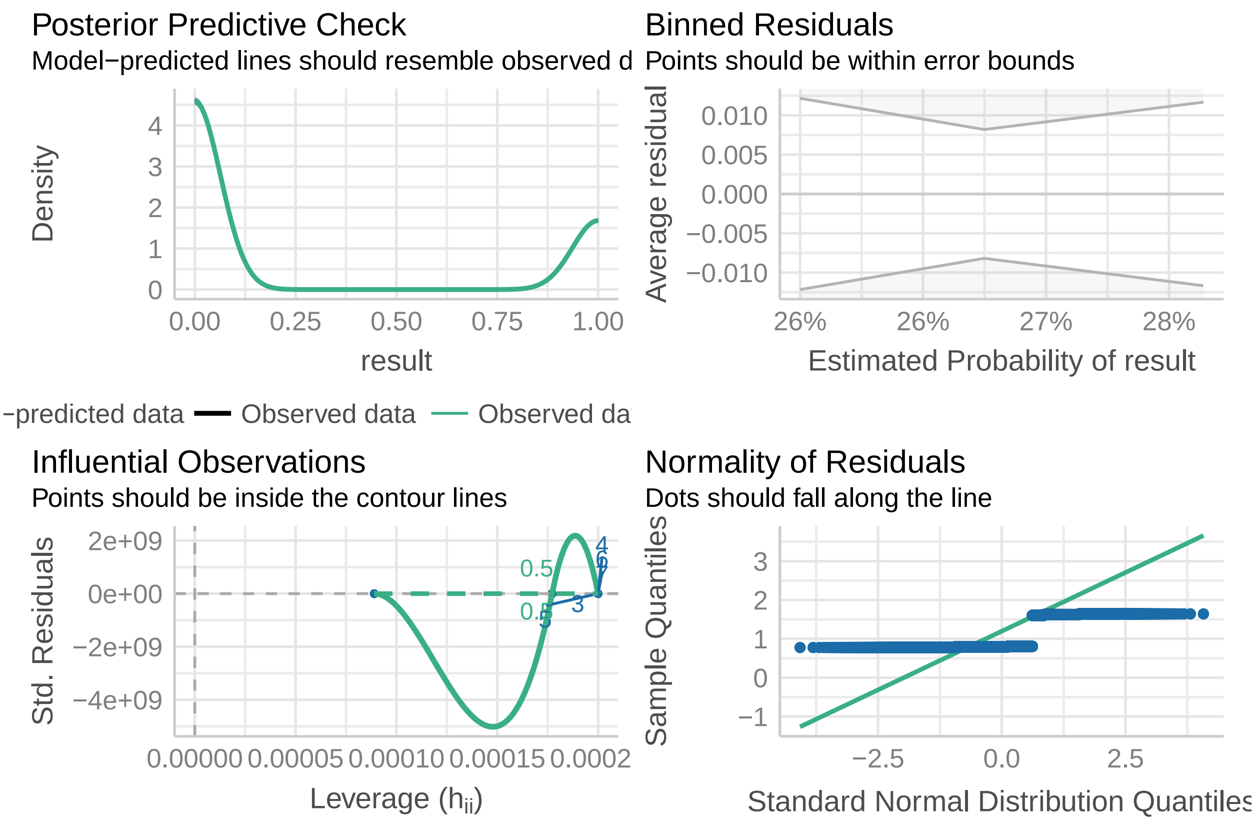

Plot .

```
comp.aeg.plot =
  ggplot(data =  NULL) +
  geom_point(data = compet_aeg[compet_aeg$line!='Expected',],
             aes(x = jitter(as.numeric(factor(line))),
                 y = comp,
                 color = line),
             size = 2, alpha = .6)+
  geom_point(data = cpt.aeg.pred[-1,],
             aes(x = (as.numeric(line.x)-1),
                 y = line.predicted * 100),
             size = 1.5, color = 'black') +
  geom_errorbar(data = cpt.aeg.pred[-1,],
                aes(x = (as.numeric(line.x)-1),
                    ymin = line.conf.low* 100,
                    ymax = line.conf.high* 100),
                width = 0.05, color = 'black') +
  annotate(geom = 'text', x = sum.compet.aeg$pos, y=85,
           label = paste0('n = ', sum.compet.aeg$value),
           size = 4) +
  annotate(geom = 'text', x= sum.compet.aeg$pos, y=95,
           label = c('N = 5', 'N = 5'),
           size = 4) +
  annotate(geom = 'line', x = c(.75, 1.25),
```

```
          y = rep(Expected_Aaeg_M*100, 2),
          lty = 2, color = 'grey30')  +
  annotate(geom = 'line', x = c(1.75, 2.25),
          y = rep(Expected_Aaeg_CS*100, 2),
          lty = 2, color = 'grey30') +
  annotate(geom = 'text', x = 2, y=50,
          label = 'n.s.',
          size = 4) +
  annotate(geom = 'text', x = 1, y=50,
          label = 'n.s.',
          size = 4) +
  scale_x_continuous(breaks = 1:2,
                     labels = c("Bra (WT) vs. Aaeg-M",
                                "Aaeg-CS vs. Aaeg-M"),
                     limits = c(.5,2.5)) +
  scale_y_continuous(limits = c(0,100),
                     breaks = c(0,25,50,75,100)) +
  scale_color_manual(values = line.color.aeg, labels = names(line.color.aeg)) +
  labs(x = 'Line ',
       y = 'Transgenic progeny (%)') +
  theme_classic() +
  theme(legend.position = 'none',
        axis.title.y = element_text(color = 'black',
                                    size = 12),
        axis.text.y = element_text(size = 10,
                                   hjust = 0, vjust = 0),
        axis.title.x = element_text(color = 'black',
                                    size = 12),
        axis.text.x = element_text(size = 10))


comp.aeg.plot
```

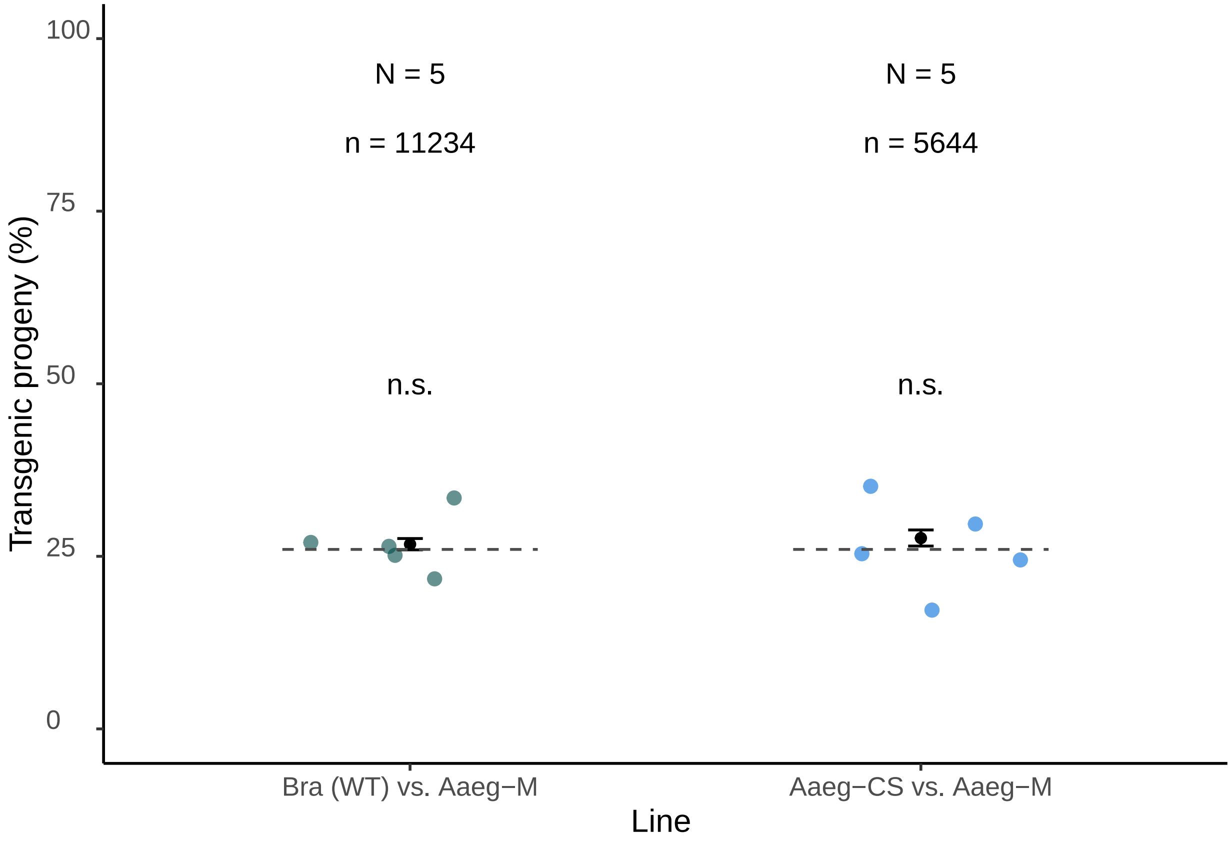
N = 5
n = 11234
N = 5
n = 5644
n.s.
n.s.
Transgenic progeny (%)
100
75
50
25
0
Bra (WT) vs. Aaeg−M
Aaeg−CS vs. Aaeg−M
Line

Ae. albopictus

Data .

```
compet_albo<-read.table("data/Competitiveness_albo.csv", header=TRUE, sep=",")
compet_albo$total = compet_albo$tsg + compet_albo$no_tsg
compet_albo$comp = compet_albo$tsg / (compet_albo$total) * 100
compet_albo$line =  factor(x = compet_albo$line,
                            levels = c('Expected','Aal-M', 'Aal-CS'))
compet_albo
```

```
       line Replicate tsg no_tsg total     comp
1  Expected         1 637   1863  2500 25.48000
2     Aal-M         1  75    185   260 28.84615
3     Aal-M         2  60    247   307 19.54397
4     Aal-M         3 119    335   454 26.21145
5     Aal-M         4 151    887  1038 14.54721
6     Aal-M         5  67    451   518 12.93436
7    Aal-CS         1 779   1405  2184 35.66850
8    Aal-CS         2 426   1980  2406 17.70574
9    Aal-CS         3 137    568   705 19.43262
10   Aal-CS         4 531   1136  1667 31.85363
11   Aal-CS         5 508   1539  2047 24.81681
```

```
compet.albo =
  rbind(
    data.frame(replicate = compet_albo[1,2],
               line = compet_albo[1,1],
               result = rep(1, compet_albo[1,3])),
    data.frame(replicate = compet_albo[1,2],
               line = compet_albo[1,1],
               result = rep(0, compet_albo[1,4])))

for(i in 2:nrow(compet_albo)){
  compet.albo =
    rbind(
      compet.albo,
      data.frame(replicate = compet_albo[i,2],
                 line = compet_albo[i,1],
                 result = rep(1, compet_albo[i,3])),
      data.frame(replicate = compet_albo[i,2],
                 line = compet_albo[i,1],
                 result = rep(0, compet_albo[i,4]))
    )
}

compet.albo$line = factor(x = compet.albo$line,
                            levels = c('Expected','Aal-M', 'Aal-CS'))
compet.albo$Species = "Ae. albopictus"

sum.compet.albo = compet.albo %>%
  filter(line != "Expected") %>%
  group_by(line) %>%
```

```r
  dplyr::summarize(value = n(),
            N = max(replicate)) %>%
  data.frame() %>%
  mutate(pos = c(1,2))

head(compet.albo)
```

```
  replicate     line result        Species
1         1 Expected      1 Ae. albopictus
2         1 Expected      1 Ae. albopictus
3         1 Expected      1 Ae. albopictus
4         1 Expected      1 Ae. albopictus
5         1 Expected      1 Ae. albopictus
6         1 Expected      1 Ae. albopictus
```

```r
Expected_Aal_M = 0.255
Expected_Aal_CS = 0.255
```

Model .

```r
# Generalized linear model with Bernoulli distribution
cpt.albo = glm(formula = 'result ~ line',
             family = binomial,
             data = compet.albo)
summary(cpt.albo)
```

```
Call:
glm(formula = "result ~ line", family = binomial, data = compet.albo)

Deviance Residuals:
    Min       1Q   Median       3Q      Max
-0.7835  -0.7835  -0.7670  -0.6361   1.8425

Coefficients:
            Estimate Std. Error z value Pr(>|z|)
(Intercept) -1.07317    0.04590 -23.382  < 2e-16 ***
lineAal-M   -0.42192    0.06856  -6.154 7.55e-10 ***
lineAal-CS   0.04939    0.05174   0.955     0.34
---
Signif. codes:  0 '***' 0.001 '**' 0.01 '*' 0.05 '.' 0.1 ' ' 1

(Dispersion parameter for binomial family taken to be 1)

    Null deviance: 15773  on 14085  degrees of freedom
Residual deviance: 15697  on 14083  degrees of freedom
AIC: 15703

Number of Fisher Scoring iterations: 4
```

```r
# Pairwise comparison
cpt.albo.pred = ggpredict(cpt.albo) %>% data.frame()

cpt.albo.pred %>% dplyr::select(Line = line.x,
                                Mean = line.predicted,
                                SE = line.std.error,
                                CI.low = line.conf.low,
                                CI.high = line.conf.high)
```

```
      Line      Mean         SE    CI.low   CI.high
1 Expected 0.2548000 0.04589797 0.2380986 0.2722543
2    Aal-M 0.1831587 0.05092841 0.1686943 0.1985671
3   Aal-CS 0.2642913 0.02389280 0.2552868 0.2734967
```

Replication

```
    Line N    n
1  Aal-M 5 2577
2 Aal-CS 5 9009
```

Model fit quality

```r
plot(cpt.albo)
```

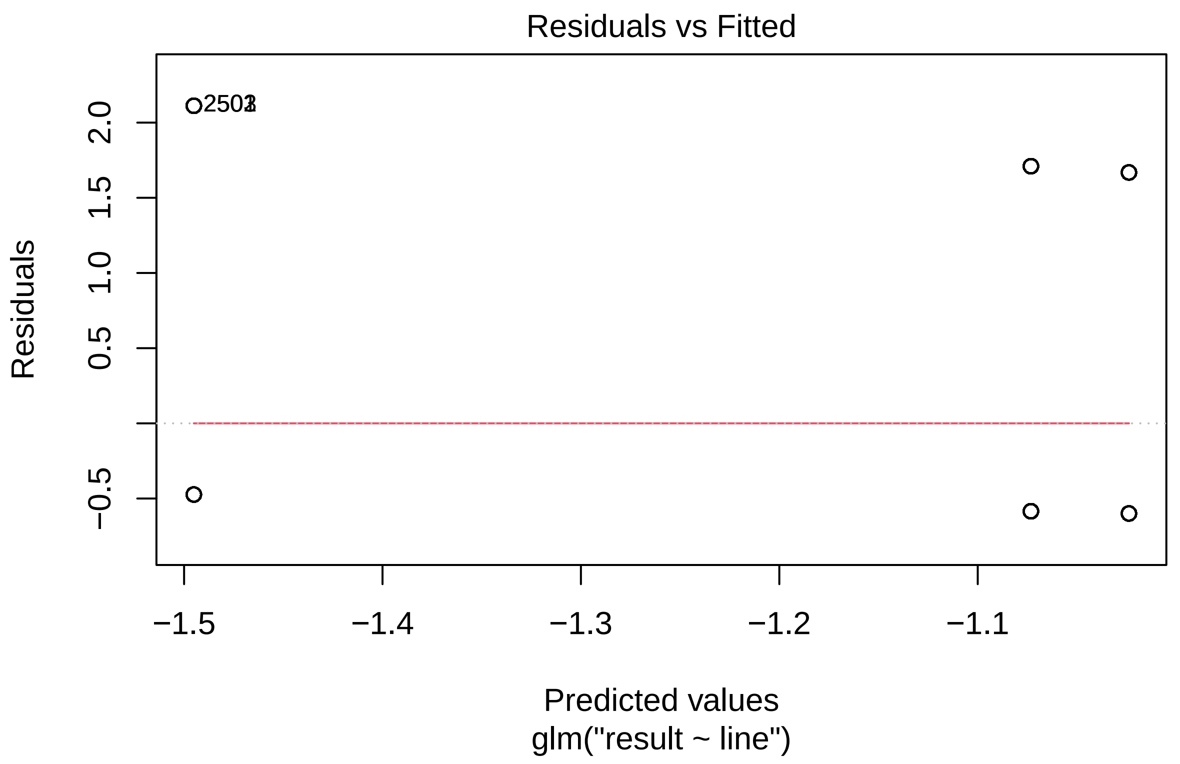

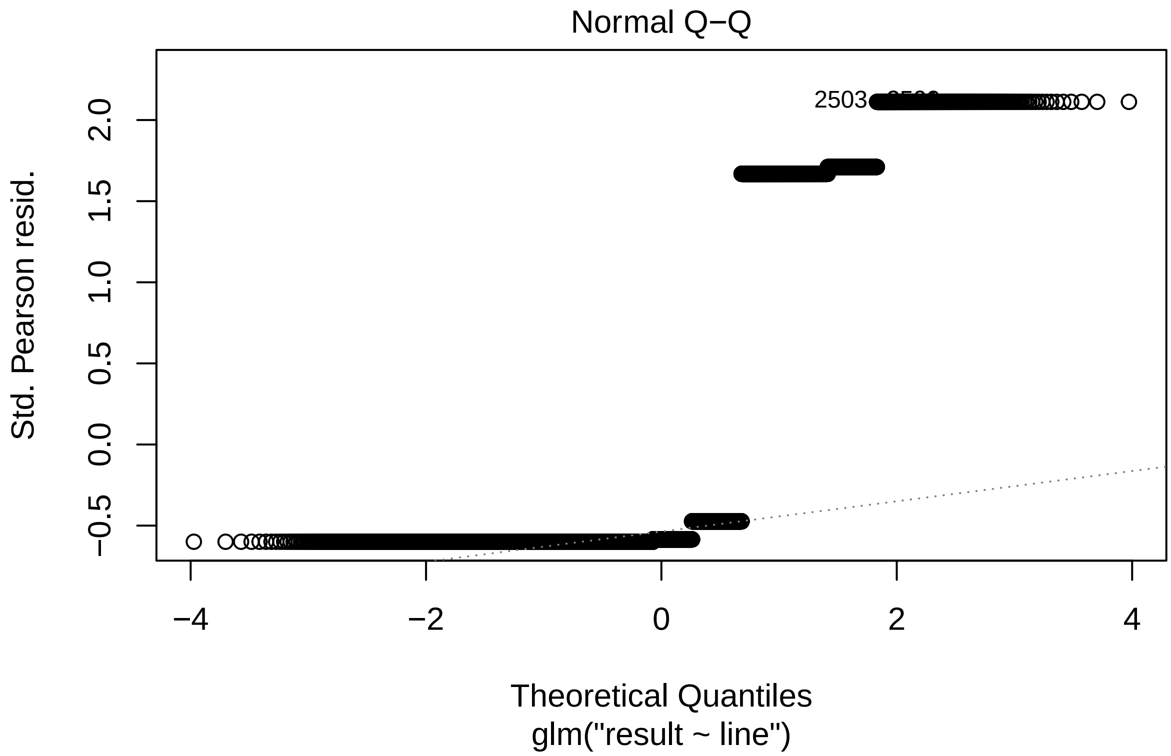
Normal Q−Q
Std. Pearson resid.
−0.5
0.0
0.5
1.0
1.5
2.0
−4
−2
0
2
4
2503
Theoretical Quantiles
glm("result ~ line")

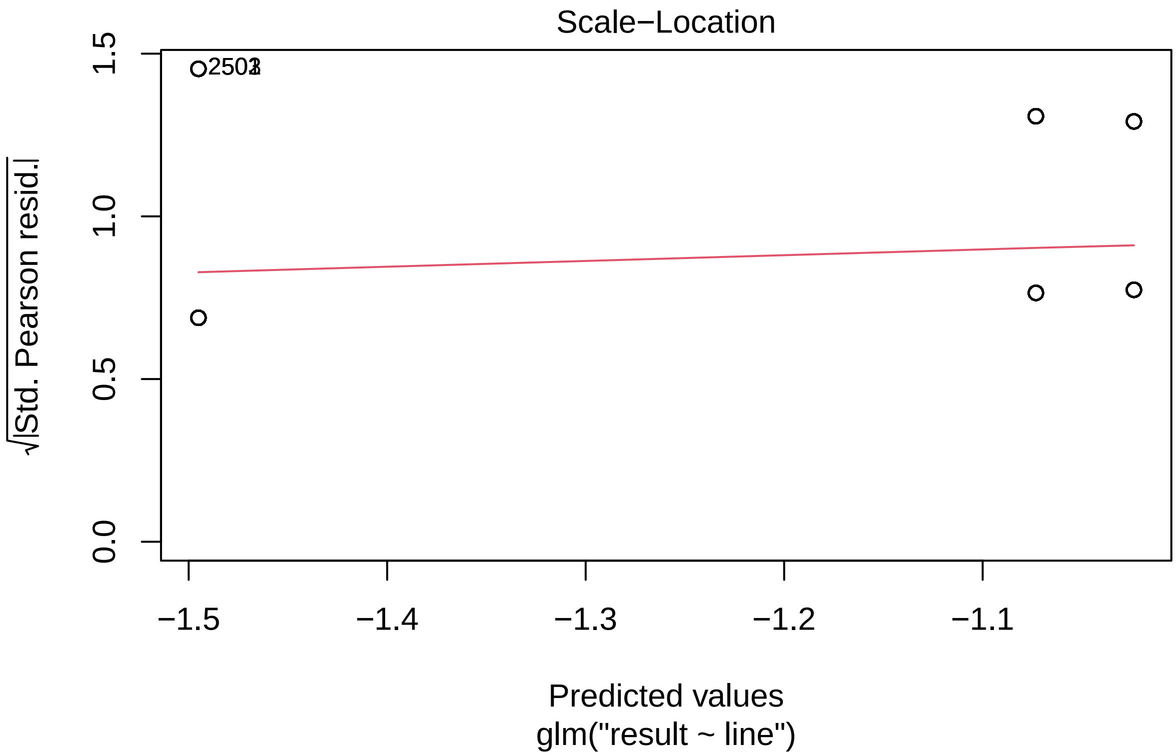
Scale−Location
√|Std. Pearson resid.|
0.0
0.5
1.0
1.5
2503
−1.5
−1.4
−1.3
−1.2
−1.1
Predicted values
glm("result ~ line")

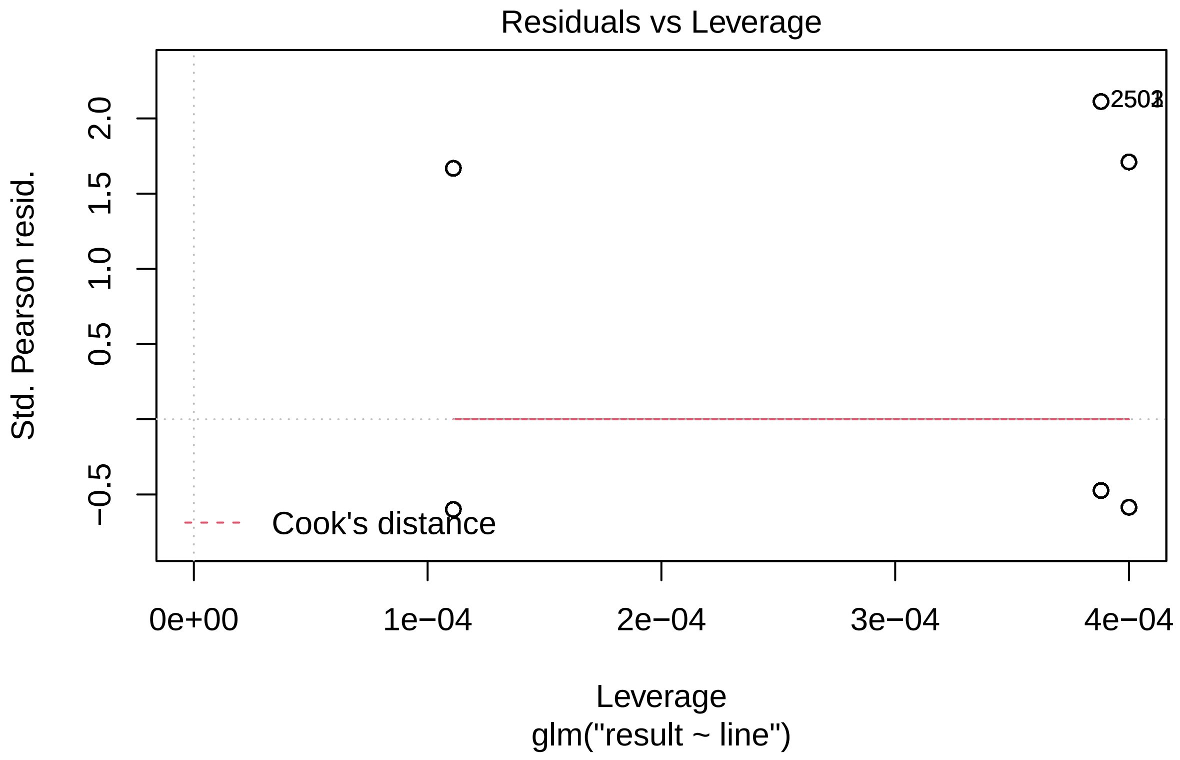

Plot .

```
comp.albo.plot =
  ggplot(data =  NULL) +
  geom_point(data = compet_albo[compet_albo$line!='Expected',],
             aes(x = jitter(as.numeric(factor(line))),
                 y = comp,
                 color = line),
             size = 2, alpha = .6)+
  geom_point(data = cpt.albo.pred[-1,],
             aes(x = (as.numeric(line.x)-1),
                 y = line.predicted * 100),
             size = 1.5,
             color = 'black') +
  geom_errorbar(data = cpt.albo.pred[-1,],
                aes(x = (as.numeric(line.x)-1),
                    ymin = line.conf.low* 100,
                    ymax = line.conf.high* 100),
                width = 0.05, color = 'black') +
  annotate(geom = 'text',
           x = sum.compet.albo$pos, y = 85,
           label = paste0('n = ', sum.compet.albo$value),
           size = 4) +
  annotate(geom = 'text',
           x = sum.compet.albo$pos, y = 95,
```

```
           label = c('N = 5', 'N = 5'),
           size = 4) +
  annotate(geom = 'line', x = c(.75, 1.25),
           y=rep(Expected_Aal_M*100, 2),
           lty = 2, color = 'grey30')  +
  annotate(geom = 'line', x = c(1.75, 2.25),
           y=rep(Expected_Aal_CS*100, 2),
           lty = 2, color = 'grey30') +
  annotate(geom = 'text', x = 2, y = 50,
           label = 'n.s.',
           size = 4) +
  annotate(geom = 'text', x = 1, y = 50,
           label = '***',
           size = 4) +
  scale_x_continuous(breaks = 1:2,
                     labels = c("BiA (WT) vs. Aal-M",
                                "Aal-CS vs. Aal-M"),
                     limits = c(.5,2.5)) +
  scale_y_continuous(limits = c(0,100),
                     breaks = c(0,25,50,75,100)) +
  scale_color_manual(values = line.color.alb, labels = names(line.color.alb)) +
  labs(x = 'Line ',
       y = 'Transgenic progeny (%)') +
  theme_classic() +
  theme(legend.position = 'none',
        panel.background = element_rect(fill = 'grey'),
        axis.title.y = element_text(color = 'black',
                                    size = 12),
        axis.text.y = element_text(size = 10,
                                   hjust = 0, vjust = 0),
        axis.title.x = element_text(color = 'black',
                                    size = 12),
        axis.text.x = element_text(size = 10))

comp.albo.plot
```

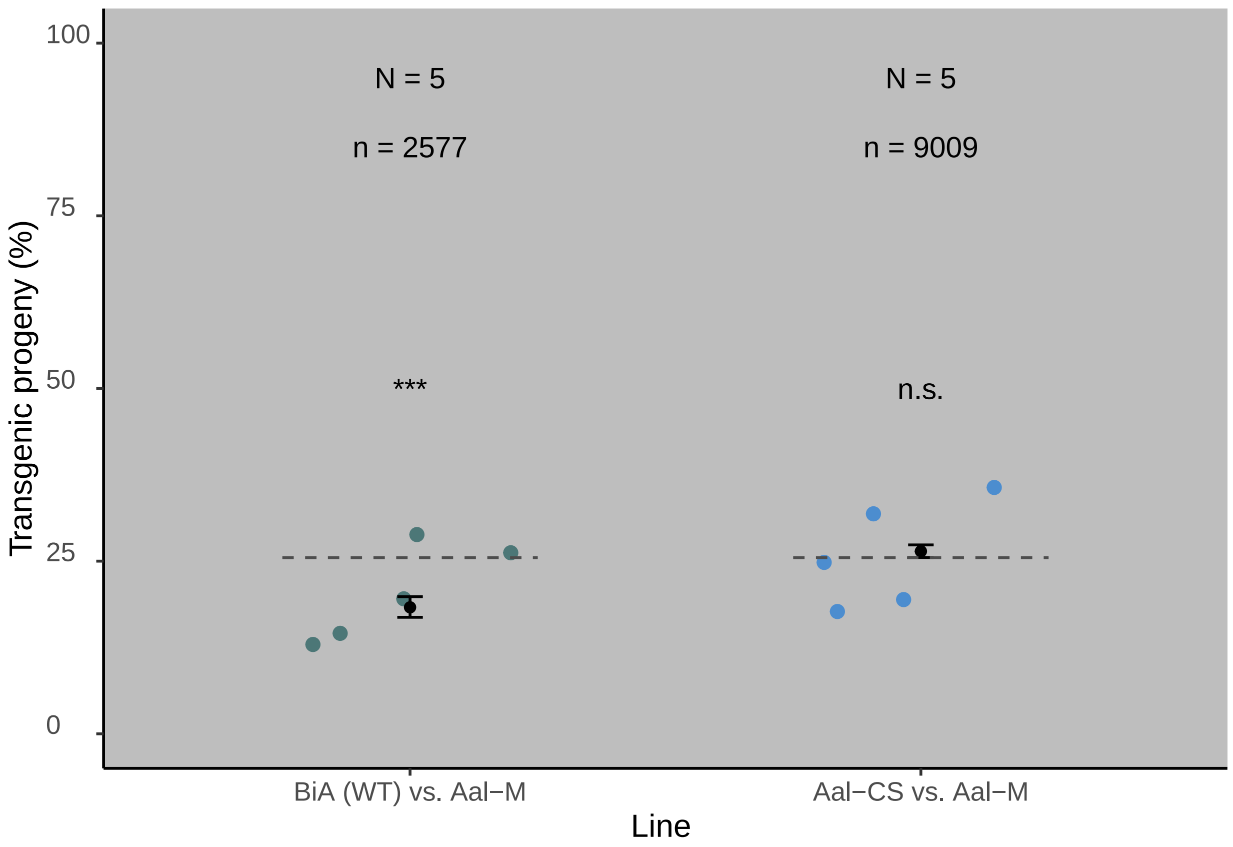

N = 5
n = 2577
***
N = 5
n = 9009
n.s.
Transgenic progeny (%)
100
75
50
25
0
BiA (WT) vs. Aal−M
Aal−CS vs. Aal−M
Line

Flight test

Ae. aegypti

Data .

```
flight_aegypti<-read.table("data/Flight_aeg.csv",
                           header = TRUE, sep = ";")

flight_aegypti$esc = flight_aegypti$out /
  (flight_aegypti$out + flight_aegypti$in.) * 100

flight_aegypti$Line = factor(flight_aegypti$Line,
                             levels = c("Bra (WT)", "Aaeg-M", "Aaeg-CS"))

flight_aegypti
```

```
  Replicate     Line out in.      esc
1         2   Aaeg-M  65  36 64.35644
2         2  Aaeg-CS  66  36 64.70588
3         2 Bra (WT)  71  22 76.34409
4         3   Aaeg-M  57  31 64.77273
5         3  Aaeg-CS  75  19 79.78723
6         3 Bra (WT)  64  35 64.64646
7         4   Aaeg-M  76  21 78.35052
8         4  Aaeg-CS  79  27 74.52830
9         4 Bra (WT)  78  19 80.41237
```

```
flight.aeg =
  rbind(
    data.frame(replicate = flight_aegypti[1,1],
               treatment = flight_aegypti[1,2],
               result = rep(1, flight_aegypti[1,3])),
    data.frame(replicate = flight_aegypti[1,1],
               treatment = flight_aegypti[1,2],
               result = rep(0, flight_aegypti[1,4])))

for(i in 2:nrow(flight_aegypti)){
  flight.aeg =
    rbind(
      flight.aeg,
      data.frame(replicate = flight_aegypti[i,1],
                 treatment = flight_aegypti[i,2],
                 result = rep(1, flight_aegypti[i,3])),
      data.frame(replicate = flight_aegypti[i,1],
                 treatment = flight_aegypti[i,2],
                 result = rep(0, flight_aegypti[i,4]))
    )
}

flight.aeg$Species = 'Ae. aegypti'
flight.aeg$treatment = factor(flight.aeg$treatment,
                              levels = c("Bra (WT)", "Aaeg-M", "Aaeg-CS"))
```

```r
head(flight.aeg)
```

```
  replicate treatment result     Species
1         2    Aaeg-M      1 Ae. aegypti
2         2    Aaeg-M      1 Ae. aegypti
3         2    Aaeg-M      1 Ae. aegypti
4         2    Aaeg-M      1 Ae. aegypti
5         2    Aaeg-M      1 Ae. aegypti
6         2    Aaeg-M      1 Ae. aegypti
```

```r
flight.aeg.summary = flight.aeg %>% group_by(treatment) %>%
  dplyr::summarise(value = n()) %>% data.frame() %>%
  mutate(pos = 1:3)

head(flight.aeg.summary)
```

```
  treatment value pos
1  Bra (WT)   289   1
2    Aaeg-M   286   2
3   Aaeg-CS   302   3
```

Model .

```r
flight.aeg.glmer = glmer(formula = 'result ~ treatment + (1|replicate)',
                         data = flight.aeg, family = binomial)

summary(flight.aeg.glmer)
```

```
Generalized linear mixed model fit by maximum likelihood (Laplace
  Approximation) [glmerMod]
 Family: binomial  ( logit )
Formula: result ~ treatment + (1 | replicate)
   Data: flight.aeg

     AIC      BIC   logLik deviance df.resid
  1045.4   1064.5   -518.7   1037.4      873

Scaled residuals:
    Min      1Q  Median      3Q     Max
-1.8340 -1.4218  0.6088  0.6315  0.7033

Random effects:
 Groups    Name        Variance Std.Dev.
 replicate (Intercept) 0.02668  0.1633
Number of obs: 877, groups:  replicate, 3

Fixed effects:
                 Estimate Std. Error z value Pr(>|z|)
(Intercept)       1.03584    0.16391   6.320 2.62e-10 ***
treatmentAaeg-M  -0.22027    0.18562  -1.187    0.235
treatmentAaeg-CS -0.04688    0.18647  -0.251    0.801
```

```
---
Signif. codes:  0 '***' 0.001 '**' 0.01 '*' 0.05 '.' 0.1 ' ' 1

Correlation of Fixed Effects:
            (Intr) trtA-M
tretmntAg-M -0.590
trtmntAg-CS -0.587  0.519
```

```r
pairewise.f.aeg = glht(flight.aeg.glmer,
                       mcp(treatment="Tukey"))

summary(pairewise.f.aeg)
```

```
	 Simultaneous Tests for General Linear Hypotheses

Multiple Comparisons of Means: Tukey Contrasts


Fit: glmer(formula = result ~ treatment + (1 | replicate), data = flight.aeg,
    family = binomial)

Linear Hypotheses:
                        Estimate Std. Error z value Pr(>|z|)
Aaeg-M - Bra (WT) == 0  -0.22027    0.18562  -1.187    0.461
Aaeg-CS - Bra (WT) == 0 -0.04688    0.18647  -0.251    0.966
Aaeg-CS - Aaeg-M == 0    0.17340    0.18254   0.950    0.609
(Adjusted p values reported -- single-step method)
```

Replication

```
# A tibble: 3 x 3
  Line         N     n
  <fct>     <int> <int>
1 Bra (WT)     3   289
2 Aaeg-M       3   286
3 Aaeg-CS      3   302
```

Model fit quality

```r
check_model(flight.aeg.glmer)
```

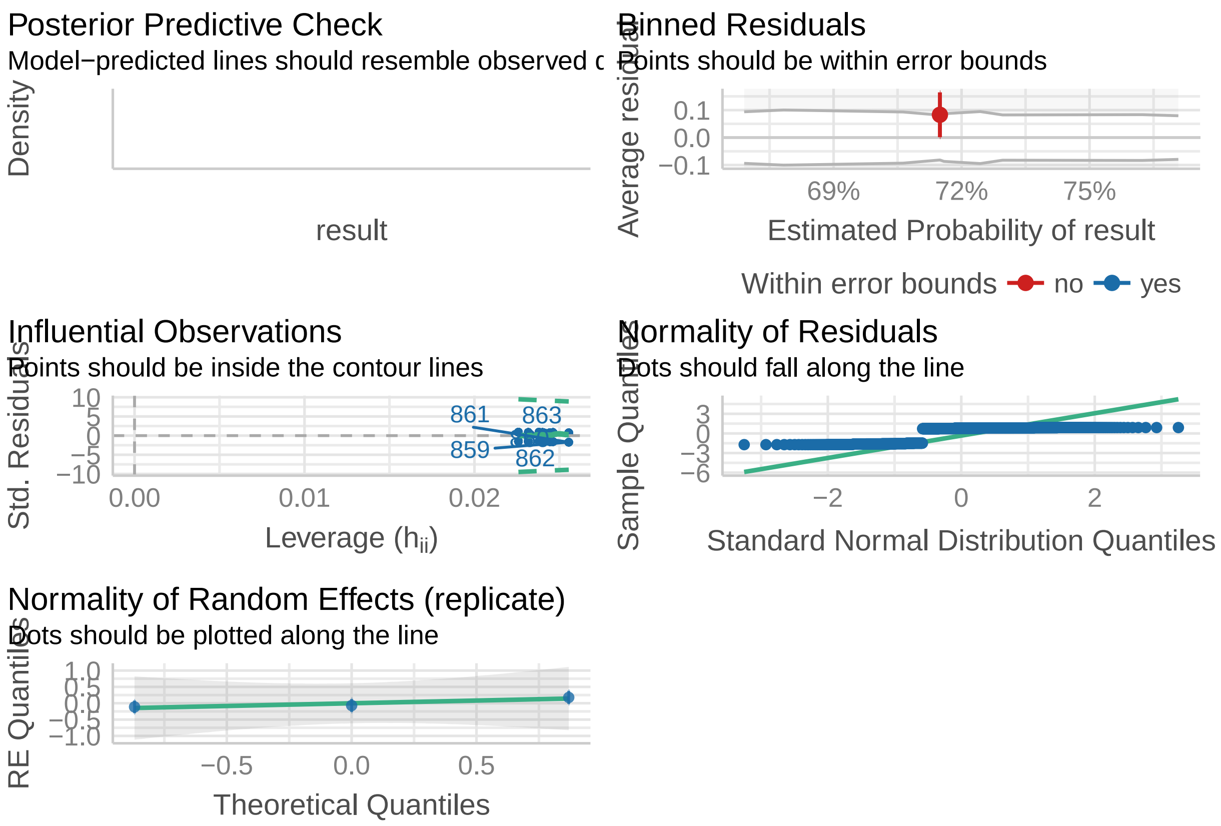

Plot .

```
ft.aeg = data.frame(ggpredict(model = flight.aeg.glmer,
                              terms = c("treatment")))
ft.aeg$x = factor(ft.aeg$x,
                  levels = c("Bra (WT)", "Aaeg-M", "Aaeg-CS"))
ft.aeg$Species = 'Ae. aegypti'

ft.plot.aeg =
  ggplot(data =  NULL) +
  geom_point(data = flight_aegypti,
             aes(x = jitter(as.numeric(factor(Line))),
                 y = esc,
                 color = Line),
             size = 2, alpha = .6)+
  geom_point(data = ft.aeg,
             aes(x = as.numeric(x),
                 y = predicted * 100),
             size = 1.5, color = 'black') +
  geom_errorbar(data = ft.aeg,
                aes(x = as.numeric(x),
                    ymin = conf.low* 100,
                    ymax = conf.high* 100),
                width = 0.05, color = 'black') +
  annotate(geom = 'text',
```

```r
           x = flight.aeg.summary$pos, y = 5,
           label = paste0('n = ', flight.aeg.summary$value),
           size = 4) +
  annotate(geom = 'text',
           x = flight.aeg.summary$pos, y = 15,
           label = 'N = 3',
           size = 4) +
  annotate(geom = 'line', x = 1:3, y = 125) +
  annotate(geom = 'text', x = 2, y = 132,
           label = 'n.s.',
           size = 4) +
  annotate(geom = 'line', x = 1:2, y = 108) +
  annotate(geom = 'text', x = 1.5, y = 115,
           label = 'n.s.',
           size = 4) +
  scale_x_continuous(breaks = 1:3,
                     labels = c("Bra (WT)",
                                "Aaeg-M",
                                "Aaeg-CS"),
                     limits = c(.5,3.5)) +
  scale_y_continuous(breaks = c(0,25,50,75,100)) +
  scale_color_manual(values = line.color.aeg, labels = names(line.color.aeg)) +
  coord_cartesian(clip = 'off', ylim = c(0,100))+
  labs(x = 'Line',
       y = 'Escaped males (%)') +
  theme_classic() +
  theme(legend.position = 'none',
        plot.margin = unit(c(1.5,0,0,0), 'cm'),
        axis.title.y = element_text(color = 'black',
                                    size = 12),
        axis.text.y = element_text(size = 11,
                                   hjust = 0, vjust = 0),
        axis.title.x = element_text(color = 'black',
                                    size = 12),
        axis.text.x = element_text(size = 11))

ft.plot.aeg
```

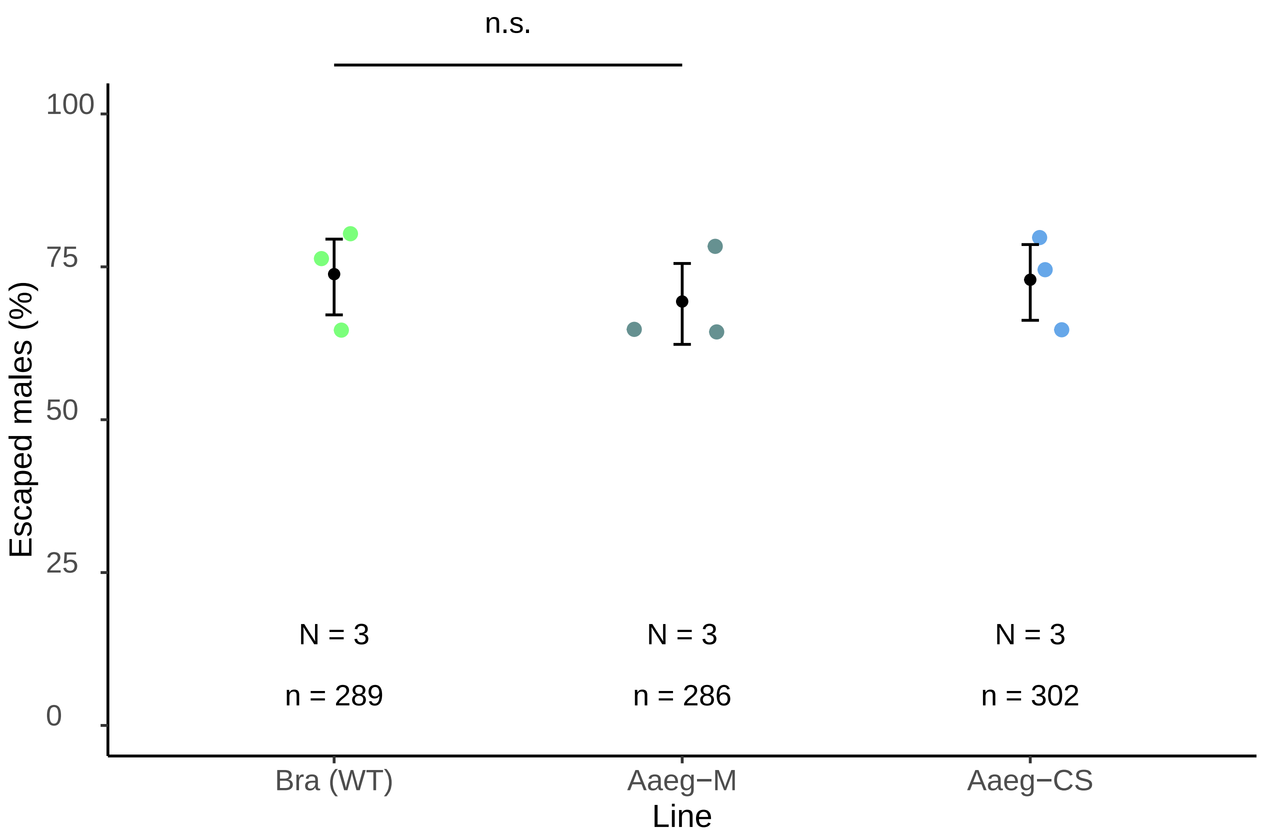

n.s.
100
75
50
25
0
Escaped males (%)
N = 3
n = 289
N = 3
n = 286
N = 3
n = 302
Bra (WT)
Aaeg−M
Aaeg−CS
Line

Ae. albopictus

Data .

```
flight_albo<-read.table("data/Flight_albo.csv",
                        header = TRUE, sep = ";")

flight_albo$esc = flight_albo$out / (flight_albo$out + flight_albo$in.) * 100

flight_albo$Line = factor(flight_albo$Line,
                          levels = c("BiA (WT)", "Aal-M", "Aal-CS"))

head(flight_albo)
```

```
  Replicate     Line out in.      esc
1         2    Aal-M  26  63 29.21348
2         2   Aal-CS  45  48 48.38710
3         2 BiA (WT)  38  48 44.18605
4         3    Aal-M  57  31 64.77273
5         3   Aal-CS  62  16 79.48718
6         3 BiA (WT)  58  31 65.16854
```

```
flight.albo =
  rbind(
    data.frame(replicate = flight_albo[1,1],
               treatment = flight_albo[1,2],
               result = rep(1, flight_albo[1,3])),
    data.frame(replicate = flight_albo[1,1],
               treatment = flight_albo[1,2],
               result = rep(0, flight_albo[1,4])))

for(i in 2:nrow(flight_albo)){
  flight.albo =
    rbind(
      flight.albo,
      data.frame(replicate = flight_albo[i,1],
                 treatment = flight_albo[i,2],
                 result = rep(1, flight_albo[i,3])),
      data.frame(replicate = flight_albo[i,1],
                 treatment = flight_albo[i,2],
                 result = rep(0, flight_albo[i,4]))
    )
}

flight.albo$Species = 'Ae. albopictus'
flight.albo$treatment = factor(flight.albo$treatment,
                               levels = c("BiA (WT)", "Aal-M", "Aal-CS"))

head(flight.albo)
```

```
  replicate treatment result        Species
1         2     Aal-M      1 Ae. albopictus
2         2     Aal-M      1 Ae. albopictus
```

```
3         2     Aal-M      1 Ae. albopictus
4         2     Aal-M      1 Ae. albopictus
5         2     Aal-M      1 Ae. albopictus
6         2     Aal-M      1 Ae. albopictus
```

```
flight.alb.summary = flight.albo %>% group_by(treatment) %>%
  dplyr::summarise(value = n()) %>% data.frame() %>%
  mutate(pos = 1:3)

head(flight.alb.summary)
```

```
  treatment value pos
1  BiA (WT)   264   1
2     Aal-M   273   2
3    Aal-CS   244   3
```

Model .

```
mod.glmer.albo = glmer(formula = 'result ~ treatment + (1|replicate)',
                       data = flight.albo, family = binomial)

summary(mod.glmer.albo)
```

```
Generalized linear mixed model fit by maximum likelihood (Laplace
  Approximation) [glmerMod]
 Family: binomial  ( logit )
Formula: result ~ treatment + (1 | replicate)
   Data: flight.albo

     AIC      BIC   logLik deviance df.resid
  1036.3   1054.9   -514.1   1028.3      777

Scaled residuals:
    Min      1Q  Median      3Q     Max
-1.7839 -0.9200  0.5606  0.9063  1.4280

Random effects:
 Groups    Name        Variance Std.Dev.
 replicate (Intercept) 0.2407   0.4906
Number of obs: 781, groups:  replicate, 3

Fixed effects:
                Estimate Std. Error z value Pr(>|z|)
(Intercept)       0.2196     0.3106   0.707   0.4795
treatmentAal-M   -0.3636     0.1784  -2.038   0.0415 *
treatmentAal-CS   0.3490     0.1867   1.869   0.0616 .
---
Signif. codes:  0 '***' 0.001 '**' 0.01 '*' 0.05 '.' 0.1 ' ' 1

Correlation of Fixed Effects:
            (Intr) trtA-M
tretmntAl-M -0.293
trtmntAl-CS -0.279  0.485
```

```
pairewise.f.albo = glht(mod.glmer.albo,
                        mcp(treatment="Tukey"))

summary(pairewise.f.albo)
```

```

     Simultaneous Tests for General Linear Hypotheses

Multiple Comparisons of Means: Tukey Contrasts


Fit: glmer(formula = result ~ treatment + (1 | replicate), data = flight.albo,
    family = binomial)

Linear Hypotheses:
                       Estimate Std. Error z value Pr(>|z|)
Aal-M - BiA (WT) == 0   -0.3636     0.1784  -2.038 0.103122
Aal-CS - BiA (WT) == 0   0.3490     0.1867   1.869 0.147542
Aal-CS - Aal-M == 0      0.7126     0.1854   3.842 0.000341 ***
---
Signif. codes:  0 '***' 0.001 '**' 0.01 '*' 0.05 '.' 0.1 ' ' 1
(Adjusted p values reported -- single-step method)
```

Replication

```
# A tibble: 3 x 3
  Line         N     n
  <fct>    <int> <int>
1 BiA (WT)     3   264
2 Aal-M        3   273
3 Aal-CS       3   244
```

Model fit quality

```
check_model(mod.glmer.albo)
```

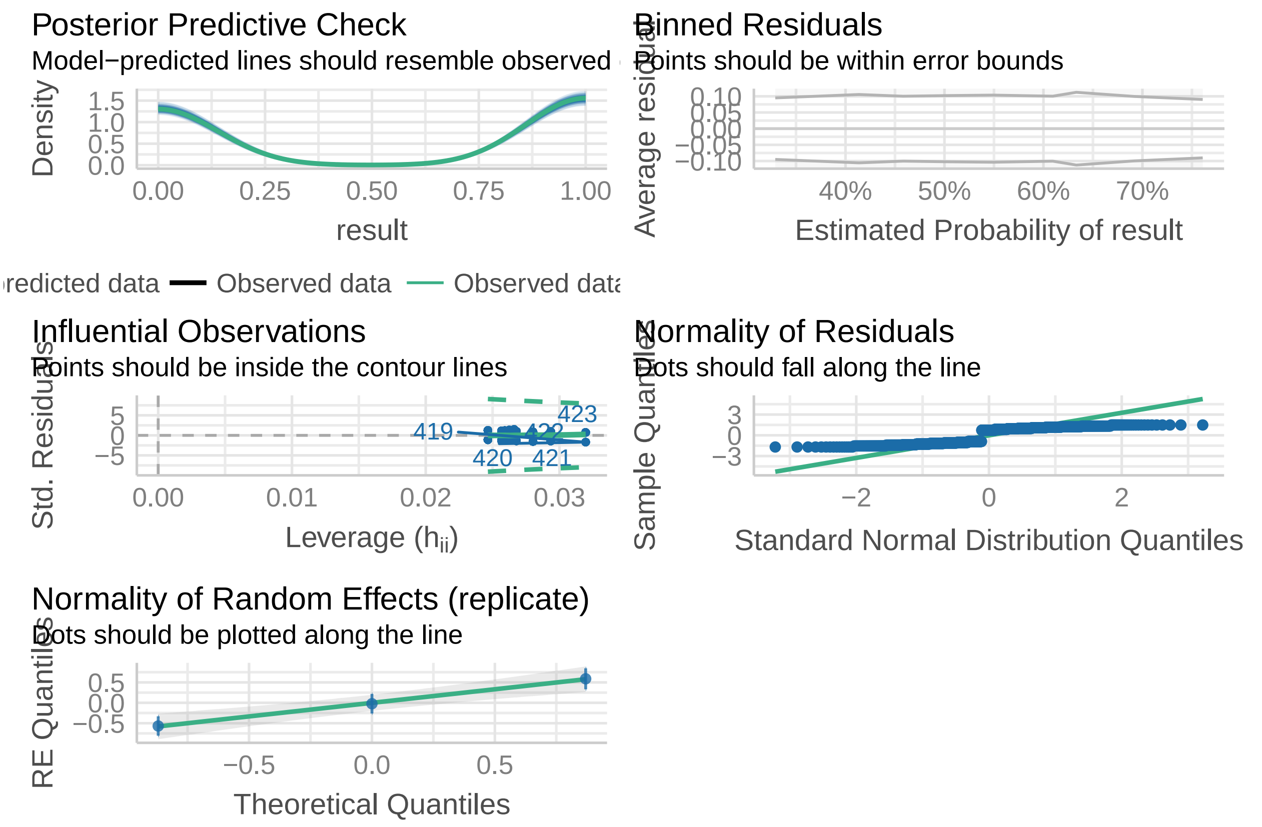

Plot .

```
ft.albo = data.frame(ggpredict(model = mod.glmer.albo, terms = c("treatment")))
ft.albo$Specft.albo$Species = 'Ae. albopictus'

ft.plot.alb =
  ggplot(data =  NULL) +
  geom_point(data = flight_albo,
             aes(x = jitter(as.numeric(factor(Line))),
                 y = esc, color = Line),
             size = 2, alpha = .6) +
  geom_point(data = ft.albo,
             aes(x = as.numeric(x),
                 y = predicted * 100),
             size = 1.5, color = 'black') +
  geom_errorbar(data = ft.albo,
                aes(x = as.numeric(x),
                    ymin = conf.low * 100,
                    ymax = conf.high * 100),
                width = 0.05, color = 'black') +
  annotate(geom = 'text',
           x = flight.alb.summary$pos, y = 5,
           label = paste0('n = ', flight.alb.summary$value),
           size = 4) +
  annotate(geom = 'text',
```

```
           x = flight.alb.summary$pos, y = 15,
           label = 'N = 3',
           size = 4) +
  annotate(geom = 'line', x = 1:3, y = 125) +
  annotate(geom = 'text', x = 2, y = 132,
           label = 'n.s.',
           size = 4) +
  annotate(geom = 'line', x = 1:2, y = 108) +
  annotate(geom = 'text', x = 1.5, y = 115,
           label = 'n.s.',
           size = 4) +
  scale_x_continuous(breaks = 1:3,
                     labels = c("BiA (WT)",
                                "Aal-M",
                                "Aal-CS"),
                     limits = c(.5,3.5)) +
  scale_y_continuous(breaks = c(0,25,50,75,100)) +
  coord_cartesian(clip = 'off', ylim = c(0,100)) +
  scale_color_manual(values = line.color.alb,
                     labels = names(line.color.alb)) +
  labs(x = 'Line',
       y = 'Escaped males (%)') +
  theme_classic() +
   theme(legend.position = 'none',
        plot.margin = unit(c(1.5,0,0,0), 'cm'),
        axis.title.y = element_text(color = 'black',
                                    size = 12),
        axis.text.y = element_text(size = 11,
                                   hjust = 0, vjust = 0),
        axis.title.x = element_text(color = 'black',
                                    size = 12),
        axis.text.x = element_text(size = 11),
        panel.background = element_rect(fill = 'grey'))

ft.plot.alb
```

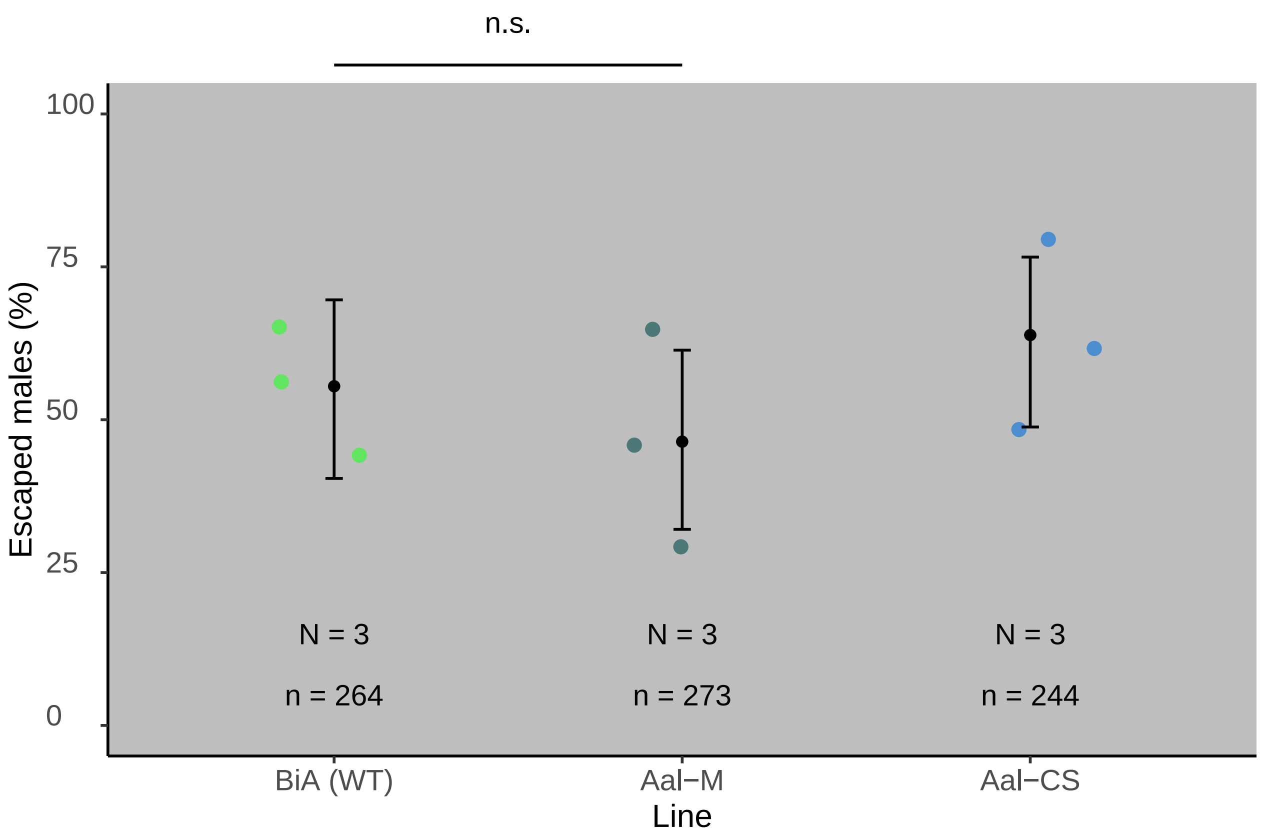
n.s.
100
75
50
25
0
Escaped males (%)
N = 3
n = 264
N = 3
n = 273
N = 3
n = 244
BiA (WT)
Aal−M
Aal−CS
Line

Adult survival

Ae. aegypti

Data .

```
data_aeg <- read.table("data/Survival_aeg.txt", h = T)
data_aeg$Line[data_aeg$Line=='Bra-(WT)'] = 'Bra (WT)'
data_aeg$Line = factor(data_aeg$Line,
                       levels = c('Bra (WT)', 'Aaeg-M','Aaeg-CS'))

data_aeg |> dplyr::select(-(3:5))
```

```
   Replicate     Line male_adults D1 D2 D3 D4 D5 D6 D7 D8 D9 D10 D11 D12 D13
1          1   Aaeg-M          87  0  0  0  0  0  0  0  0  0   0   0   0   0
2          2   Aaeg-M          82  0  0  0  0  0  0  0  0  0   0   0   0   0
3          3   Aaeg-M          93  1  0  0  0  1  0  0  0  0   0   0   0   0
4          4   Aaeg-M          73  0  0  0  0  0  0  0  1  0   0   0   0   0
5          1 Bra (WT)          82  0  0  0  0  0  0  0  0  0   0   0   0   1
6          2 Bra (WT)          97  0  0  0  0  2  0  0  0  0   0   0   0   0
7          3 Bra (WT)          90  0  1  0  0  0  0  0  0  0   1   0   0   0
8          4 Bra (WT)         102  0  1  0  0  0  0  0  0  0   1   0   0   0
9          5 Bra (WT)         105  2  0  0  0  0  1  1  0  0   0   0   1   0
10         6 Bra (WT)         105  3  0  0  0  2  0  0  0  0   0   0   0   0
11         7 Bra (WT)         105  0  0  0  0  0  0  0  0  0   0   0   0   1
12         8 Bra (WT)         105  0  0  0  0  0  0  1  0  0   0   0   0   0
13         5   Aaeg-M         105  1  0  0  0  0  0  0  0  0   0   0   0   0
14         6   Aaeg-M         105  2  1  0  0  0  0  0  0  0   0   0   0   0
15         7   Aaeg-M         105  1  0  0  0  0  0  2  0  0   0   1   0   0
16         8   Aaeg-M         105  0  0  0  0  1  0  0  2  0   0   0   0   0
17         1  Aaeg-CS         105  0  0  0  0  0  0  0  0  0   0   1   0   0
18         2  Aaeg-CS         105  0  1  0  0  0  0  0  0  0   0   0   0   0
19         3  Aaeg-CS         105  0  0  0  0  1  0  0  0  0   0   0   0   0
20         4  Aaeg-CS         105  0  1  0  0  0  0  0  0  0   0   0   0   0
   D14
1    0
2    0
3    0
4    1
5    0
6    0
7    0
8    0
9    0
10   0
11   0
12   0
13   0
14   0
15   0
16   0
17   0
18   1
19   0
```

20 0

```
# First, melt the data.frame
data1 <- melt(data_aeg ,
              .(Replicate,Line, larvae, adults, larval_deaths, male_adults))

# Transform days in numbers
data1$variable <- as.numeric(str_replace(data1$variable, "D",""))

#Sort by Line, Replicate and variable.
data2 <- arrange(data1, Line, Replicate, variable )
event <- subset(data2, value>0)

dat <- NULL
temporary_event <- NULL
for (i in 1:nrow(event)) {
  N <- event[i, "value"]
  dat <- data.frame(Replicate=rep(event[i,"Replicate"],N) ,Line=rep(event[i,"Line"],N) ,larvae=rep(even

  temporary_event  <- rbind(temporary_event ,dat)
}

dat <- data.frame(Replicate = 1, Line = "Aaeg-M", larvae = 100,
                  adults = 87, larval_deaths = 13,
                  male_adults = 87, Day = 14, Event = 0)
dat1 <- data.frame(Replicate = 2, Line = "Aaeg-M", larvae = 100,
                   adults = 82, larval_deaths = 18,
                   male_adults = 82, Day = 14, Event = 0)

temporary_event <- rbind(temporary_event, dat, dat1)

# Add the censored values.
censored <- unique(ddply(temporary_event,
                         .(Replicate,Line,larvae,adults,
                           larval_deaths,male_adults),
                         summarise,
                         variable = Day,
                         Event = sum(Event),
                         Censored = (male_adults-Event))[,-7])
censored$variable <- max(data2$variable)

dat <- NULL
temporary <- NULL
for (i in 1:nrow(censored)) {
  N <- censored[i, "Censored"]
  dat <- data.frame(Replicate = rep(censored[i,"Replicate"],N),
                    Line = rep(censored[i,"Line"],N),
                    larvae = rep(censored[i,"larvae"],N),
                    adults = rep(censored[i,"adults"],N),
                    larval_deaths = rep(censored[i,"larval_deaths"],N),
                    male_adults = rep(censored[i,"male_adults"],N),
                    Day = rep(censored[i,"variable"],N),
                    Event = rep(0,N) )
```

```
  temporary <- rbind(temporary,dat )
}

data_surv <- rbind(temporary_event, temporary)
data_surv <- arrange(data_surv, Line,Replicate,desc(Event),Day)

SurvObj <- with(data_surv, Surv(Day, Event))
```

Model .

```
my.KMest <- survfit(SurvObj ~ Line , conf.int = 0.95, data = data_surv)
summary(my.KMest)
```

```
Call: survfit(formula = SurvObj ~ Line, data = data_surv, conf.int = 0.95)

                Line=Bra (WT)
 time n.risk n.event survival std.err lower 95% CI upper 95% CI
    1    791       5    0.994 0.00282        0.988        0.999
    2    786       2    0.991 0.00333        0.985        0.998
    5    784       4    0.986 0.00416        0.978        0.994
    6    780       1    0.985 0.00435        0.976        0.993
    7    779       2    0.982 0.00469        0.973        0.992
   10    777       2    0.980 0.00501        0.970        0.990
   12    775       1    0.979 0.00516        0.968        0.989
   13    774       2    0.976 0.00544        0.965        0.987

                Line=Aaeg-M
 time n.risk n.event survival std.err lower 95% CI upper 95% CI
    1    757       5    0.993 0.00294        0.988        0.999
    2    752       1    0.992 0.00322        0.986        0.998
    5    751       2    0.989 0.00372        0.982        0.997
    7    749       2    0.987 0.00415        0.979        0.995
    8    747       3    0.983 0.00472        0.974        0.992
   11    744       1    0.982 0.00490        0.972        0.991
   14    743       1    0.980 0.00507        0.970        0.990

                Line=Aaeg-CS
 time n.risk n.event survival std.err lower 95% CI upper 95% CI
    2    420       2    0.995 0.00336        0.989        1.000
    5    418       1    0.993 0.00411        0.985        1.000
   11    417       1    0.990 0.00474        0.981        1.000
   14    416       1    0.988 0.00529        0.978        0.999
```

```
df = data.frame(t = my.KMest$time,
                nb.rk = my.KMest$n.risk,
                line = c(rep(names(my.KMest$strata[1]),
                             my.KMest$strata[1]),
                         rep(names(my.KMest$strata[2]),
                             my.KMest$strata[2]),
                         rep(names(my.KMest$strata[3]),
                             my.KMest$strata[3]))
)
```

```r
# df$t[df$line == 'Line=MyriaF' & df$t==4] = 7
df$t[df$line == 'Line=Aaeg-CS' & df$t==5] = 7
# df$t[df$line == 'Line=MyriaF' & df$t==3] = 1
df$t[df$line == 'Line=Aaeg-CS' & df$t==2] = 1

# Test differences of survival at 7 and 14 days
data_aeg.sum =
  data_aeg %>%
  group_by(Line, Replicate) %>%
  dplyr::summarise(sur.7 = (male_adults -
                              (D1 + D2 + D3 + D4 + D5 + D6 + D7))/
                     male_adults,
            sur.14 = (male_adults - (D1 + D2 + D3 + D4 + D5 +
                                        D6 + D7 + D8 + D9 + D10 +
                                        D11 + D14 + D13 + D14))/
              male_adults)

mod.surv.7 = lm(data = data_aeg.sum,
   formula = sur.7 ~ Line)

summary(mod.surv.7)
```

```
Call:
lm(formula = sur.7 ~ Line, data = data_aeg.sum)

Residuals:
      Min        1Q    Median        3Q       Max
-0.030523 -0.004965  0.002688  0.008733  0.017097

Coefficients:
            Estimate Std. Error t value Pr(>|t|)
(Intercept) 0.982904   0.004902 200.529   <2e-16 ***
LineAaeg-M  0.004884   0.006932   0.705    0.491
LineAaeg-CS 0.009954   0.008490   1.172    0.257
---
Signif. codes:  0 '***' 0.001 '**' 0.01 '*' 0.05 '.' 0.1 ' ' 1

Residual standard error: 0.01386 on 17 degrees of freedom
Multiple R-squared:  0.07789,   Adjusted R-squared:  -0.0306
F-statistic: 0.7179 on 2 and 17 DF,  p-value: 0.502
```

```r
TukeyHSD(aov(mod.surv.7))
```

```
  Tukey multiple comparisons of means
    95% family-wise confidence level

Fit: aov(formula = mod.surv.7)

$Line
                        diff         lwr        upr     p adj
Aaeg-M-Bra (WT)  0.004884479 -0.01289814 0.02266710 0.7640413
```

```
Aaeg-CS-Bra (WT) 0.009953603 -0.01182557 0.03173277 0.4848501
Aaeg-CS-Aaeg-M   0.005069124 -0.01671005 0.02684830 0.8235245
```

```
mod.surv.14 = lm(data = data_aeg.sum,
   formula = sur.14 ~ Line)

summary(mod.surv.14)
```

```
Call:
lm(formula = sur.14 ~ Line, data = data_aeg.sum)

Residuals:
      Min        1Q    Median        3Q       Max 
-0.025193 -0.009310  0.002312  0.010522  0.020920 

Coefficients:
            Estimate Std. Error t value Pr(>|t|)    
(Intercept) 0.977574   0.005019 194.776   <2e-16 ***
LineAaeg-M  0.001505   0.007098   0.212    0.835    
LineAaeg-CS 0.008140   0.008693   0.936    0.362    
---
Signif. codes:  0 '***' 0.001 '**' 0.01 '*' 0.05 '.' 0.1 ' ' 1

Residual standard error: 0.0142 on 17 degrees of freedom
Multiple R-squared:  0.05089,   Adjusted R-squared:  -0.06077 
F-statistic: 0.4558 on 2 and 17 DF,  p-value: 0.6415
```

```
TukeyHSD(aov(mod.surv.14))
```

```
  Tukey multiple comparisons of means
    95% family-wise confidence level

Fit: aov(formula = mod.surv.14)

$Line
                        diff         lwr        upr     p adj
Aaeg-M-Bra (WT)  0.001505310 -0.01670328 0.01971390 0.9755453
Aaeg-CS-Bra (WT) 0.008139992 -0.01416088 0.03044087 0.6254566
Aaeg-CS-Aaeg-M   0.006634682 -0.01566619 0.02893556 0.7299193
```

Replication

```
# A tibble: 3 x 3
  Line         N     n
  <fct>    <int> <int>
1 Bra (WT)     8   791
2 Aaeg-M       8   755
3 Aaeg-CS      4   420
```

Model quality

```
check_model(mod.surv.7)
```

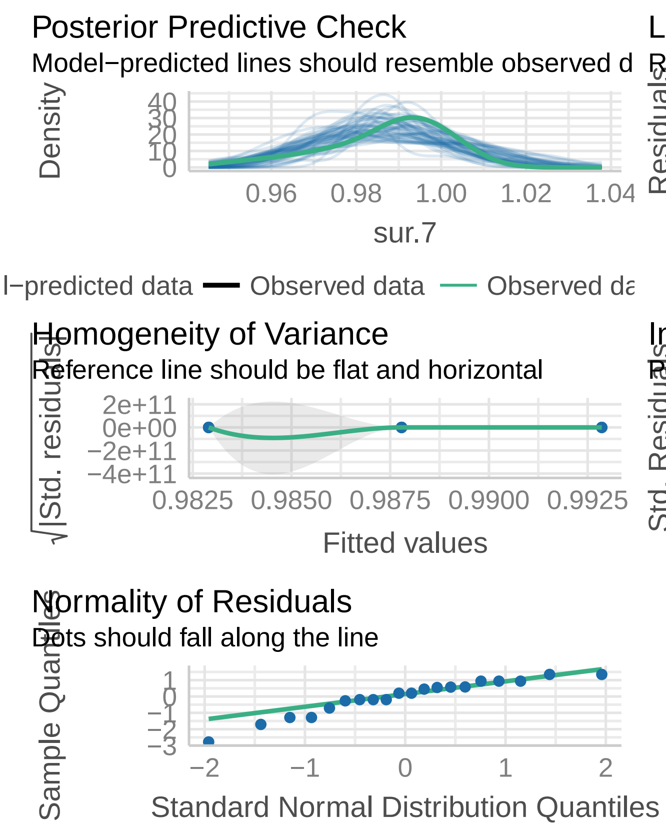


```
check_model(mod.surv.14)
```

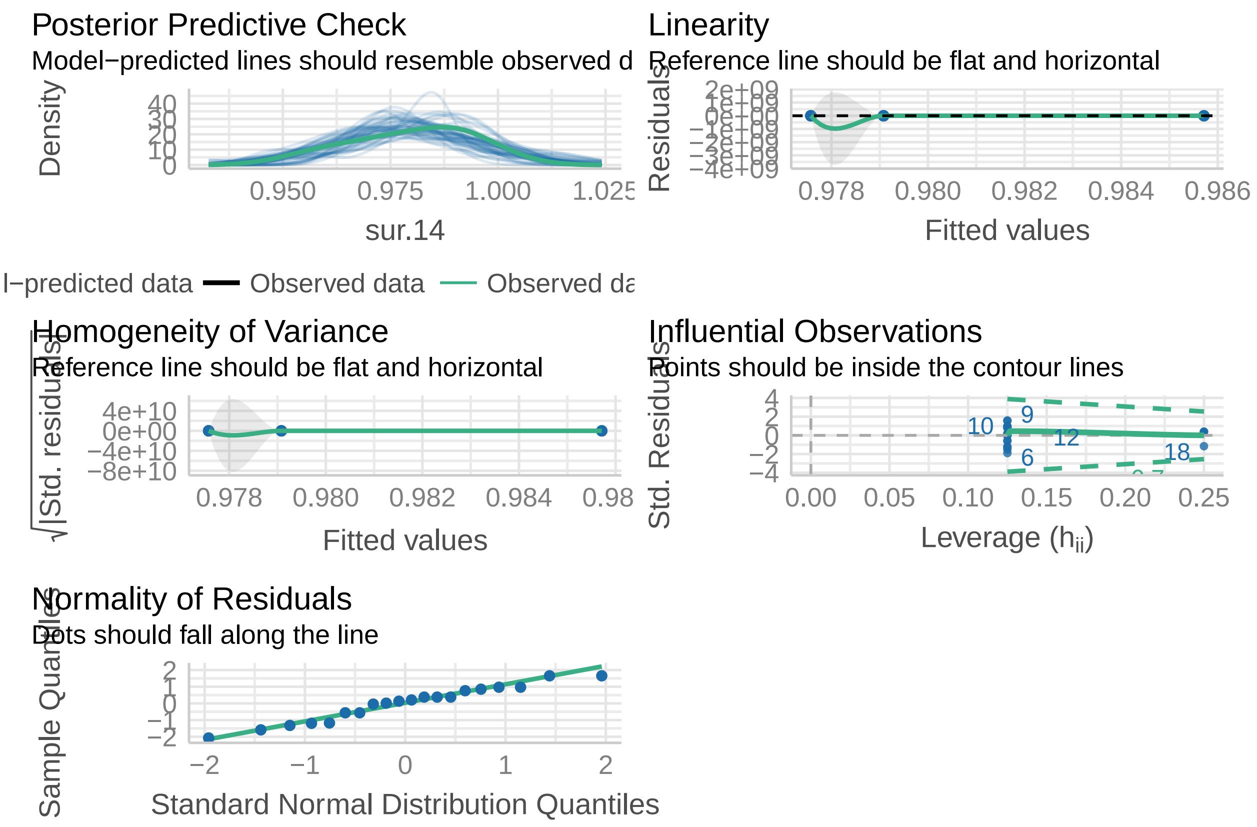

Plot .

```
ggsurv <-
  ggsurvplot(my.KMest,
             data = data_surv,
             conf.int = T,
             risk.table = T,
             pval = T,
             risk.table.height = 0.25,
             xlim = c(-0.5, 16.5))

line.color.aeg = c("#24ff24","#004949", "#006ddb")
names(line.color.aeg) = c('Line=Bra (WT)', 'Line=Aaeg-M', 'Line=Aaeg-CS')

p.aeg =
  ggsurv$plot +
  labs(x = 'Time (days)') +
  scale_y_continuous(limits =  c(.835, 1),
                     breaks = c(.84, .90, .95, 1),
                     labels = c(0, .90, .95,1)) +
  scale_x_continuous(breaks = c(0,7,14), limits = c(-1, 21)) +
  scale_color_manual(breaks = names(line.color.aeg),
                     values = line.color.aeg,
                     guide = 'none') +
  scale_fill_manual(breaks = names(line.color.aeg),
```

```
                    values = line.color.aeg,
                    labels = c("Bra (WT)", "Aaeg-M", "Aaeg-CS"),
                    name = "Line") +
  theme(axis.line.y = element_blank(),
        legend.position = 'right',
        panel.background = element_rect(fill = 'white')) +
  annotate(geom = 'segment', x = -Inf, xend = -Inf,
           y = -Inf, yend = Inf) +
  annotate(geom = 'segment', x = -Inf, xend = -Inf,
           y = .85, yend = .89, linetype = 'dashed', color = 'white') +
  annotate(geom = 'text',
           x = -.5, y = .91,
           label = "Number at risk",
           hjust = 0, size = 4) +
  annotate(geom = 'text',
           x = rep(0, 3), y =  seq(.841, .881, .02),
           label = rev(df$nb.rk[df$t == 1]),
           color = rev(c("#24ff24","#004949", "#006ddb")),
           size = 4) +
  annotate(geom = 'text',
           x = rep(7, 3), y =  seq(.841, .881, .02),
           label = rev(df$nb.rk[df$t == 7]),
           color = rev(c("#24ff24","#004949", "#006ddb")),
           size = 4) +
  annotate(geom = 'text',
           x = rep(14, 3), y =  seq(.841, .881, .02),
           label = rev(df$nb.rk[df$t == 14]),
           color = rev(c("#24ff24","#004949", "#006ddb")),
           size = 4) +
  annotate(geom = 'text', x = 15.5, y = .87, label = 'n.s', size = 4) +
  annotate(geom = "line", x = rep(14.85,2), y = c(.86,.88)) +
  annotate(geom = 'text', x = 16.7, y = .86, label = 'n.s', size = 4) +
  annotate(geom = "line", x = rep(16.1,2), y = c(.84,.88))

p.aeg
```

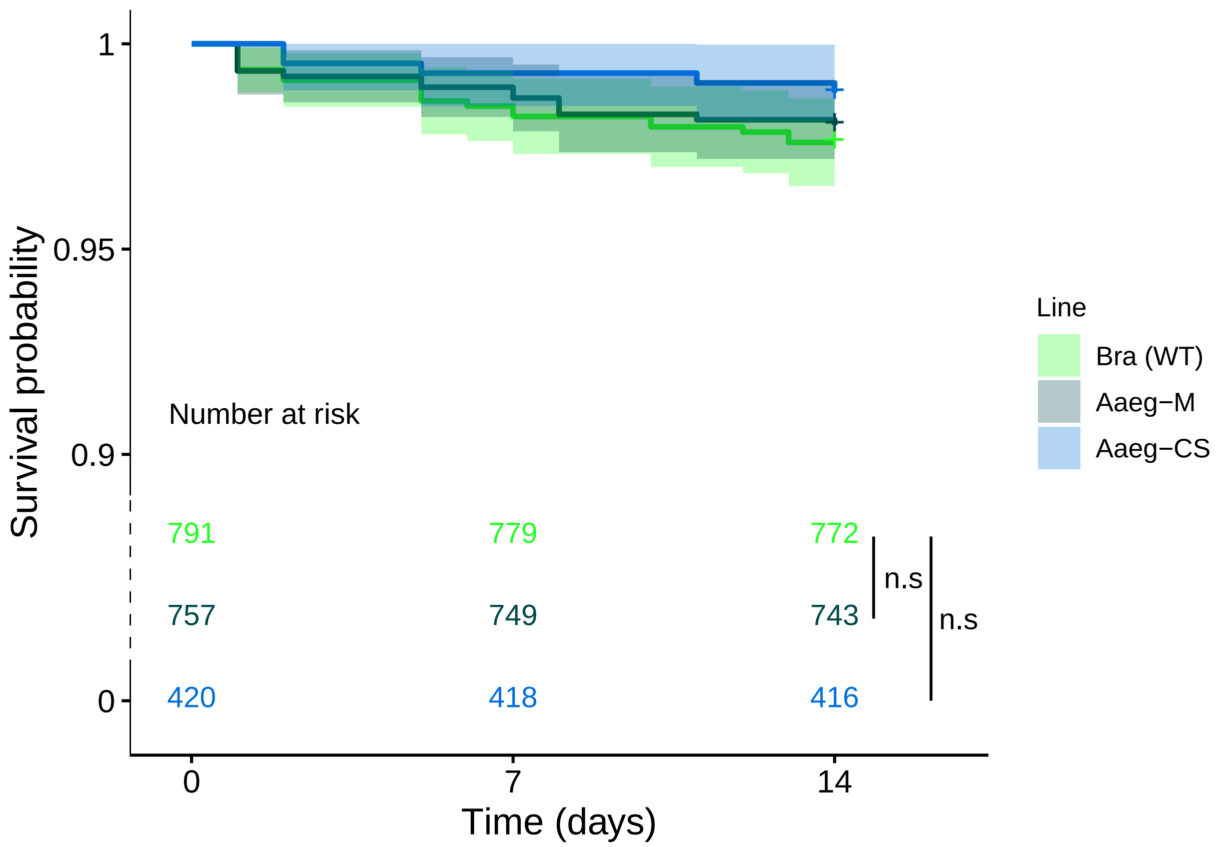

Survival probability
1
0.95
0.9
0
Number at risk
791
779
772
757
749
743
420
418
416
n.s
n.s
0
7
14
Time (days)
Line
Bra (WT)
Aaeg−M
Aaeg−CS

Ae. albopictus

Data .

```
data_albo <- read.table("data/Survival_albo.txt", h = T)
data_albo$Line[data_albo$Line=='BiA-(WT)'] = 'BiA (WT)'

data_albo |> dplyr::select(-(3:5))
```

```
   Replicate     Line male_adults D1 D2 D3 D4 D5 D6 D7 D8 D9 D10 D11 D12 D13
1          1 BiA (WT)          56  2  1  0  1  0  0  0  0  0   0   0   0   0
2          2 BiA (WT)          96  1  1  0  0  0  0  0  0  0   0   0   0   1
3          3 BiA (WT)          91  0  0  0  0  0  0  0  0  0   0   0   0   0
4          4 BiA (WT)          76  1  0  0  0  0  0  0  0  1   0   0   0   0
5          1    Aal-M          88  1  1  0  2  0  0  0  0  0   0   0   1   0
6          2    Aal-M          79  1  1  0  0  0  1  0  0  0   1   0   0   0
7          3    Aal-M          80  1  0  0  0  0  0  0  1  0   0   0   1   0
8          4    Aal-M          73  1  0  0  0  0  0  0  0  0   0   0   0   0
9          1   Aal-CS          71  0  0  0  0  1  0  1  0  0   0   0   0   2
10         2   Aal-CS          73  0  0  0  2  0  0  0  0  0   0   1   0   1
11         3   Aal-CS          61  0  0  0  0  0  0  0  0  0   0   0   0   1
12         4   Aal-CS          69  0  0  0  0  0  0  0  1  0   0   0   0   0
   D14
1    0
2    0
3    0
4    0
5    0
6    0
7    0
8    0
9    0
10   0
11   0
12   0
```

```
data1 <- melt(data_albo,
              .(Replicate,Line, larvae, adults, larval_deaths, male_adults))
#Then transform days in numbers
data1$variable <- as.numeric(str_replace(data1$variable, "D",""))
#Sort by Line, Replicate and variable.
data2 <- arrange(data1, Line, Replicate, variable )
#We now separate the data set in two parts: individuals that has experienced an event(death) and censor

event <- subset(data2, value>0)

#We will now work of the dataset with individuals experiencing an event.

dat <- NULL
temporary_event <- NULL
for (i in 1:nrow(event)) {
  N <- event[i, "value"]
  dat <- data.frame(Replicate=rep(event[i,"Replicate"],N),
```

```
                     Line=rep(event[i,"Line"],N),
                     larvae=rep(event[i,"larvae"],N),
                     adults=rep(event[i,"adults"],N),
                     larval_deaths=rep(event[i,"larval_deaths"],N),
                     male_adults=rep(event[i,"male_adults"],N),
                     Day=rep(event[i,"variable"],N),
                     Event=rep(1,N))

  temporary_event  <- rbind(temporary_event ,dat )
}

temporary_event
```

```
   Replicate     Line larvae adults larval_deaths male_adults Day Event
1          1   Aal-CS    100     71            29          71   5     1
2          1   Aal-CS    100     71            29          71   7     1
3          1   Aal-CS    100     71            29          71  13     1
4          1   Aal-CS    100     71            29          71  13     1
5          2   Aal-CS    100     73            27          73   4     1
6          2   Aal-CS    100     73            27          73   4     1
7          2   Aal-CS    100     73            27          73  11     1
8          2   Aal-CS    100     73            27          73  13     1
9          3   Aal-CS    100     61            39          61  13     1
10         4   Aal-CS    100     69            31          69   8     1
11         1    Aal-M    100     88            12          88   1     1
12         1    Aal-M    100     88            12          88   2     1
13         1    Aal-M    100     88            12          88   4     1
14         1    Aal-M    100     88            12          88   4     1
15         1    Aal-M    100     88            12          88  12     1
16         2    Aal-M    100     79            21          79   1     1
17         2    Aal-M    100     79            21          79   2     1
18         2    Aal-M    100     79            21          79   6     1
19         2    Aal-M    100     79            21          79  10     1
20         3    Aal-M    100     80            20          80   1     1
21         3    Aal-M    100     80            20          80   8     1
22         3    Aal-M    100     80            20          80  12     1
23         4    Aal-M    100     77            23          73   1     1
24         1 BiA (WT)    200    103            97          56   1     1
25         1 BiA (WT)    200    103            97          56   1     1
26         1 BiA (WT)    200    103            97          56   2     1
27         1 BiA (WT)    200    103            97          56   4     1
28         2 BiA (WT)    200    141            59          96   1     1
29         2 BiA (WT)    200    141            59          96   2     1
30         2 BiA (WT)    200    141            59          96  13     1
31         4 BiA (WT)    200    130            70          76   1     1
32         4 BiA (WT)    200    130            70          76   9     1
```

```
# here is or transformed dataset with one
# row for each time we have an event.

# BIA rep3 has no event over the course of
# the study, we add it manually so that it would appear.
dat <- data.frame(Replicate=3, Line="BiA (WT)",
```

```r
                   larvae=200,  adults=129, larval_deaths=71,
                   male_adults=91, Day=14, Event=0)

temporary_event <- rbind(temporary_event, dat)

#We now add the censored values.

censored <- unique(ddply(temporary_event,
                          .(Replicate,Line,larvae,adults,larval_deaths,male_adults),
                          summarise,
                          variable=Day,
                          Event=sum(Event),
                          Censored=(male_adults-Event))[,-7])
censored$variable <- max(data2$variable)

dat <- NULL
temporary <- NULL
for (i in 1:nrow(censored)) {
  N <- censored[i, "Censored"]
  dat <- data.frame(Replicate=rep(censored[i,"Replicate"],N) ,
                    Line=rep(censored[i,"Line"],N) ,
                    larvae=rep(censored[i,"larvae"],N) ,
                    adults=rep(censored[i,"adults"],N) ,
                    larval_deaths=rep(censored[i,"larval_deaths"],N) ,
                    male_adults=rep(censored[i,"male_adults"],N),
                    Day=rep(censored[i,"variable"],N),
                    Event=rep(0,N) )

  temporary <- rbind(temporary,dat )
}

#Bind the final dataset
data_surv <- rbind(temporary_event, temporary)
data_surv <- arrange(data_surv, Line,Replicate,desc(Event),Day)

SurvObj <- with(data_surv, Surv(Day, Event))
```

Model .

```r
my.KMest <- survfit(SurvObj ~ Line , conf.int = 0.95, data = data_surv)
summary(my.KMest)
```

```
Call: survfit(formula = SurvObj ~ Line, data = data_surv, conf.int = 0.95)

                Line=Aal-CS
 time n.risk n.event survival std.err lower 95% CI upper 95% CI
    4    274       2    0.993 0.00514        0.983        1.000
    5    272       1    0.989 0.00629        0.977        1.000
    7    271       1    0.985 0.00725        0.971        1.000
    8    270       1    0.982 0.00809        0.966        0.998
   11    269       1    0.978 0.00884        0.961        0.996
   13    268       4    0.964 0.01133        0.942        0.986
```

```
                Line=Aal-M
 time n.risk n.event survival std.err lower 95% CI upper 95% CI
    1    320       4    0.988 0.00621        0.975        1.000
    2    316       2    0.981 0.00758        0.967        0.996
    4    314       2    0.975 0.00873        0.958        0.992
    6    312       1    0.972 0.00924        0.954        0.990
    8    311       1    0.969 0.00973        0.950        0.988
   10    310       1    0.966 0.01018        0.946        0.986
   12    309       2    0.959 0.01104        0.938        0.981

                Line=BiA (WT)
 time n.risk n.event survival std.err lower 95% CI upper 95% CI
    1    320       4    0.988 0.00621        0.975        1.000
    2    316       2    0.981 0.00758        0.967        0.996
    4    314       1    0.978 0.00818        0.962        0.994
    9    313       1    0.975 0.00873        0.958        0.992
   13    312       1    0.972 0.00924        0.954        0.990
```

```r
df = data.frame(t = my.KMest$time,
                nb.rk = my.KMest$n.risk,
                line = c(rep(names(my.KMest$strata[1]),
                             my.KMest$strata[1]),
                         rep(names(my.KMest$strata[2]),
                             my.KMest$strata[2]),
                         rep(names(my.KMest$strata[3]),
                             my.KMest$strata[3]))
)

df$t[df$line == 'Line=Aal-M' & df$t==6] = 7
df$t[df$line == 'Line=BiA (WT)' & df$t==4] = 7
df$t[df$line == 'Line=Aal-CS' & df$t==4] = 1

df$line = factor(df$line, levels = c('Line=BiA (WT)','Line=Aal-M','Line=Aal-CS'))
df = df[order(df$line),]

data_albo.sum =
  data_albo %>%
  group_by(Line, Replicate) %>%
  dplyr::summarise(sur.7 = (male_adults -
                              (D1 + D2 + D3 + D4 + D5 + D6 + D7))/
                     male_adults,
            sur.14 = (male_adults - (D1 + D2 + D3 + D4 + D5 +
                                       D6 + D7 + D8 + D9 + D10 +
                                       D11 + D14 + D13 + D14))/
              male_adults)

data_albo.sum$Line = data_albo.sum$Line %>%
  factor(., levels = c('BiA (WT)','Aal-M', 'Aal-CS'))

mod.surv.7 = lm(data = data_albo.sum,
   formula = sur.7 ~ Line)

summary(mod.surv.7)
```

```
Call:
lm(formula = sur.7 ~ Line, data = data_albo.sum)

Residuals:
      Min        1Q    Median        3Q       Max
-0.045074 -0.013699  0.009359  0.013892  0.026355

Coefficients:
             Estimate Std. Error t value Pr(>|t|)
(Intercept)  0.973645   0.011243  86.600 1.85e-14 ***
LineAal-M   -0.001052   0.015900  -0.066    0.949
LineAal-CS   0.012463   0.015900   0.784    0.453
---
Signif. codes:  0 '***' 0.001 '**' 0.01 '*' 0.05 '.' 0.1 ' ' 1

Residual standard error: 0.02249 on 9 degrees of freedom
Multiple R-squared:  0.09038,   Adjusted R-squared:  -0.1118
F-statistic: 0.4471 on 2 and 9 DF,  p-value: 0.6529
```

```
TukeyHSD(aov(mod.surv.7))
```

```
  Tukey multiple comparisons of means
    95% family-wise confidence level

Fit: aov(formula = mod.surv.7)

$Line
                        diff         lwr        upr     p adj
Aal-M-BiA (WT)  -0.001052015 -0.04544497 0.04334094 0.9975900
Aal-CS-BiA (WT)  0.012463381 -0.03192957 0.05685634 0.7216963
Aal-CS-Aal-M     0.013515396 -0.03087756 0.05790835 0.6831225
```

```
mod.surv.14 = lm(data = data_albo.sum,
   formula = sur.14 ~ Line)

summary(mod.surv.14)
```

```
Call:
lm(formula = sur.14 ~ Line, data = data_albo.sum)

Residuals:
      Min        1Q    Median        3Q       Max
-0.039180 -0.017525  0.003466  0.019333  0.032249

Coefficients:
             Estimate Std. Error t value Pr(>|t|)
(Intercept)  0.967751   0.011931  81.111 3.33e-14 ***
LineAal-M   -0.001448   0.016873  -0.086    0.933
LineAal-CS  -0.003256   0.016873  -0.193    0.851
---
Signif. codes:  0 '***' 0.001 '**' 0.01 '*' 0.05 '.' 0.1 ' ' 1
```

```
Residual standard error: 0.02386 on 9 degrees of freedom
Multiple R-squared:  0.004137,  Adjusted R-squared:  -0.2172
F-statistic: 0.0187 on 2 and 9 DF,  p-value: 0.9815
```

```
TukeyHSD(aov(mod.surv.14))
```

```
  Tukey multiple comparisons of means
    95% family-wise confidence level

Fit: aov(formula = mod.surv.14)

$Line
                        diff         lwr        upr     p adj
Aal-M-BiA (WT)  -0.001447932 -0.04855825 0.04566239 0.9959503
Aal-CS-BiA (WT) -0.003256096 -0.05036641 0.04385422 0.9797262
Aal-CS-Aal-M    -0.001808164 -0.04891848 0.04530215 0.9936934
```

Replication

```
# A tibble: 3 x 3
  Line         N     n
  <chr>    <int> <int>
1 Aal-CS       4   274
2 Aal-M        4   320
3 BiA (WT)     4   319
```

Model quality

```
check_model(mod.surv.7)
```

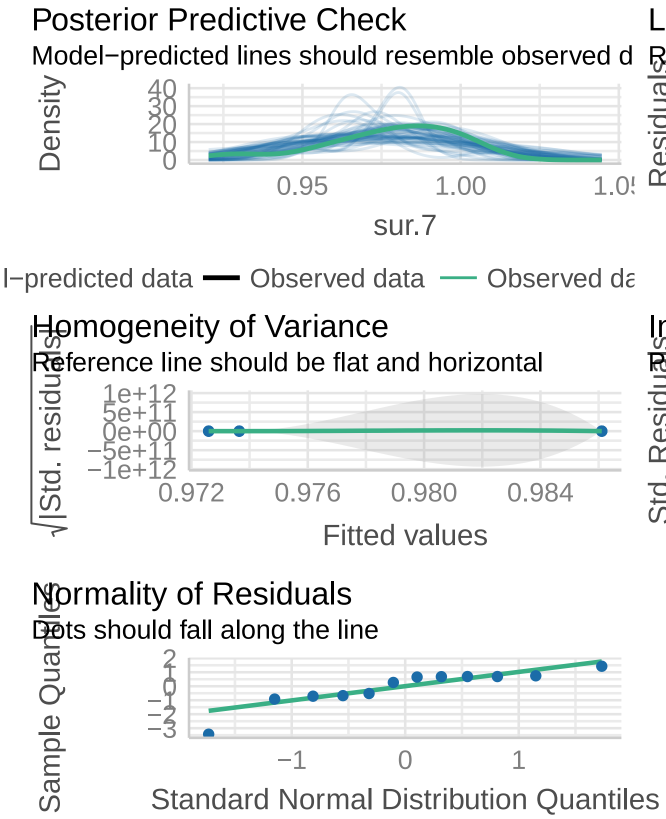


```
check_model(mod.surv.14)
```

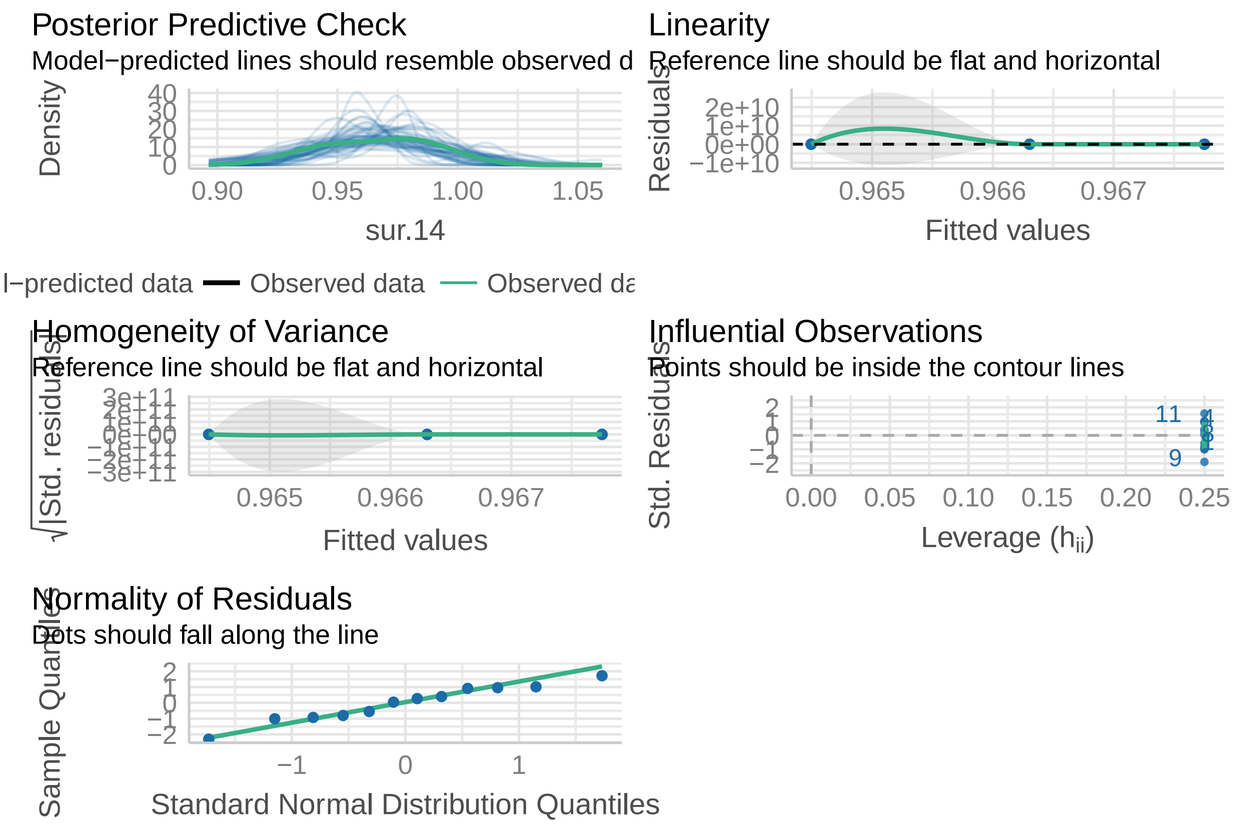

Plot .

```
ggsurv <-
  ggsurvplot(my.KMest,
             data = data_surv,
             conf.int = T,
             risk.table = T,
             pval = T,
             risk.table.height = 0.25,
              xlim = c(-0.5, 16.5))

line.color.alb = c("#24ff24","#004949", "#006ddb")
names(line.color.alb) = c('Line=BiA (WT)', 'Line=Aal-M', 'Line=Aal-CS')

p.albo =
  ggsurv$plot +
  labs(x = 'Time (days)') +
  scale_y_continuous(limits =  c(.835, 1),
                     breaks = c(.84, .90, .95, 1),
                     labels = c(0, .90, .95,1)) +
  scale_x_continuous(breaks = c(0,7,14)) +
  scale_color_manual(breaks = names(line.color.alb),
                     values = line.color.alb,
                     guide = 'none') +
  scale_fill_manual(breaks = names(line.color.alb),
```

```
                    values = line.color.alb,
                    labels = c("BiA (WT)", "Aal-M", "Aal-CS"),
                    name = "Line: ") +
  theme(axis.line.y = element_blank(),
        legend.position = 'right',
        panel.background = element_rect(fill = 'grey')) +
  annotate(geom = 'segment', x = -Inf, xend = -Inf,
           y = -Inf, yend = Inf) +
  annotate(geom = 'segment', x = -Inf, xend = -Inf,
           y = .85, yend = .89, linetype = 'dashed', color = 'white') +
  annotate(geom = 'text',
           x = -.5, y = .895,
           label = "Number at risk",
           hjust = 0, size = 4) +
  annotate(geom = 'text',
           x = rep(0, 3), y =  seq(.841, .881, .02),
           label = rev(df$nb.rk[df$t == 1]),
           color = rev(c("#24ff24","#004949", "#006ddb")),
           size = 4) +
  annotate(geom = 'text',
           x = rep(7, 3), y =  seq(.841, .881, .02),
           label = rev(df$nb.rk[df$t == 7]),
           color = rev(c("#24ff24","#004949", "#006ddb")),
           size = 4) +
  annotate(geom = 'text',
           x = rep(14, 3), y =  seq(.841, .881, .02),
           label = rev(df$nb.rk[df$t == 14]),
           color = rev(c("#24ff24","#004949", "#006ddb")),
           size = 4) +
  annotate(geom = 'text', x = 15.5, y = .87, label = 'n.s', size = 4) +
  annotate(geom = "line", x = rep(14.85,2), y = c(.86,.88)) +
  annotate(geom = 'text', x = 16.7, y = .86, label = 'n.s', size = 4) +
  annotate(geom = "line", x = rep(16.1,2), y = c(.84,.88))

p.albo
```

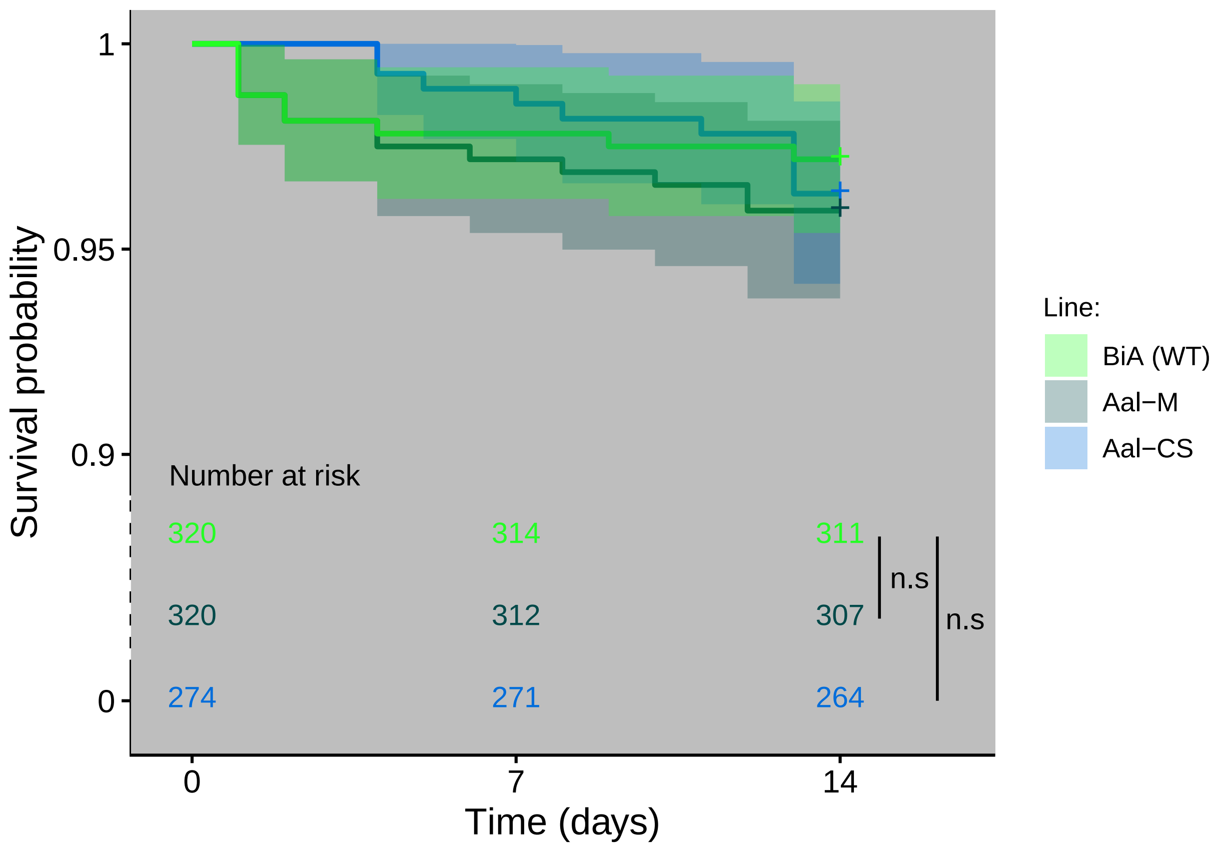

Survival probability
1
0.95
0.9
0
Number at risk
320
314
311
320
312
307
274
271
264
n.s
n.s
0
7
14
Time (days)
Line:
BiA (WT)
Aal−M
Aal−CS

Figure 5

Data

```
speed.aeg = read.csv('data/Speed-test_aeg.csv', sep = ';') %>%
  mutate(recovery = 100*recovered_males/total_males) %>%
  mutate(contamination = 100*contamination/total_males)

speed.albo = read.csv('data/Speed-test_albo.csv', sep = ';') %>%
  mutate(recovery = 100*recovered_males/total_males) %>%
  mutate(contamination = 100*contamination/total_males)

head(speed.aeg)
```

```
  Replicate Volume Larval_concentration Mean_speed total_males recovered_males
1         1    500                   20          6    3924.536            3619
2         2    500                   20          6    3898.820            3578
3         3    500                   20          6    3829.696            3556
4         1    250                   40         12    3672.180            3328
5         2    250                   40         12    3366.765            2927
6         3    250                   40         12    3408.405            3037
  contamination recovery
1             0 92.21472
2             0 91.77136
3             0 92.85332
4             0 90.62737
5             0 86.93805
6             0 89.10326
```

Model

Recovery

.

```
speed.lm = lm(formula = recovery~Mean_speed,
              data = speed.aeg)

speed.poly2 = lm(formula = recovery~poly(Mean_speed, degree = 2),
              data = speed.aeg)

speed.poly3 = lm(formula = recovery~poly(Mean_speed, degree = 3),
              data = speed.aeg)

speed.poly4 = lm(formula = recovery~poly(Mean_speed, degree = 4),
              data = speed.aeg)

speed.log = lm(formula = recovery~log(Mean_speed),
              data = speed.aeg)

compare_performance(speed.lm, speed.log,
```

```
                      speed.poly2, speed.poly3,
                      speed.poly4)
```

```
# Comparison of Model Performance Indices

Name        | Model | AIC (weights) | AICc (weights) | BIC (weights) |    R2 | R2 (adj.) |  RMSE | Sigma
---------------------------------------------------------------------------------------------------------
speed.lm    |    lm | 107.1 (<.001) |  109.3 (<.001) | 109.2 (<.001) | 0.917 |     0.911 | 7.034 | 7.55
speed.log   |    lm | 103.2 (<.001) |  105.3 (<.001) | 105.3 (<.001) | 0.936 |     0.932 | 6.169 | 6.62
speed.poly2 |    lm |  82.9 (0.409) |   86.9 (0.773) |  85.8 (0.518) | 0.986 |     0.983 | 2.941 | 3.28
speed.poly3 |    lm |  82.8 (0.432) |   89.5 (0.215) |  86.4 (0.383) | 0.987 |     0.984 | 2.742 | 3.20
speed.poly4 |    lm |  84.8 (0.159) |   95.3 (0.012) |  89.1 (0.099) | 0.987 |     0.982 | 2.742 | 3.35
```

Best model:

```
summary(speed.poly2)
```

```
Call:
lm(formula = recovery ~ poly(Mean_speed, degree = 2), data = speed.aeg)

Residuals:
    Min      1Q  Median      3Q     Max 
-6.2202 -1.0021  0.2709  1.1871  6.7801 

Coefficients:
                              Estimate Std. Error t value Pr(>|t|)    
(Intercept)                     65.498      0.849  77.144  < 2e-16 ***
poly(Mean_speed, degree = 2)1  -90.774      3.288 -27.605 3.15e-12 ***
poly(Mean_speed, degree = 2)2   24.746      3.288   7.526 6.99e-06 ***
---
Signif. codes:  0 '***' 0.001 '**' 0.01 '*' 0.05 '.' 0.1 ' ' 1

Residual standard error: 3.288 on 12 degrees of freedom
Multiple R-squared:  0.9856,    Adjusted R-squared:  0.9831 
F-statistic: 409.3 on 2 and 12 DF,  p-value: 9.089e-12
```

Contamination

.

```
conta.lm = lm(formula = contamination~Mean_speed,
              data = speed.aeg)

conta.poly2 = lm(formula = contamination~poly(Mean_speed, degree = 2),
              data = speed.aeg)

conta.poly3 = lm(formula = contamination~poly(Mean_speed, degree = 3),
              data = speed.aeg)

conta.poly4 = lm(formula = contamination~poly(Mean_speed, degree = 4),
```

```r
              data = speed.aeg)

conta.log = lm(formula = contamination~log(Mean_speed),
              data = speed.aeg)

compare_performance(conta.lm,
                    conta.log,
                    conta.poly2,
                    conta.poly3,
                    conta.poly4)
```

```
# Comparison of Model Performance Indices

Name        | Model | AIC (weights) | AICc (weights) | BIC (weights) |    R2 | R2 (adj.) |  RMSE | Sigma
-----------------------------------------------------------------------------------------------------------
conta.lm    |    lm | -17.1 (0.138) |  -14.9 (0.354) | -15.0 (0.207) | 0.626 |     0.597 | 0.112 | 0.120
conta.log   |    lm |  -8.2 (0.002) |   -6.1 (0.004) |  -6.1 (0.002) | 0.326 |     0.274 | 0.150 | 0.16[illegible]
conta.poly2 |    lm | -19.9 (0.561) |  -15.9 (0.579) | -17.1 (0.588) | 0.729 |     0.683 | 0.096 | 0.107
conta.poly3 |    lm | -18.0 (0.219) |  -11.3 (0.060) | -14.5 (0.161) | 0.731 |     0.657 | 0.095 | 0.11[illegible]
conta.poly4 |    lm | -16.0 (0.081) |   -5.5 (0.003) | -11.8 (0.042) | 0.731 |     0.623 | 0.095 | 0.117
```

Best model

```r
summary(conta.lm)
```

```

Call:
lm(formula = contamination ~ Mean_speed, data = speed.aeg)

Residuals:
     Min       1Q   Median       3Q      Max 
-0.25076 -0.06549  0.03905  0.04709  0.24102 

Coefficients:
              Estimate Std. Error t value Pr(>|t|)    
(Intercept) -0.0551361  0.0422440  -1.305 0.214460    
Mean_speed   0.0013403  0.0002872   4.667 0.000441 ***
---
Signif. codes:  0 '***' 0.001 '**' 0.01 '*' 0.05 '.' 0.1 ' ' 1

Residual standard error: 0.1204 on 13 degrees of freedom
Multiple R-squared:  0.6262,    Adjusted R-squared:  0.5975 
F-statistic: 21.78 on 1 and 13 DF,  p-value: 0.0004409
```

Plot

.

```r
pred.speed = ggpredict(speed.poly2) %>%
  data.frame()
pred.conta = ggpredict(conta.lm) %>%
```

```
  data.frame()

reco.plot =
  ggplot(data = NULL) +
  annotate(geom = 'line', x = c(60, 60), y = c(-Inf,70),
           lty =2, color = 'gray', size = 1 ) +
  annotate(geom = 'line', x = c(-Inf, 60), y = c(70,70),
           lty =2, color = 'gray', size = 1 ) +
  geom_ribbon(data = pred.speed,
              aes(x = Mean_speed.x,
                  ymin = Mean_speed.conf.low,
                  ymax = Mean_speed.conf.high),
              fill = "grey", alpha=.3) +
  geom_line(data= pred.speed,
            aes(Mean_speed.x,
                Mean_speed.predicted),
            col = "black") +
  geom_ribbon(data = pred.conta,
              aes(x = Mean_speed.x,
                  ymin = Mean_speed.conf.low,
                  ymax = Mean_speed.conf.high),
              fill = "red", alpha=.3) +
  geom_line(data= pred.conta,
            aes(x = Mean_speed.x,
                y = Mean_speed.predicted),
            col="red") +
  geom_point(data = speed.aeg,
             aes(x = Mean_speed,
                 y = recovery,
                 col = 'Recovery', pch = "Ae. Aegypti")) +
  geom_point(data = speed.aeg,
             aes(x = Mean_speed,
                 y = contamination,
                 col = 'Contamination', pch = "Ae. Aegypti")) +
  geom_point(data = speed.albo,
             aes(x = Mean_speed,
                 y = recovery,
                 col = 'Recovery', pch = "Ae. Albopictus")) +
  geom_point(data = speed.albo,
             aes(x = Mean_speed,
                 y = contamination,
                 col = 'Contamination', pch = "Ae. Albopictus")) +
  annotate(geom='text', x = -27, y =70, label = bquote(italic('70')), color = 'gray') +
  annotate(geom='text', x = 275, y =50, label = bquote(R^2 ~ '= 98.3%'), color = 'black') +
  annotate(geom='text', x = 275, y =10, label = bquote(R^2 ~ '= 55.6%'), color = 'red') +
  labs(x = 'Sorting speed (larvae/sec) \n [larval concentration (larvae/mL)]',
       y = 'Percentage of recovery\n and contamination (%)',
       color = "Measurement" ,
       pch = "Species") +
  scale_y_continuous(breaks = seq(0,100,25)) +
  scale_x_continuous(breaks = seq(0, 300, 60),
                     labels = c("0 [0]", '60 [200]', '120 [400]',
                                '180 [600]', '240 [800]', '300 [1000]')) +
```

```
  scale_shape_manual(labels = c(bquote(italic("Ae. aegypti")),
                                bquote(italic("Ae. albopictus"))),
                     values = c(16, 17)) +
  scale_color_manual(labels = c("Recovery",
                                "Contamination"),
                     values = c(Recovery = "black", Contamination = "red")) +
  coord_cartesian(xlim = c(0,300), ylim = c(-5,100), clip = 'off') +
  theme_classic() +
  theme(text = element_text(size = 15),
        legend.position = 'right')

reco.plot
```

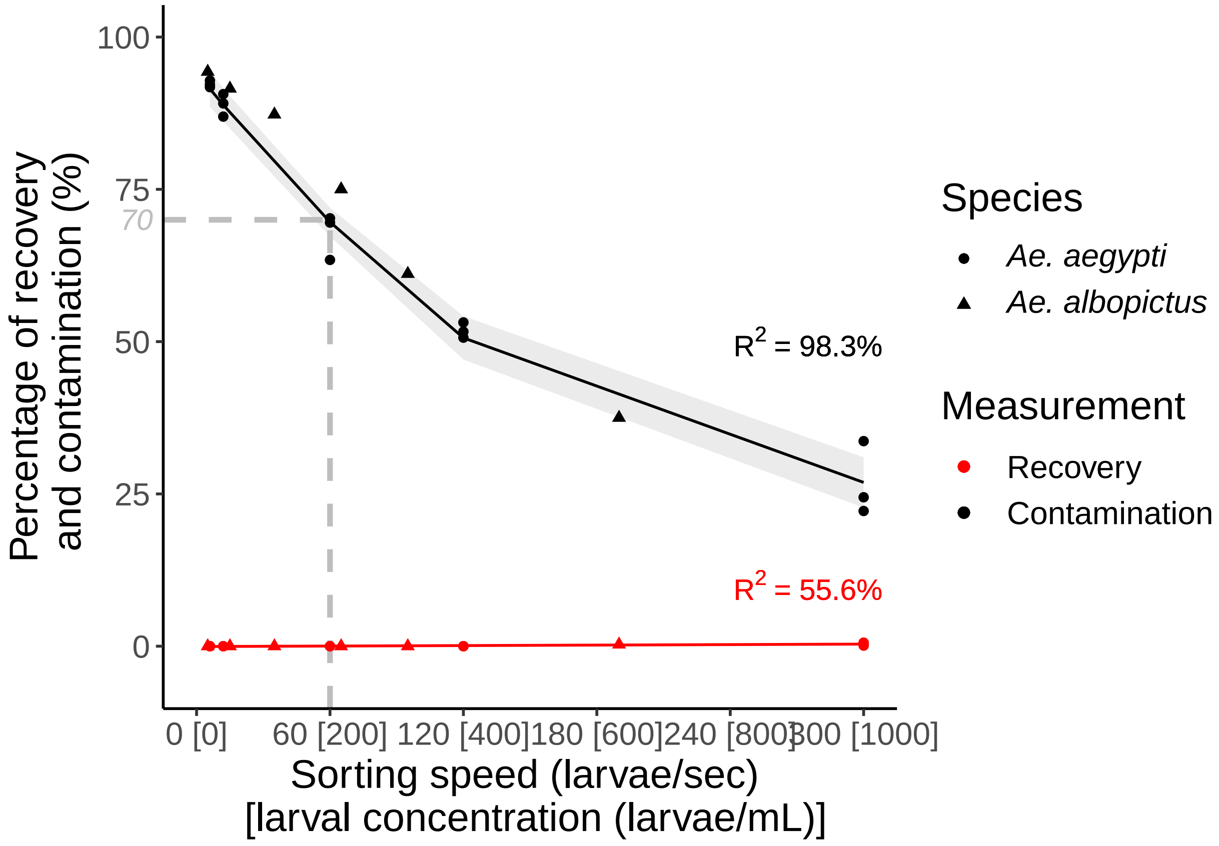

Supplement: Supplementary file 5 — Supplementary Data 2 [file 42003_2023_5030_MOESM5_ESM.pdf]
