# Supplementary material for: Combining two genetic sexing strains allows sorting of non-transgenic males for Aedes genetic control
Source: Commun Biol. 2023 Jun 16;6:646. doi: 10.1038/s42003-023-05030-7 (PMC10275924; doi:10.1038/s42003-023-05030-7)
Supplement: Supplementary file 6 — Supplementary Data 3 [file 42003_2023_5030_MOESM6_ESM.pdf]

Supplementary Data 3 - Production costs and initial parameters

Lutrat et al. 2023

Contents

Mass rearing processes 2

Size-based sorting . . . 2

GSS . . . 3

GSS-CS . . . 4

Simulation outputs 5

Insect production . . . 5

Facility area . . . 5

Workload (both facilities) . . . 6

Water requirements . . . 6

Construction budget . . . 6

Equipment budget . . . 7

Diet and consumables yearly cost . . . 8

Estimate of the total yearly cost for construction, equipment, diet and consumables . . . 8

Initial parameters 9

Mass rearing processes

Size-based sorting

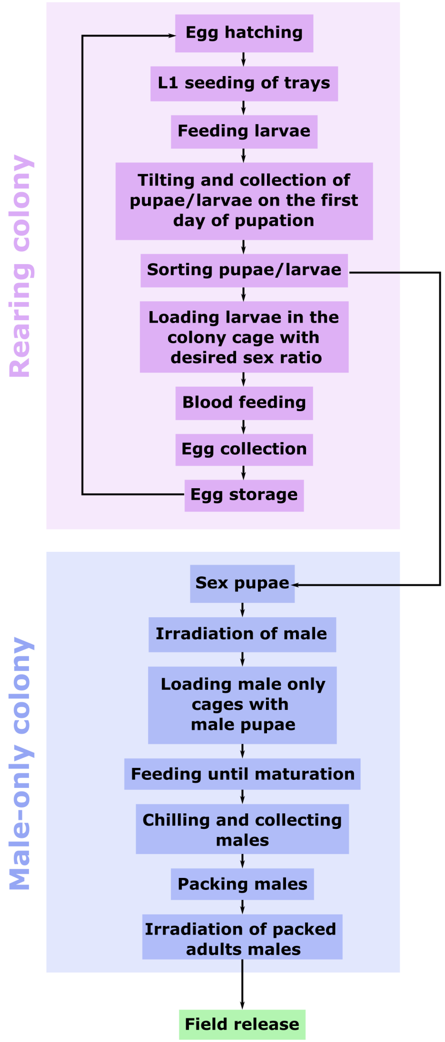

GSS

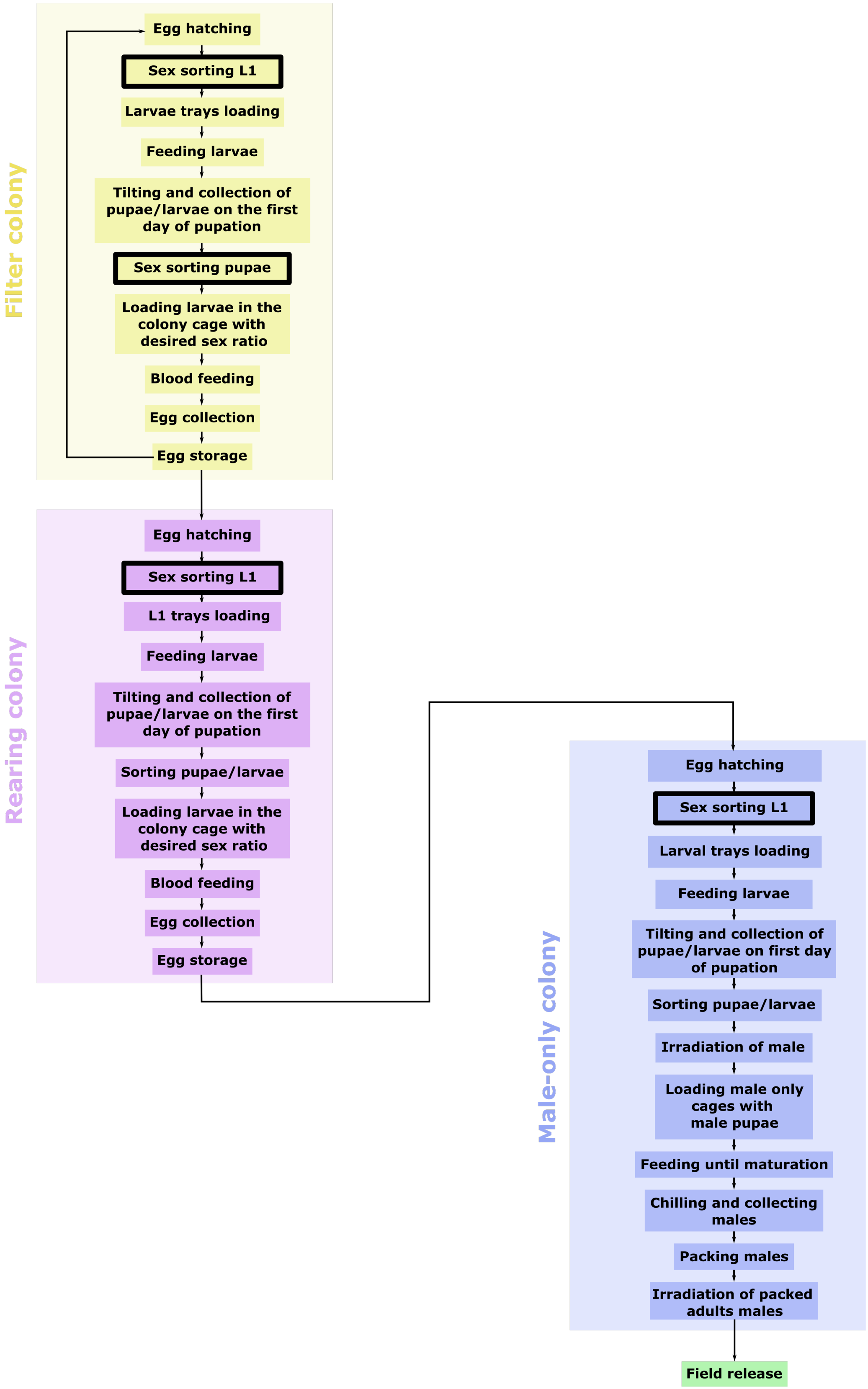

Filter colony
Egg hatching
Sex sorting L1
Larvae trays loading
Feeding larvae
Tilting and collection of pupae/larvae on the first day of pupation
Sex sorting pupae
Loading larvae in the colony cage with desired sex ratio
Blood feeding
Egg collection
Egg storage
Rearing colony
Egg hatching
Sex sorting L1
L1 trays loading
Feeding larvae
Tilting and collection of pupae/larvae on the first day of pupation
Sorting pupae/larvae
Loading larvae in the colony cage with desired sex ratio
Blood feeding
Egg collection
Egg storage
Male-only colony
Egg hatching
Sex sorting L1
Larval trays loading
Feeding larvae
Tilting and collection of pupae/larvae on first day of pupation
Sorting pupae/larvae
Irradiation of male
Loading male only cages with male pupae
Feeding until maturation
Chilling and collecting males
Packing males
Irradiation of packed adults males
Field release

GSS-CS

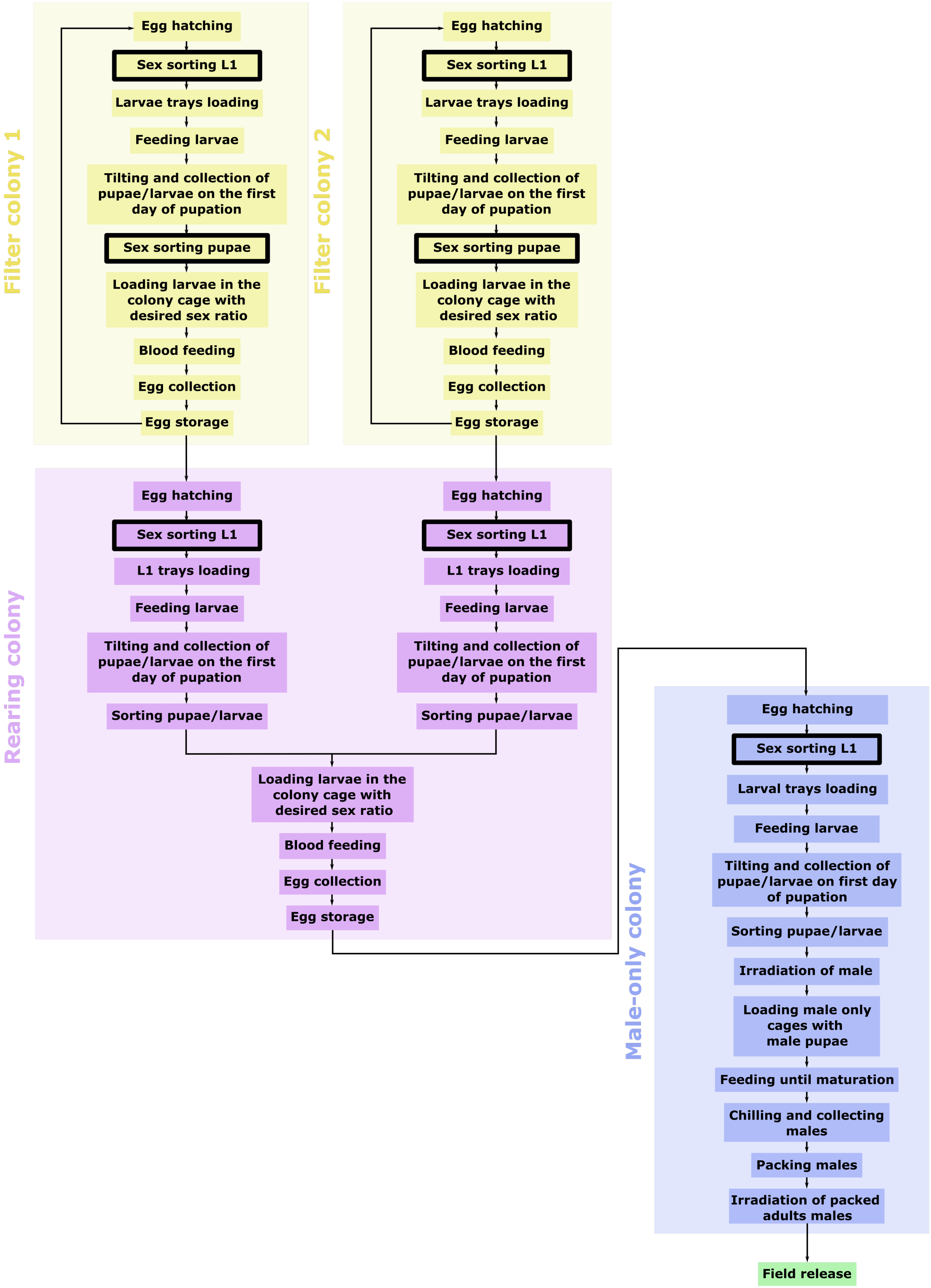
Filter colony 1
Egg hatching
Sex sorting L1
Larvae trays loading
Feeding larvae
Tilting and collection of pupae/larvae on the first day of pupation
Sex sorting pupae
Loading larvae in the colony cage with desired sex ratio
Blood feeding
Egg collection
Egg storage
Filter colony 2
Egg hatching
Sex sorting L1
Larvae trays loading
Feeding larvae
Tilting and collection of pupae/larvae on the first day of pupation
Sex sorting pupae
Loading larvae in the colony cage with desired sex ratio
Blood feeding
Egg collection
Egg storage
Rearing colony
Egg hatching
Sex sorting L1
L1 trays loading
Feeding larvae
Tilting and collection of pupae/larvae on the first day of pupation
Sorting pupae/larvae
Egg hatching
Sex sorting L1
L1 trays loading
Feeding larvae
Tilting and collection of pupae/larvae on the first day of pupation
Sorting pupae/larvae
Loading larvae in the colony cage with desired sex ratio
Blood feeding
Egg collection
Egg storage
Male-only colony
Egg hatching
Sex sorting L1
Larval trays loading
Feeding larvae
Tilting and collection of pupae/larvae on first day of pupation
Sorting pupae/larvae
Irradiation of male
Loading male only cages with male pupae
Feeding until maturation
Chilling and collecting males
Packing males
Irradiation of packed adults males
Field release

Simulation outputs

Insect production

Number of released males (M)	Size manual	Size auto	GSS	GSS-CS	GSS vs. Size manual	GSS-CS vs. Size manual
**Number of eggs to be hatched (M/day)**						
5	7.96	8.44	5.49	6.46	-2.46 (-31%)	-1.49 (-18.8%)
10	15.92	16.88	10.99	12.93	-4.93 (-31%)	-2.99 (-18.8%)
20	31.83	33.75	21.98	25.86	-9.86 (-31%)	-5.98 (-18.8%)
50	79.58	84.38	54.94	64.64	-24.64 (-31%)	-14.94 (-18.8%)
100	159.16	168.76	109.88	129.28	-49.29 (-31%)	-29.89 (-18.8%)
**Number of larvae in the mass-rearing facility (M)**						
5	32.98	34.96	11.56	17.82	-21.42 (-65%)	-15.16 (-46%)
10	65.95	69.93	23.00	35.53	-42.95 (-65.1%)	-30.42 (-46.1%)
20	131.91	139.86	46.01	71.06	-85.90 (-65.1%)	-60.84 (-46.1%)
50	329.77	349.65	114.59	176.90	-215.18 (-65.3%)	-152.86 (-46.4%)
100	659.53	699.30	228.96	353.48	-430.57 (-65.3%)	-306.05 (-46.4%)
**Number of adults in the mass-rearing facility (M)**						
5	4.84	4.84	3.63	4.44	-1.21 (-25%)	-0.40 (-8.3%)
10	9.28	9.68	6.45	7.66	-2.82 (-30.4%)	-1.61 (-17.4%)
20	18.15	18.96	12.91	15.33	-5.24 (-28.9%)	-2.82 (-15.6%)
50	44.77	47.59	31.46	37.51	-13.31 (-29.7%)	-7.26 (-16.2%)
100	89.14	94.78	62.92	75.02	-26.22 (-29.4%)	-14.12 (-15.8%)

Facility area

Number of released males (M)	Size manual	Size auto	GSS	GSS-CS	GSS vs. Size manual	GSS-CS vs. Size manual
**Mass rearing facility area (m2)**						
5	535	539	417	470	-118 (-22.1%)	-65 (-12.2%)
10	839	876	565	661	-274 (-32.6%)	-178 (-21.2%)
20	1,466	1,519	893	1,098	-573 (-39.1%)	-368 (-25.1%)
50	3,321	3,516	1,895	2,389	-1,427 (-43%)	-933 (-28.1%)
100	6,437	6,800	3,553	4,533	-2,884 (-44.8%)	-1,903 (-29.6%)
**Release facility area (m2)**						
5	174	174	174	174	0 (0%)	0 (0%)
10	228	228	228	228	0 (0%)	0 (0%)
20	338	338	338	338	0 (0%)	0 (0%)
50	667	667	667	667	0 (0%)	0 (0%)
100	1,215	1,215	1,215	1,215	0 (0%)	0 (0%)

Workload (both facilities)

Number of released males (M)	Size manual	Size auto	GSS	GSS-CS	GSS vs. Size manual	GSS-CS vs. Size manual
**Number of rearing labourers**						
5	40	42	28	32	-12 (-30%)	-8 (-20%)
10	76	79	48	57	-28 (-36.8%)	-19 (-25%)
20	151	156	95	110	-56 (-37.1%)	-41 (-27.2%)
50	368	385	230	269	-138 (-37.5%)	-99 (-26.9%)
100	733	766	453	531	-280 (-38.2%)	-202 (-27.6%)
**Number of QC technicians**						
5	4	4	2	3	-2 (-50%)	-1 (-25%)
10	6	6	3	4	-3 (-50%)	-2 (-33.3%)
20	14	14	8	9	-6 (-42.9%)	-5 (-35.7%)
50	36	37	21	25	-15 (-41.7%)	-11 (-30.6%)
100	72	75	41	50	-31 (-43.1%)	-22 (-30.6%)
**Number of maintenance officers**						
5	2	2	1	1	-1 (-50%)	-1 (-50%)
10	2	2	1	1	-1 (-50%)	-1 (-50%)
20	6	6	3	4	-3 (-50%)	-2 (-33.3%)
50	18	19	11	13	-7 (-38.9%)	-5 (-27.8%)
100	38	40	23	27	-15 (-39.5%)	-11 (-28.9%)
**Total number of staff for both facilities**						
5	55	57	39	45	-16 (-29.1%)	-10 (-18.2%)
10	102	105	65	78	-37 (-36.3%)	-24 (-23.5%)
20	200	206	127	147	-73 (-36.5%)	-53 (-26.5%)
50	483	504	303	353	-180 (-37.3%)	-130 (-26.9%)
100	957	1,000	588	692	-369 (-38.6%)	-265 (-27.7%)

Water requirements

Number of released males (M)	Size manual	Size auto	GSS	GSS-CS	GSS vs. Size manual	GSS-CS vs. Size manual
**Daily water requirements (L)**						
5	3,704	3,900	1,580	2,202	-2,124 (-57.3%)	-1,502 (-40.6%)
10	7,403	7,799	3,142	4,383	-4,261 (-57.6%)	-3,021 (-40.8%)
20	14,802	15,594	6,284	8,766	-8,518 (-57.5%)	-6,037 (-40.8%)
50	36,999	38,986	15,658	21,831	-21,341 (-57.7%)	-15,168 (-41%)
100	73,993	77,968	31,296	43,630	-42,698 (-57.7%)	-30,364 (-41%)

Construction budget

Number of released males (M)	Size manual	Size auto	GSS	GSS-CS	GSS vs. Size manual	GSS-CS vs. Size manual
**Construction cost of the mass rearing facility ($)**						
5	653,350	656,800	511,188	575,122	-142,163 (-21.8%)	-78,228 (-12%)
10	1,019,988	1,063,840	690,876	805,563	-329,112 (-32.3%)	-214,424 (-21%)
20	1,776,099	1,840,034	1,086,457	1,333,551	-689,642 (-38.8%)	-442,548 (-24.9%)
50	4,015,630	4,250,948	2,296,329	2,893,922	-1,719,301 (-42.8%)	-1,121,708 (-27.9%)

(continued)

Number of released males (M)	Size manual	Size auto	GSS	GSS-CS	GSS vs. Size manual	GSS-CS vs. Size manual
100	7,774,879	8,213,995	4,300,135	5,485,650	-3,474,744 (-44.7%)	-2,289,229 (-29.4%)
**Yearly depreciation of the mass rearing facility ($)**						
5	32,668	32,840	25,559	28,756	-7,108 (-21.8%)	-3,911 (-12%)
10	50,999	53,192	34,544	40,278	-16,456 (-32.3%)	-10,721 (-21%)
20	88,805	92,002	54,323	66,678	-34,482 (-38.8%)	-22,127 (-24.9%)
50	200,782	212,547	114,816	144,696	-85,965 (-42.8%)	-56,085 (-27.9%)
100	388,744	410,700	215,007	274,282	-173,737 (-44.7%)	-114,461 (-29.4%)
**Construction cost of the release facility ($)**						
5	237,901	237,901	237,901	237,901	0 (0%)	0 (0%)
10	311,603	311,603	311,603	311,603	0 (0%)	0 (0%)
20	459,006	459,006	459,006	459,006	0 (0%)	0 (0%)
50	901,214	901,214	901,214	901,214	0 (0%)	0 (0%)
100	1,638,082	1,638,082	1,638,082	1,638,082	0 (0%)	0 (0%)
**Yearly depreciation of the release facility ($)**						
5	11,895	11,895	11,895	11,895	0 (0%)	0 (0%)
10	15,580	15,580	15,580	15,580	0 (0%)	0 (0%)
20	22,950	22,950	22,950	22,950	0 (0%)	0 (0%)
50	45,061	45,061	45,061	45,061	0 (0%)	0 (0%)
100	81,904	81,904	81,904	81,904	0 (0%)	0 (0%)

Equipment budget

Number of released males (M)	Size manual	Size auto	GSS	GSS-CS	GSS vs. Size manual	GSS-CS vs. Size manual
**Mass rearing facility equipment cost ($)**						
5	1,093,510	1,831,030	1,247,100	1,448,810	153,590 (14%)	355,300 (32.5%)
10	1,791,920	3,249,980	1,913,010	2,314,060	121,090 (6.8%)	522,140 (29.1%)
20	3,260,960	6,055,430	3,238,590	4,103,040	-22,370 (-0.7%)	842,080 (25.8%)
50	7,534,210	14,563,840	7,270,080	9,493,760	-264,130 (-3.5%)	1,959,550 (26%)
100	14,751,890	28,653,800	13,933,530	18,352,250	-818,360 (-5.5%)	3,600,360 (24.4%)
**Mass rearing facility equipment yearly depreciation ($)**						
5	132,888	207,608	136,497	159,878	3,608 (2.7%)	26,990 (20.3%)
10	222,067	369,903	210,832	257,063	-11,235 (-5.1%)	34,997 (15.8%)
20	408,607	692,415	357,415	456,713	-51,192 (-12.5%)	48,107 (11.8%)
50	950,178	1,665,433	803,147	1,059,787	-147,032 (-15.5%)	109,608 (11.5%)
100	1,865,932	3,279,073	1,541,245	2,050,885	-324,687 (-17.4%)	184,953 (9.9%)
**Release facility equipment cost**						
5	88,508	88,508	88,508	88,508	0 (0%)	0 (0%)
10	141,016	141,016	141,016	141,016	0 (0%)	0 (0%)
20	246,032	246,032	246,032	246,032	0 (0%)	0 (0%)
50	561,080	561,080	561,080	561,080	0 (0%)	0 (0%)
100	1,086,076	1,086,076	1,086,076	1,086,076	0 (0%)	0 (0%)
**Release facility equipment yearly depreciation ($)**						
5	17,660	17,660	17,660	17,660	0 (0%)	0 (0%)
10	29,121	29,121	29,121	29,121	0 (0%)	0 (0%)
20	52,041	52,041	52,041	52,041	0 (0%)	0 (0%)
50	120,803	120,803	120,803	120,803	0 (0%)	0 (0%)
100	235,386	235,386	235,386	235,386	0 (0%)	0 (0%)
**Total equipment cost**						
5	1,182,018	1,919,538	1,335,608	1,537,318	153,590 (13%)	355,300 (30.1%)

(continued)

Number of released males (M)	Size manual	Size auto	GSS	GSS-CS	GSS vs. Size manual	GSS-CS vs. Size manual
10	1,932,936	3,390,996	2,054,026	2,455,076	121,090 (6.3%)	522,140 (27%)
20	3,506,992	6,301,462	3,484,622	4,349,072	-22,370 (-0.6%)	842,080 (24%)
50	8,095,290	15,124,920	7,831,160	10,054,840	-264,130 (-3.3%)	1,959,550 (24.2%)
100	15,837,966	29,739,876	15,019,606	19,438,326	-818,360 (-5.2%)	3,600,360 (22.7%)
**Total equipment yearly depreciation ($)**						
5	150,549	225,269	154,157	177,539	3,608 (2.4%)	26,990 (17.9%)
10	251,187	399,024	239,952	286,184	-11,235 (-4.5%)	34,997 (13.9%)
20	460,648	744,456	409,456	508,755	-51,192 (-11.1%)	48,107 (10.4%)
50	1,070,982	1,786,237	923,950	1,180,590	-147,032 (-13.7%)	109,608 (10.2%)
100	2,101,317	3,514,459	1,776,631	2,286,271	-324,687 (-15.5%)	184,953 (8.8%)

Diet and consumables yearly cost

Number of released males (M)	Size manual	Size auto	GSS	GSS-CS	GSS vs. Size manual	GSS-CS vs. Size manual
**Yearly diet cost**						
5	30,720	32,346	13,376	18,742	-17,344 (-56.5%)	-11,978 (-39%)
10	61,388	64,692	26,599	37,327	-34,789 (-56.7%)	-24,061 (-39.2%)
20	122,723	129,331	53,169	74,585	-69,553 (-56.7%)	-48,138 (-39.2%)
50	306,728	323,340	132,452	185,717	-174,276 (-56.8%)	-121,011 (-39.5%)
100	613,402	646,628	264,721	371,166	-348,681 (-56.8%)	-242,236 (-39.5%)
**Yearly consumables cost**						
5	8,110	8,435	4,641	5,714	-3,469 (-42.8%)	-2,396 (-29.5%)
10	15,554	16,214	8,596	10,741	-6,958 (-44.7%)	-4,812 (-30.9%)
20	30,441	31,763	16,531	20,814	-13,911 (-45.7%)	-9,628 (-31.6%)
50	75,105	78,427	40,250	50,903	-34,855 (-46.4%)	-24,202 (-32.2%)
100	149,544	156,189	79,807	101,096	-69,736 (-46.6%)	-48,447 (-32.4%)
**Yearly cost for diet and consumables**						
5	38,830	40,781	18,017	24,456	-20,813 (-53.6%)	-14,374 (-37%)
10	76,941	80,906	35,194	48,069	-41,747 (-54.3%)	-28,873 (-37.5%)
20	153,164	161,094	69,700	95,399	-83,464 (-54.5%)	-57,765 (-37.7%)
50	381,832	401,768	172,701	236,619	-209,131 (-54.8%)	-145,213 (-38%)
100	762,946	802,817	344,528	472,263	-418,418 (-54.8%)	-290,683 (-38.1%)

Estimate of the total yearly cost for construction, equipment, diet and consumables

Number of released males (M)	Size manual	Size auto	GSS	GSS-CS	GSS vs. Size manual	GSS-CS vs. Size manual
5	233,941	310,785	209,629	242,646	-24,313 (-10.4%)	8,705 (3.7%)
10	394,708	548,703	325,271	390,111	-69,438 (-17.6%)	-4,597 (-1.2%)
20	725,567	1,020,502	556,430	693,781	-169,138 (-23.3%)	-31,786 (-4.4%)
50	1,698,656	2,445,613	1,256,529	1,606,966	-442,128 (-26%)	-91,690 (-5.4%)
100	3,334,911	4,809,880	2,418,070	3,114,720	-916,842 (-27.5%)	-220,192 (-6.6%)

Initial parameters

Weekly flying male production (production) [5, 10, 20, 50, 100] M males

Parameters	Size manual	Size auto	GSS	GSS-CS
**Production goals**				
Weekly pupal production (Mpp/week)	production / 0.95	production / 0.95	production / 0.95	production / 0.95
Rearing efficienty				
Number of weeks with releases per year	52	52	52	52
**Biological parameters**				
Egg hatching rate	0.85	0.85	0.85	0.85
Sex ratio (M/(F+M))	0.5	0.5	0.5	0.5
Male pupae recovery after first sorting	0.65	0.65	0.65	0.65
Male pupae recovery after second sorting	0.15	0.15	0.15	0.15
Male pupae recovery after third sorting	0.04	0.04	0.04	0.04
Female pupae recovery after first sorting	0.32	0.32	0.32	0.32
Female pupae recovery after second sorting	0.35	0.35	0.35	0.35
Female pupae recovery after third sorting	0.36	0.36	0.36	0.36
Number of tilting or sex sorting operations for MO	1	1	1	1
**Number of tilting or sex sorting operations for colony**	**2**	**2**	**1**	**1**
Survival pupae to flying males	0.95	0.95	0.95	0.95
Survival pupae to flying females	0.95	0.95	0.95	0.95
Average number of eggs per females for the 1st gonotrophic cycle	30	30	30	30
Average number of eggs per females for the 2nd gonotrophic cycle	20	20	20	20
Average number of eggs per females for the 3rd gonotrophic cycle	10	10	10	10
Life cycle information				
Pre oviposition period (days)	8	8	8	8
Duration of gonotrophic cycle (days)	7	7	7	7
Number of gonotrophic cycles colonies	3	3	3	3
Duration of males holding before release (days)	4	4	4	4
Duration of the larval cycle Colony (days)	8	8	8	8
Egg density (eggs/mL)	91000	91000	91000	91000
Pupa density (pupae/L)	333333	333333	333333	333333
**Capacity of main equipment**				
Larval trays				
Density of larvae in colony trays (larvae/mL)	3	3	3	3
Depth of colony larval tray (cm)	1	1	1	1
Larval tray length (cm)	60	60	60	60
Larval tray width (cm)	100	100	100	100
Height of tray columns (cm)	170	170	170	170
Free space between trays (cm)	2.5	2.5	2.5	2.5

(continued)

Parameters	Size manual	Size auto	GSS	GSS-CS
MO cages				
Vertical resting space in MO cages (cm2/adult)	1	1	1	1
MO cage compartment (cm2 per cage)	12	12	12	12
MO cage height (cm)	25	25	25	25
MO cage width (cm)	10	10	10	10
MO cage length (cm)	6	6	6	6
Colony cages				
Vertical resting place for colony cages (cm2/adult)	1.2	1.2	1.2	1.2
Net colony cages height (cm)	100	100	100	100
Net colony cages width (cm)	20	20	20	20
Net colony cages length (cm)	90	90	90	90
**Rearing task schedule**				
Frequency of blood feeding (days)	3	3	3	3
Frequency of egg collection (days)	3	3	3	3
Number of feeding rounds per blood membrane	5	5	5	5
Frequency of colony cage washing (holding cycle)	1	1	1	1
Frequency of MO cage washing (holding cycle)	1	1	1	1
Increase factor mother colony	1.08	1.08	1.08	1.08
**Diet formula**				
Larval diet				
Beef liver powder (%)	0	0	0	0
Tuna meal (%)	50	50	50	50
Brewer yeast (%)	15	15	15	15
BSF yeast (%)	35	35	35	35
Component 5 (%)	0	0	0	0
Component 6 (%)	0	0	0	0
Concentration in water (g/mL)	4	4	4	4
Ration per one larva on day 1 (mg ingredients/larva)	0.66	0.66	0.66	0.66
Ration per one larva on day 2 (mg ingredients/larva)	0	0	0	0
Ration per one larva on day 3 (mg ingredients/larva)	0	0	0	0
Ration per one larva on day 4 (mg ingredients/larva)	0.66	0.66	0.66	0.66
Ration per one larva on day 5 (mg ingredients/larva)	0.44	0.44	0.44	0.44
Ration per one larva on day 6 (mg ingredients/larva)	0.66	0.66	0.66	0.66
Ration per one larva on day 7 after 1st sorting (mg ingredients/larva)	0	0	0	0
Ration per one larva on day 8 after 2nd sorting (mg ingredients/larva)	0	0	0	0
Adult diet				
Sugar percentage in adult diets	10	10	10	10
Weight of sugar per colony cage (kg/cage)	0.03	0.03	0.03	0.03
Weight of sugar per MO cage (kg/cage)	0.1	0.1	0.1	0.1
Replenishment of water in adult cages (days)	5	5	5	5

(continued)

Parameters	Size manual	Size auto	GSS	GSS-CS
Average Blood intake per female (mg/female/intake)	1.5	1.5	1.5	1.5
**Storage of ingredients**				
Beef liver powder storage time (days)	90	90	90	90
Beef liver powder storage bags per m3	6	6	6	6
Beef liver powder (kg/unit)	25	25	25	25
Beef liver powder height (cm)	1	1	1	1
Tuna meal storage time (days)	90	90	90	90
Tuna meal storage bags per m3	6	6	6	6
Tuna meal (kg/unit)	25	25	25	25
Tuna meal height (cm)	1	1	1	1
Brewer yeast storage time (days)	90	90	90	90
Brewer yeast storage bags per m3	6	6	6	6
Brewer yeast (kg/unit)	25	25	25	25
Brewer yeast height (cm)	1	1	1	1
BSF yeast storage time (days)	90	90	90	90
BSF yeast storage bags per m3	6	6	6	6
BSF yeast (kg/unit)	25	25	25	25
BSF yeast height (cm)	1	1	1	1
Component 5 storage time (days)	90	90	90	90
Component 5 storage bags per m3	6	6	6	6
Component 5 (kg/unit)	25	25	25	25
Component 5 height (cm)	1	1	1	1
Component 6 storage time (days)	90	90	90	90
Component 6 storage bags per m3	6	6	6	6
Component 6 (kg/unit)	25	25	25	25
Component 6 height (cm)	1	1	1	1
Total sugar for adult diet storage time (days)	90	90	90	90
Total sugar for adult diet storage bags per m3	6	6	6	6
Total sugar for adult diet (kg/unit)	50	50	50	50
Total sugar for adult diet height (cm)	1	1	1	1
**Rearing equipment**				
Oversize factors				
Oversize factor larval trays	1.3	1.3	1.3	1.3
Oversize factor racks	1.2	1.2	1.2	1.2
Oversize factor cages	1.2	1.2	1.2	1.2
Oversize factor RM cages	1.3	1.3	1.3	1.3
Irradiators				
Number of pupae per irradiation canister	375000	375000	375000	375000
Duration of one irradiation operation (hour)	0.17	0.17	0.17	0.17
Irradiator max time (h/day)	12	12	12	12
Irradiator backup	0	0	0	0
L1 sex sorter				
L1 sex sorter male recovery	0.696	0.696	0.696	0.696
L1 sex sorter female recovery	0.696	0.696	0.696	0.696
L1 sex sorter throwput (M/hour)	0.133	0.133	0.133	0.133
L1 sex sorter max time (h/day)	5	5	5	5
L1 sex sorter backup	1	1	1	1
Pupal sorter				
**Pupal sex sorter male recovery**	**0.427**	**0.403**	**0.403**	**0.403**
Pupal sex sorter female recovery	0.3806	0.3806	0.3806	0.3806

(continued)

Parameters	Size manual	Size auto	GSS	GSS-CS
Pupal sorter throwput (Mpp/h)	0.05	0.05	0.05	0.05
Pupal sorter max time (h/day)	5	5	5	5
Pupal sorter backup	1	1	1	1
Male recovery for GSS filter double check	0.9	0.9	0.9	0.9
L1 counter				
L1 counter capacity (tray/h)	20	20	20	20
L1 counter max time	4	4	4	4
L1 counter backup	1	1	1	1
Larval diet mixer				
Larval diet mixer capacity (trays/h)	40	40	40	40
Larval diet mixer duration per batch (h)	0.25	0.25	0.25	0.25
Larval diet mixer max time (h/day)	3	3	3	3
Larval diet mixer backup	0	0	0	0
Adult diet mixer				
Adult diet mixer capacity (L/operation)	20	20	20	20
Adult diet mixer duration per batch (h)	0.25	0.25	0.25	0.25
Adult diet mixer max time (h/day)	3	3	3	3
Adult diet mixer backup	0	0	0	0
Larval diet feeder				
Larval feeder capacity (trays/h)	45	45	45	45
Larval feeder max time (h/day)	4	4	4	4
Larval feeder backup	1	1	1	1
Blood feeder				
Blood feeder capacity (cages/h)	2	2	2	2
Blood feeder max time (h/day)	6	6	6	6
Blood feeder backup	1	1	1	1
Tray washer				
Tray washer capacity (trays/h)	200	200	200	200
Tray washer max time (h/day)	6	6	6	6
Tray washer backup	0	0	0	0
Cage washer				
Cage washer capacity (cages/h)	2.4	2.4	2.4	2.4
Cage washer max time (h/day)	6	6	6	6
Cage washer backup	0	0	0	0
MO cage washer				
MO cage washer capacity (cages/h)	12	12	12	12
MO cage washer max time (h/day)	6	6	6	6
MO cage washer backup	0	0	0	0
**Water requirement**				
Water per tray wash (L/tray)	0.5	0.5	0.5	0.5
Water per cage wash (L/cage)	2	2	2	2
Water per MO cage wash (L/cage)	3	3	3	3
Percentage of water for room cleaning (%)	0.4	0.4	0.4	0.4
**Floor area calculation**				
Mass rearing facility				
Rack oversize factor	2	2	2	2

(continued)

Parameters	Size manual	Size auto	GSS	GSS-CS
Colony cage oversize factor	2	2	2	2
Storage oversize factor	3	3	3	3
Offices size per staff (m2/staff)	5	5	5	5
WC size per staff (m2/staff)	3	3	3	3
Percentage corridors (%)	0.1	0.1	0.1	0.1
Diet preparation area (m2)	10	10	10	10
Tray washing area (m2)	15	15	15	15
Cage washing area (m2)	15	15	15	15
QC lab area (m2)	20	20	20	20
Workshop area (m2)	20	20	20	20
Warehouse area (m2)	20	20	20	20
Office staff (m2)	6	6	6	6
WC staff (m2)	10	10	10	10
Realese facility				
MO cage oversize factor	2	2	2	2
MO cages loading area (m2)	15	15	15	15
MO QC lab area (m2)	20	20	20	20
MO adult packaging area (m2)	15	15	15	15
MO diet preparation area (m2)	15	15	15	15
MO Warehouse area (m2)	10	10	10	10
MO office staff (m2)	2	2	2	2
MO wc staff (m2)	6	6	6	6
**Construction cost**				
Cost m2 office labs wc irradiation corridor ($/m2)	1500	1500	1500	1500
Cost m2 rearing rooms ($/m2)	1200	1200	1200	1200
Cost m2 storage warehouse ($/m2)	1000	1000	1000	1000
Expected lifespan mass rearing facility (years)	20	20	20	20
Cost m2 chilling room ($/m2)	2000	2000	2000	2000
Expected lifespan release facility (years)	20	20	20	20
**Workload**				
Mass rearing facility				
Egg hatching work rate (h/day)	2	2	2	2
Tray hanging work rate (h/rack)	0.45	0.45	0.45	0.45
L1 dosage work rate (h/rack)	0.25	0.25	0.25	0.25
L4 loading work rate (h/rack)	0.05	0.05	0.05	0.05
Larval feeding work rate (h/rack)	0.25	0.25	0.25	0.25
Tray tilting work rate (h/rack)	1	1	1	1
Insect packing for irradiation work rate (h/canister)	0	0	0	0
Colony cage loading work rate (h/cage)	0.2	0.2	0.2	0.2
Colony cage bloodfeeding work rate (h/cage)	0.05	0.05	0.05	0.05
Egg collection work rate (h/cage)	0.05	0.05	0.05	0.05
Egg storage work rate (h/day)	0	0	0	0
Larval diet prep work rate (hour/batch)	0.15	0.15	0.15	0.15
Adult diet prep work rate (hour/batch)	0.1	0.1	0.1	0.1
Blood collection weekly	1	1	1	1
Blood collection work rate (hour/collection)	5	5	5	5
Blood doses prep work rate (hour/dose)	0.1	0.1	0.1	0.1
Net working time per staff per day (h/day)	7.5	7.5	7.5	7.5

(continued)

Parameters	Size manual	Size auto	GSS	GSS-CS
Realese facility				
MO cage loading work rate (h/cage)	0.1	0.1	0.1	0.1
MO cage chilling work rate (h/cage)	0.1	0.1	0.1	0.1
MO adult collection work rate (hour/cage)	0.15	0.15	0.15	0.15
MO adult packing for release work rate (hour/cage)	0.1	0.1	0.1	0.1
Majoring factors				
Majoring factor labourer	1.7	1.7	1.7	1.7
Majoring factor team leader	1.5	1.5	1.5	1.5
Majoring factor QC manager	1	1	1	1
Majoring factor QC technician	1	1	1	1
Majoring factor maintenance manager	1	1	1	1
Majoring factor maintenance officer	1	1	1	1
Majoring factor admin assistant	1	1	1	1
Majoring factor manager	1	1	1	1
QC managers needed mass rearing	1	1	1	1
Manager needed mass rearing	1	1	1	1
**Equipment budget**				
Mass rearing facility				
Larval trays unit price ($)	80	80	80	80
Larval trays life expectancy	6	6	6	6
Racks for larval trays unit price ($)	7000	7000	7000	7000
Racks for larval trays life expectancy (years)	10	10	10	10
Cages for Colonies unit price ($)	350	350	350	350
Cages for Colonies life expectancy (years)	10	10	10	10
Irradiator unit price ($)	250000	250000	250000	250000
Irradiator life expectancy (years)	10	10	10	10
L1 Sex sorter unit price ($)	50000	50000	50000	50000
L1 Sex sorter life expectancy (years)	10	10	10	10
**Pupal sex sorter unit price ($)**	**40000**	**40000**	**40000**	**40000**
Pupal sex sorter life expectancy (years)	10	10	10	10
L1 counter unit price ($)	2000	2000	2000	2000
L1 counter life expectancy (years)	5	5	5	5
Larval diet mixer unit price ($)	3000	3000	3000	3000
Larval diet mixer life expectancy (years)	5	5	5	5
Adult diet mixer unit price ($)	2000	2000	2000	2000
Adult diet mixer life expectancy (years)	6	6	6	6
Larval diet feeder unit price ($)	3000	3000	3000	3000
Larval diet feeder life expectancy (years)	5	5	5	5
Blood feeders unit price ($)	2000	2000	2000	2000
Blood feeders life expectancy (years)	5	5	5	5
Tray washing machine in mass rearing facility unit price ($)	20000	20000	20000	20000

(continued)

Parameters	Size manual	Size auto	GSS	GSS-CS
Tray washing machine in mass rearing facility life expectancy (years)	6	6	6	6
Cage washing machine in mass rearing facility unit price ($)	20000	20000	20000	20000
Cage washing machine in mass rearing facility life expectancy (years)	6	6	6	6
Equipment for basic QC lab number	1	1	1	1
Equipment for basic QC lab unit price ($)	12000	12000	12000	12000
Equipment for basic QC lab life expectancy (years)	5	5	5	5
Workshop equipment number	1	1	1	1
Workshop equipment unit price ($)	25000	25000	25000	25000
Workshop equipment life expectancy (years)	10	10	10	10
Blood storage number	1	1	1	1
Blood storage unit price ($)	5000	5000	5000	5000
Blood storage life expectancy (years)	6	6	6	6
Realese facility				
Cages for Release Males unit price ($)	84	84	84	84
Cages for Release Males life expectancy (years)	4	4	4	4
Cage washing machine in release facility unit price ($)	20000	20000	20000	20000
Cage washing machine in release facility life expectancy (years)	6	6	6	6
Equipment for basic QC lab MO number	1	1	1	1
Equipment for basic QC lab MO unit price ($)	6000	6000	6000	6000
Equipment for basic QC lab MO life expectancy (years)	5	5	5	5
Workshop equipment MO number	1	1	1	1
Workshop equipment MO unit price ($)	10000	10000	10000	10000
Workshop equipment MO life expectancy (years)	6	6	6	6
Beef liver powder unit cost ($/kg)	80	80	80	80
**Diet and consumable costs**				
Tuna meal unit cost ($/kg)	0.7	0.7	0.7	0.7
Brewer yeast unit cost ($/kg)	8.5	8.5	8.5	8.5
BSF powder unit cost ($/kg)	8.5	8.5	8.5	8.5
Component 5 unit cost ($/kg)	0	0	0	0
Component 6 unit cost ($/kg)	0	0	0	0
Water including the initial load of trays unit cost ($/L)	3	3	3	3
Sugar for adult colony unit cost ($/kg)	1	1	1	1
Blood for adult diet unit cost ($/L)	2	2	2	2
Radiation dosimeters unit cost ($)	1.5	1.5	1.5	1.5
Consumables without inventoring unit cost ($)	0.2	0.2	0.2	0.2
