# Supplementary material for: Combining two genetic sexing strains allows sorting of non-transgenic males for Aedes genetic control
Source: Commun Biol. 2023 Jun 16;6:646. doi: 10.1038/s42003-023-05030-7 (PMC10275924; doi:10.1038/s42003-023-05030-7)
Supplement: Supplementary file 8 — Reporting summary [file 42003_2023_5030_MOESM8_ESM.pdf]

nature portfolio

Corresponding author(s): Célia Lutrat & Eric Marois

Last updated by author(s): Jun 2, 2023

Reporting Summary

Nature Portfolio wishes to improve the reproducibility of the work that we publish. This form provides structure for consistency and transparency in reporting. For further information on Nature Portfolio policies, see our Editorial Policies and the Editorial Policy Checklist.

Statistics

For all statistical analyses, confirm that the following items are present in the figure legend, table legend, main text, or Methods section.

n/a	Confirmed	
☐	☒	The exact sample size (*n*) for each experimental group/condition, given as a discrete number and unit of measurement
☐	☒	A statement on whether measurements were taken from distinct samples or whether the same sample was measured repeatedly
☐	☒	The statistical test(s) used AND whether they are one- or two-sided<br>*Only common tests should be described solely by name; describe more complex techniques in the Methods section.*
☒	☐	A description of all covariates tested
☐	☒	A description of any assumptions or corrections, such as tests of normality and adjustment for multiple comparisons
☐	☒	A full description of the statistical parameters including central tendency (e.g. means) or other basic estimates (e.g. regression coefficient) AND variation (e.g. standard deviation) or associated estimates of uncertainty (e.g. confidence intervals)
☐	☒	For null hypothesis testing, the test statistic (e.g. *F*, *t*, *r*) with confidence intervals, effect sizes, degrees of freedom and *P* value noted<br>*Give P values as exact values whenever suitable.*
☒	☐	For Bayesian analysis, information on the choice of priors and Markov chain Monte Carlo settings
☒	☐	For hierarchical and complex designs, identification of the appropriate level for tests and full reporting of outcomes
☒	☐	Estimates of effect sizes (e.g. Cohen's *d*, Pearson's *r*), indicating how they were calculated

Our web collection on statistics for biologists contains articles on many of the points above.

Software and code

Policy information about availability of computer code

Data collection	COPAS measurements were performed using Union Biometrica BIOSORT software.
Data analysis	Data analysis was performed on R version 4.1.0.<br>All custom codes used to analyse the data have been permanently deposited on Zenodo under DOI 10.5281/zenodo.7997430.

For manuscripts utilizing custom algorithms or software that are central to the research but not yet described in published literature, software must be made available to editors and reviewers. We strongly encourage code deposition in a community repository (e.g. GitHub). See the Nature Portfolio guidelines for submitting code & software for further information.

Data

Policy information about availability of data

All manuscripts must include a data availability statement. This statement should provide the following information, where applicable:

- Accession codes, unique identifiers, or web links for publicly available datasets
- A description of any restrictions on data availability
- For clinical datasets or third party data, please ensure that the statement adheres to our policy

All plasmid sequences are available in Supplementary Data 4. Plasmids are available from Addgene under reference numbers #173666, #183903, #183904, #183911, #183912, #183913, #183914 and #183966. Mosquito strains are available upon request from EM. All data needed to evaluate the conclusions in the paper are present in the paper and/or in the Supplementary Information. Datasets can also be downloaded from Zenodo.

Research involving human participants, their data, or biological material

Policy information about studies with human participants or human data. See also policy information about sex, gender (identity/presentation), and sexual orientation and race, ethnicity and racism.

Reporting on sex and gender	NA
Reporting on race, ethnicity, or other socially relevant groupings	NA
Population characteristics	NA
Recruitment	NA
Ethics oversight	NA

Note that full information on the approval of the study protocol must also be provided in the manuscript.

Field-specific reporting

Please select the one below that is the best fit for your research. If you are not sure, read the appropriate sections before making your selection.

☒ Life sciences ☐ Behavioural & social sciences ☐ Ecological, evolutionary & environmental sciences

For a reference copy of the document with all sections, see nature.com/documents/nr-reporting-summary-flat.pdf

Life sciences study design

All studies must disclose on these points even when the disclosure is negative.

Sample size	No sample size pre-calculation was performed. Sample sizes were chosen as large as possible depending on mosquito availability and insectary maintenance capacity.
Data exclusions	No data were excluded.
Replication	All experiments were performed on large mosquito pools and repeated on independent pools at least N=3 times. To ensure maximum reproducibility, no data was ever excluded.
Randomization	NA
Blinding	NA

Reporting for specific materials, systems and methods

We require information from authors about some types of materials, experimental systems and methods used in many studies. Here, indicate whether each material, system or method listed is relevant to your study. If you are not sure if a list item applies to your research, read the appropriate section before selecting a response.

Materials & experimental systems

n/a	Involved in the study	
☒	☐	Antibodies
☒	☐	Eukaryotic cell lines
☒	☐	Palaeontology and archaeology
☐	☒	Animals and other organisms
☒	☐	Clinical data
☒	☐	Dual use research of concern
☒	☐	Plants

Methods

n/a	Involved in the study	
☒	☐	ChIP-seq
☐	☒	Flow cytometry
☒	☐	MRI-based neuroimaging

Animals and other research organisms

Policy information about studies involving animals; ARRIVE guidelines recommended for reporting animal research, and Sex and Gender in Research

Laboratory animals	Aedes albopictus and Aedes aegypti mosquitoes were used for all experiments. Mice were used for mosquito bloodfeeding.
Wild animals	The study did not involve wild animals.
Reporting on sex	NA
Field-collected samples	The study did not involve samples collected from the field.
Ethics oversight	Mice use and rearing was approved by CREMEAS ethics comittee.

Note that full information on the approval of the study protocol must also be provided in the manuscript.

Flow Cytometry

Plots

Confirm that:

☒ The axis labels state the marker and fluorochrome used (e.g. CD4-FITC).

☒ The axis scales are clearly visible. Include numbers along axes only for bottom left plot of group (a 'group' is an analysis of identical markers).

☒ All plots are contour plots with outliers or pseudocolor plots.

☒ A numerical value for number of cells or percentage (with statistics) is provided.

Methodology

Sample preparation	COPAS is a device allowing large-object flow cytometry. We used it for sorting mosquito larvae. Eggs were hatched by placing them into water and applying a 30min vacuum. Neonate larvae were then directly introduced in the sample tank with water, their fluorescence being endogenous.
Instrument	COPAS SELECT (Union Biometrica) with 500µm flowcell.
Software	COPAS SELECT provided software: Biosort.
Cell population abundance	Several hundred to several thousands of larvae were sorted at once. Exact larva population was measured by the software. Purity was assessed on the Ext/TOF graph, and non-larvae were excluded from the gate region.
Gating strategy	Larvae with distinct fluorescence intensity formed distinct dot clouds. Following a first run of the sample in "Aquire" mode, the gate and sort regions were defined so that all dots contained in the gate region were larvae and all dots in the sort region were larvae of the desired fluorescence. When necessary, trial sortings coud be performed on a sub-sample and controlled visually under a fluorescence binocular microscope in order to refine the sort region.

☒ Tick this box to confirm that a figure exemplifying the gating strategy is provided in the Supplementary Information.
